# Antiretroviral regimen and sex-specific metabolic heterogeneity in pediatric HIV

**DOI:** 10.1016/j.isci.2026.116865

**Published:** 2026-07-20

**Authors:** Chandre Herbert, Louise Kuhn, Nicole H. Tobin, Fan Li, Renate Strehlau, Faeezah Patel, Tian Wang, Shuang Wang, Grace M. Aldrovandi, Caroline T. Tiemessen

**Affiliations:** 1 National Institutes for Communicable Diseases, Faculty of Health Sciences, University of the Witwatersrand, Johannesburg, South Africa; 2 Gertrude H. Sergievsky Center, Vagelos College of Physicians and Surgeons, Department of Epidemiology, Mailman School of Public Health, Columbia University Irving Medical Center, New York, NY, USA; 3 Department of Pediatrics, University of California, Los Angeles, Los Angeles, CA, USA; 4 VIDA Nkanyezi Research Unit, Rahima Moosa Mother and Child Hospital, Department of Paediatrics and Child Health, Faculty of Health Sciences, University of the Witwatersrand, Johannesburg, South Africa; 5 Wits RHI, Faculty of Health Sciences, University of the Witwatersrand, Johannesburg, South Africa

**Keywords:** perinatally acquired HIV, children living with HIV, antiretroviral therapy, virally suppressed, prepubescent, sexual dimorphism, metabolomics, metabotypes, mitochondria, HIV cure

## Abstract

Host metabolism is increasingly recognized as relevant in HIV remission and cure research yet remains understudied in children living with perinatally acquired human immunodeficiency virus (CLWH). The relation between clinical factors and plasma metabolic profiles, generated by untargeted ultra high-performance liquid chromatography/tandem mass spectrometry, of pre-pubescent, virally suppressed CLWH (*n* = 155) and uninfected controls (UCs, *n* = 155) was investigated. The specific antiretroviral treatment (ART) regimen dominated the plasma metabolic profile and significantly influenced metabolic sexual dimorphism in CLWH. Two CD4%-associated metabotypes among well-controlled CLWH on the same regimen suggested divergent, sub-clinical responses to ART. Phospholipids were most frequently able to predict HIV status independent of treatment regimen. Metabolic biomarkers are unlikely to reliably predict outcomes in CLWH across regimens, if not curated for ART-induced divergence. Thus, grouping of treated individuals in metabolic studies should be done with careful consideration for the ART regimen used.

## Highlights

• ART regimen is a major driver of plasma metabolic variation in CLWH
• Sex-based metabolic signatures in children are shaped by HIV and ART
• CD4%-associated metabotypes suggest divergent responses to the same ART
• Findings have implications for study design and interpretation of metabolomics data

## Graphical abstract

**Figure:**
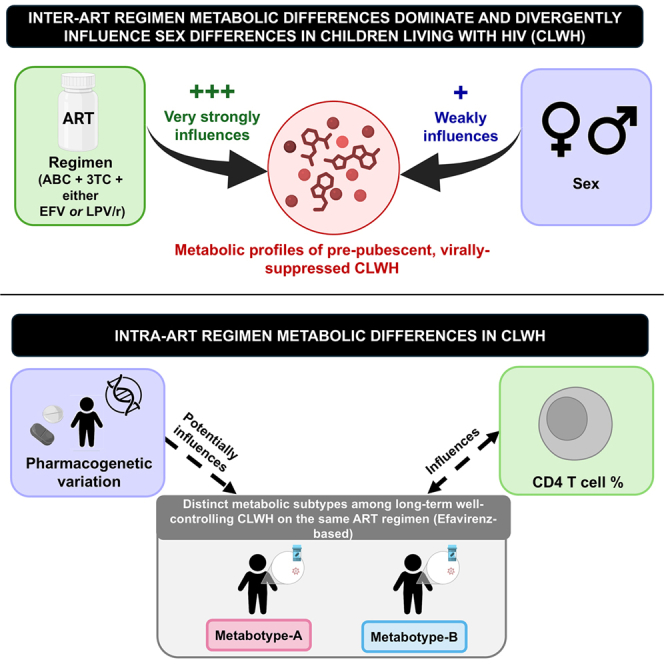

## Teaser

Pharmacology; Human metabolism; Systems biology

## Introduction

Early initiation of antiretroviral therapy (ART) in children living with perinatally acquired human immunodeficiency virus (CLWH) has enabled many to survive into adulthood.^1^ However, no widely applicable remission or cure for human immunodeficiency virus (HIV) infection exists. Consequently, CLWH face lifelong exposure to potentially toxic antiretrovirals (ARVs), a persisting viral reservoir, and associated chronic inflammation. Efforts to understand host factors influencing HIV persistence, particularly in genetics, immunology, and more recently, immunometabolism, have increased.^2^^,^^3^ Yet, the metabolic effects of HIV and ART remain poorly understood, especially in CLWH, due to cohort diversity and limited reproducibility. Understanding this variability is essential to evaluating biomarkers and cure strategies effectively.

Most HIV metabolic studies broadly characterize people living with HIV (PLWH) as treated or untreated, are of small sample size, and often overlook variables such as ART regimen, sex, infection and treatment timing, and reservoir size - factors critical for remission and cure research. ARVs impact not only viral replication but also metabolic enzymes, transporters, and other human proteins,^4^ with varying effects across classes and within drug classes.^5^ These interactions may be modified by genetic and treatment adherence-related factors, contributing to toxicity.^6^

Perinatal HIV infection presents unique metabolic challenges distinct from adult-acquired infection due to developmental changes. Data on the long-term outcomes of CLWH are emerging,^7^ and some have shown that sub-clinical abnormalities persist in CLWH on long-term ART, which may only cross the threshold of clinical observation years later.^8^^,^^9^ While ART success is clinically measured by viral suppression and tolerability, its broader physiological impacts, even if subclinical,^10^ require greater attention to improve long-term health and inform cure strategies.

Given the fundamental role of metabolism in essentially all biological processes, and especially immune responses, the effects of ART must be considered in studies of PLWH. Sexually dimorphic responses in HIV acquisition, pathogenesis, and treatment are increasingly recognized,^11^^,^^12^ but understudied from a metabolic perspective.^11^ White men are overrepresented in analytical treatment interruption (ATIs) studies, skewing current understanding in terms of ethnicity and sex.^13^ As pediatric ATIs become more common with recommendations recently published,^14^ understanding participants’ physiology is crucial to ensure safety. Metabolic data on CLWH are limited and heterogeneous, with only two studies conducted in school-aged children within a relatively narrow age range; however, their differing aims make direct comparison difficult.^15^^,^^16^

To address these gaps, we investigated the metabolic effects of non-nucleoside reverse transcriptase inhibitor (NNRTI) or protease inhibitor (PI)-based ART regimens and sex in pre-pubescent, virally suppressed CLWH from the Childhood HAART Alterations in Normal Growth, Genes, and Aging Evaluation Study (CHANGES) cohort, using untargeted metabolomics. ART regimen significantly influenced metabolic variation and the manifestation of sex differences. Given this prominent effect of ART, a data-driven approach identified distinct metabotypes among long-term well-controlled (LTWC) CLWH on the same regimen, highlighting interindividual variability. Finally, we identified metabolites that distinguish CLWH from uninfected controls (UCs), regardless of regimen. These findings contribute to a more nuanced understanding of HIV-associated metabolism and support refinements in study designs for future research.

## Results

### Setting the stage: Strategy and clinical characteristics

**Figure 1:**
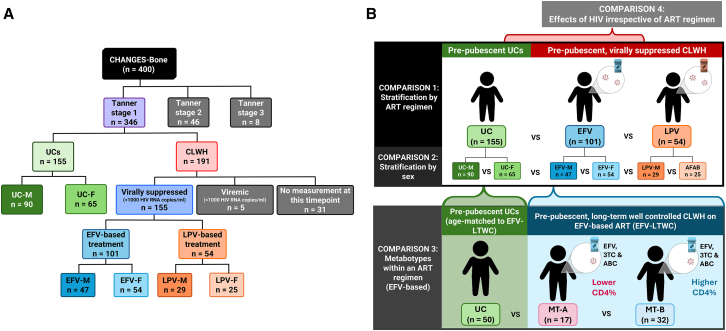
Participant selection and the stratification strategy (A) Prepubescent virally suppressed CLWH and uninfected controls were selected from the CHANGES cohort. CLWH without viral load data at this time point were excluded. (B) Comparisons were made by (1) ART regimen, (2) sex (in UCs and CLWH), and (3) metabotypes from unsupervised clustering within EFV-treated LTWC. Abbreviations: LTWC: long-term well-controlled CLWH, MT-A: metabotype A, MT-B: metabotype B.

At the outset of investigation into metabolic factors in CLWH, potential confounders were minimized by selecting participants based on sexual development (Tanner stage) and, for CLWH, viral suppression (Figure 1). Initially, UCs were compared to CLWH overall as a starting point. Given reported effects of ART regimen and sex on metabolism, these factors were preliminarily explored before formal stratification to more rigorously define observed differences.

Clinical and demographic characteristics of UCs (*n* = 155), CLWH (*n* = 155), and CLWH subgroups [stratified by ART regimen; efavirenz (EFV, *n* = 101) or lopinavir/ritonavir (LPV, *n* = 54)] are in Table 1; ART regimen details are in Table S3. The original cohort had age-matched UC and CLWH participants ± six months, but in this sub-set CLWH were slightly younger than UCs (median 7.85 versus 8.18 years, *p* = 0.034). However, when stratified by ART regimen, age differences were no longer significant (EFV-CLWH = 8.11, versus UC: *p* = 0.061; LPV-CLWH = 8.19 years, versus UC: *p* = 0.254). As all participants were prepubertal, these minor differences are unlikely to be developmentally meaningful.

**Table 1:** Clinical characteristics of the pre-pubescent, virally suppressed metabolomics subset of CHANGES, stratified by HIV status and ART regimen, relative to uninfected controls

	UC^a^	CLWH^a^	p^b^	EFV-CLWH^a^	LPV-CLWH^a^	p^b^ (EFV vs. LPV)	p^b^ (UC vs. EFV)	p^b^ (UC vs. LPV)
No of participants	155	155		101	54			
Age (years)	8.58 ± 1.35 (8.18; 1.89)	8.14 ± 1.09 (7.85; 1.21)	**0.0340**	8.11 ± 1.04 (7.92; 1.17)	8.19 ± 1.18 (7.71; 1.34)	>0.9999	0.0608	0.2543
Sex (male: female)	76:79 (49.03%:50.97%)	90:65 (58.06%:41.94%)	0.3834	47:54 (46.53%: 53.47%)	29:25 (53.70%: 46.30%)	0.1951	0.1951	0.1951
BMI	16.60 ± 3.10 (15.95; 3.06)	15.34 ± 1.51 (15.13; 1.92)	**0.0001**	15.32 ± 1.49 (15.12; 2.20)	15.37 ± 1.56 (15.17; 1.49)	>0.9999	**>0.0024**	**>0.0164**
Height (cm)	126.80 ± 7.80 (127.00; 12.40)	121.60 ± 7.23 (120.50; 9.70)	**<0.0001**	121.40 ± 6.95 (119.80; 9.30)	122.00 ± 7.78 (121.80; 13.20)	>0.9999	**<0.0001**	**0.0005**
Weight (kg)	26.98 ± 7.17 (25.30; 7.40)	22.82 ± 3.94 (22.60; 4.60)	**<0.0001**	22.71 ± 3.68 (22.6; 4.8)	23.04 ± 4.43 (22.7; 4.6)	>0.9999	**<0.0001**	**<0.0001**
CD4 count (cells/ml)		1069.00 ± 350.10 (1038.00; 411.00)		1060.00 ± 306.10 (1020.00; 349.00)	1086.00 ± 423.00 (1056.00; 573.70)	0.8201		
CD4%		37.69 ± 6.28 (37.63; 8.65)		37.94 ± 6.35 (37.63; 8.55)	37.21 ± 6.18 (37.92; 9.46)	0.3946		
Viral load (HIV RNA copies/ml)								
Undetectable (VL < 50)		127 (81.94%)		83 (82.18%)	44 (81.48%)	>0.9999		
Detectable (50<VL < 1000)		28 (18.06%)		18 (17.82%)	10 (18.52%)	>0.9999		
Age at ART initiation (months)		8.52 ± 6.82 (5.53; 8.32)		8.335 ± 6.92 (5.10; 9.48)	8.88 ± 6.68 (6.87; 7.74)	0.3832		
ART duration		7.43 ± 1.04 (7.17; 1.12)		7.42 ± 0.98 (7.14; 1.16)	7.45 ± 1.14 (7.20; 1.24)	0.8758		
ART regimen switching history								
Ever on EFV: never on EFV					18:36 (33.33%:66.67%)			
Time since switch to EFV (years)				3.16 ± 1.45 (3.63; 2.12)				
History of long-term viral suppression (no blips >1,000)		91 (58.71%)		67 (63.37%)	24 (44.44%)	**0.0104**		

Abbreviations: UC: uninfected controls; CLWH: children living with perinatally acquired HIV; EFV: efavirenz-based antiretroviral therapy regimen; LPV: lopinavir/ritonavir-based antiretroviral therapy regimen; VL: viral load; ART: antiretroviral therapy.

a Continuous variables are given as median ±standard deviation (average; interquartile range) and categorical variables are given as n_x_:n_y_ (percentage_x_:percentage_y_).

b The *p* values were obtained from one of the following tests, as appropriate: Fisher’s exact test, Chi-square test, Wilcoxon rank sum, or Kruskal-Wallis with Dunn’s test as post-hoc analysis. Significant *p*-values are indicated in bold.

BMI, height, and weight were significantly lower in CLWH (including both EFV- and LPV-treated groups) than in UCs, consistent with prior findings in children with perinatally acquired HIV.^17^^,^^18^ The larger cohort has shown increased stunting and delayed pubertal development among CLWH.^19^ ART was initiated at a median of 5.53 months of age, with a median duration of 7.17 years at sampling. CLWH who switched to an EFV-based regimen had been on that regimen for a median of 3.63 years.

CLWH had been enrolled in a trial that randomized those suppressed on LPV at ages 3–5 years to continue treatment or switch to an EFV-based regimen, to evaluate a switch from PI- to NNRTI-based therapy.^20^ Participants were divided into those receiving EFV- vs. LPV-based ART. Most CLWH (95%) received two nucleoside reverse transcriptase inhibitors [NRTIs; abacavir (ABC) and lamivudine (3 TC)] with either one NNRTI (EFV) or one PI (LPV/r). Five used an alternative NRTI to ABC, together with 3 TC and EFV or LPV/r, and one child received both EFV and LPV/r but was grouped with the LPV/r group, as PI-based regimens are typically associated with greater metabolic alteration (especially relating to lipids and glucose metabolism) than other regimens.^21^^,^^22^ Results did not differ whether this child was included or excluded, nor were any of the children using an alternative NRTI flagged as outliers in any analysis (sensitivity analysis can be found in Tables S4 and S5). No adherence data were available for this cohort, although the untargeted metabolomics analysis method did detect all relevant ARVs (discussed in more detail later). Based on these data, 3 of the 155 CLWH were potentially non-adherent, as no ARVs were detected in these samples. All these potentially non-adherent children were of the LPV-CLWH group. However, as there was no record of when the last dose was taken and if it was taken, these measures could not be reliably compared head-to-head. As there is great variation in the pharmacokinetics and dynamics between individuals, even if a dose is taken at the same time, individual ARV levels may differ at a certain point in time. It is also possible that these data do not accurately reflect the real quantity of ARVs in plasma, as the data were batch corrected, especially as these 3 children did not stand out as outliers. All selected CLWH had viral load (VL) < 1,000 at sampling; most (75.48%) were undetectable (<20 HIV RNA copies/ml). No metabolic differences were found between detectable and undetectable VL groups, even within treatment groups. ART regimen remained the strongest grouping factor (Figure S5). The median CD4% of the CLWH was 37.63%, within the normal range for this age group,^23^ though some (*n* = 20, 12.90% of all included CLWH) had values below the 10th percentile for the age group (31%, median = 37%^23^). Similarly, the median CD4 T cell count was 1,069 cells/ml, within the normal range,^23^ with 12.98% (*n* = 20, 12.90%) falling below the 10th percentile (700 cells/ml). Only three children had both CD4% and CD4 T cell counts below the 10th percentile. None of the clinical characteristics were significantly different between the EFV-CLWH and the LPV-CLWH, except that the EFV-CLWH more often had sustained viral suppression (no recorded blips >1,000 HIV RNA copies/ml, *p* = 0.010). Given that it is yet unknown for how long metabolic changes induced by a particular ART regimen may persist, the clinical characteristics and metabolic profiles of the subgroups of LPV-CLWH who had either ever or never been on EFV were compared (Data S6). There were no significant differences in the distribution of sex or the immunological status at this time point (CD4 T cell count and percentage). There were also no metabolic differences when metabolic profiles were compared using Kruskal-Wallis with Dunn (KWD) (false discovery rate (FDR)<0.05). Three metabolites had raw *p* < 0.05 and 0.5< fold change (FC) < 2 (homocitrulline, 1,6-anhydroglucose, and N-acetylkynurenine). This suggests, along with other metabolites with significant FC from this comparison (Tables S6 and S17), that whatever degree of metabolic difference existed between those who had ever or never used EFV originated from the diet and/or the gut microbiota. Dietary or gut microbiota data were not available to further explore these hypotheses.

### Identification of major metabolic patterns in CLWH: Predominant effects of ART regimen and sex

**Figure 2:**
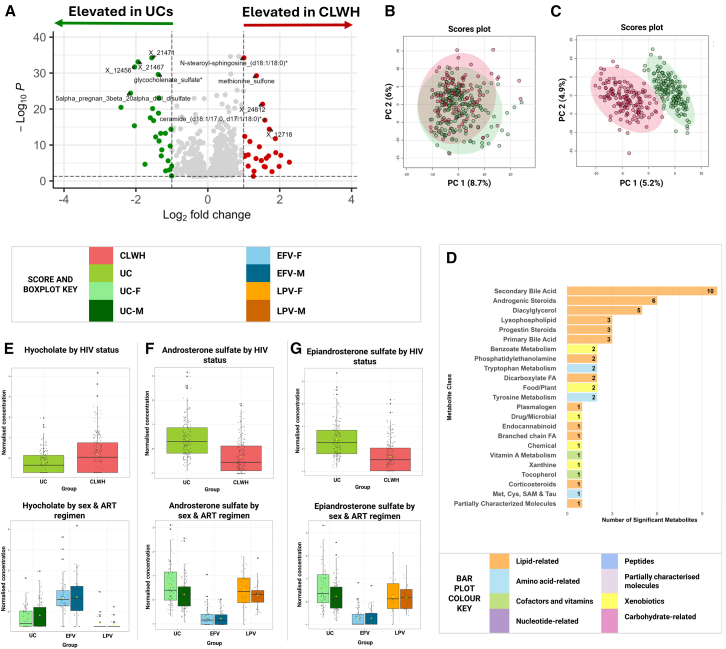
Identification of the predominant patterns in the metabolic profiles of CLWH: comparison of UCs to CLWH (A) A volcano plot highlights significantly altered metabolites (KWD, FDR <0.05; 0.5 < FC > 2, in color), with only the annotated metabolites out of the top five up- and top five downregulated metabolites labeled. (B) PCA scores plot. (C) PLS-DA scores plot shows clear group separation and strong model validation. (D) Bar plot illustrates metabolite class distribution among annotated metabolites significant in all but one test (WR FDR, ES, KWD, and PLS-DA VIP). These plots serve as visual aids only. Final lists of significant metabolites used for interpretation can be found in the supplementary material. Bar colors denote metabolite super-pathways. (E–G) Paired boxplots of three highly significant metabolites, showing ART regimen- and sex-specific differences. Yellow triangles in boxplots indicate the average. These trends differ between CLWH and UCs, underscoring the influence of treatment and sex on metabolic profiles. Abbreviations: FA: fatty acids, SAM: S-adenosyl methionine, and Tau: taurine.

To investigate metabolic alterations linked to HIV and/or ART, metabolic profiles of CLWH were compared to UCs, and demographic and clinical factors correlated with a meaningful and robust metabolic divergence were determined. Principal component analysis (PCA) (Figure 2B) indicated substantial overlap between groups, reflecting blood homeostasis. However, distinct differences emerged when additional principal components were considered (Figure S3), and well-validated partial least squares-discriminant analysis (PLS-DA) showed near-complete group separation (Figure 2C, a three-component model: accuracy = 0.97, R2 = 0.87, Q2 = 0.80, permutation testing *p* value = 0.0005). This indicated that although representing a small proportion of the metabolome, there were distinct differences between UCs and CLWH.

Metabolites were selected for further analysis of CLWH and UCs if they met the following criteria: Wilcoxon rank-sum test (WR) FDR < 0.05 with r > 0.5, 0.5< FC > 2, and PLS-DA variable influence on projection (VIP) > 1.5. This yielded 33 metabolites meeting all criteria [54.55% (18 metabolites) being lipids and 27% (9 metabolites) unannotated metabolites, see Table S24 for details] and another 38 meeting all but one (with lipids and unannotated metabolites dominating both these sets of metabolites, see Table S24 for details). Of the 71 metabolites, 53.55% (38 metabolites) were lipid-related, 25.35% unannotated (18 metabolites), and the remainder included amino acids, cofactors and vitamins, xenobiotics, and partially characterized molecules (Table S24). Bile acids (BAs, primary and secondary), androgenic steroids, and diacylglycerol sub-pathways were the most represented within the group of significantly different metabolites from the lipid super-pathway (Figure 2D).

Table S24. Summary of all statistics applied for exploratory analyses (UC vs. CLWH)

Boxplots of these metabolites stratified by ART regimen and sex revealed that ART had a stronger effect than sex on metabolite levels, though stratification reduced sample size. Often, significant differences between UCs and CLWH were driven by a single ART group, while others resembled UCs, showed weaker trends, or opposite effects. Examples of these patterns amongst the metabolites that were most significant between UCs and CLWH based on all applied criteria are shown in Figures 2E–2G.

Based on these findings, three structured analyses were conducted: (1) comparing ART regimens; (2) assessing sex effects in UCs and how ART influences sex-related metabolism in CLWH; and (3) identifying metabotypes within CLWH on EFV. Associations between clinical factors and metabolites were examined for each comparison.

### Bile acids, bilirubin, and androgenic steroids primarily distinguish EFV-CLWH from LPV-CLWH (comparison 1)

While both treatment groups shared several metabolic differences from UCs, they also exhibited distinct profiles. Shared differences fell into three categories: qualitative (same metabolite, different trends), quantitative (similar extent), or trending (same trend, different extent) (Figure S10 and Table S25).

Table S25. Summary of all statistics applied for comparison 1 (UC vs. CLWH-EFV vs. CLWH-LPV)

**Figure 3:**
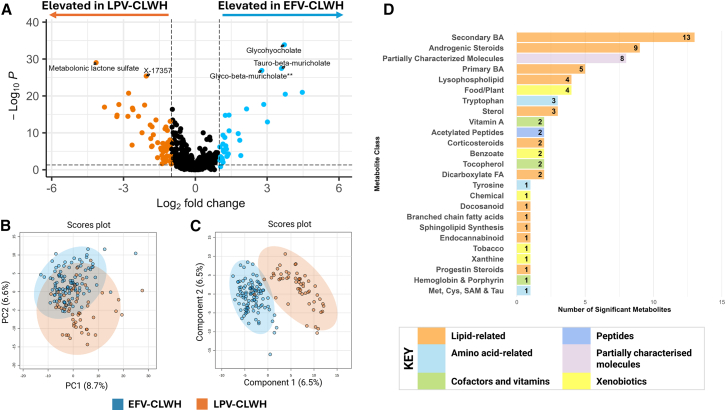
Comparison of CLWH on an EFV- and LPV-based ART regimen The volcano plot (A) highlights significantly altered metabolites (KWD, FDR <0.05; 0.5 < FC > 2, in color), with the top five—mostly secondary BAs—labeled. PCA (B) and PLS-DA (C) plots illustrate group separation; PCA revealed partial overlap, and the PLS-DA validated well. A bar plot (D) shows class distribution of annotated metabolites significant in KWD and at least two other tests (ES, FC, PLS-DA), with secondary BAs most frequently affected by ART. Bar colors denote metabolite super-pathways. Abbreviations: BA: bile acids, FA: fatty acids, SAM: S-adenosylmethionine, and Tau: taurine.

Univariate analysis (FC and KWD) identified three BAs, metabolonic lactone sulfate, and an unannotated metabolite as the top five discriminatory features (Figure 3A). PCA showed partial separation (Figure 3B), whereas the PLS-DA yielded clear group separation and strong validation (Figure 3C, a five-component model: accuracy = 0.94, R2 = 0.96, Q2 = 0.71, permutation testing *p* value = 0.01). Of 99 metabolites achieving statistical significance in three or more tests (Table S25), 27 were unannotated, leaving 72 interpretable metabolites. Lipid-related metabolites dominated (43/72), followed by xenobiotics (9/72; mainly benzoate and food metabolism) and partially characterized molecules (8/72, including 5 bilirubin metabolites) were the next most frequent super-pathways differentiating the treatment groups (Figure 3D). The lipid metabolites were primarily primary/secondary BAs, androgenic steroids, lysophospholipids, sterols, dicarboxylate fatty acids, corticosteroids, and a progestin steroid, amongst others (Table S25). Microbial tryptophan (Trp) metabolism accounted for three of five amino acid-related metabolites, while vitamin A and tocopherols represented the vitamin/cofactor pathway metabolites.

Both the CLWH groups, to varying extents, exhibited decreased levels of the primary bile acid cholate, conjugated chenodeoxycholate (glycochenodeoxycholate 3-sulfate), and secondary bile acid conjugates (deoxycholic acid glucuronide and glycoursodeoxycholic acid sulfate), while taurochenodeoxycholate was increased. The LPV-CLWH had higher levels of conjugated primary BAs (taurocholate, glycochenodeoxycholate glucuronide, and glycocholate) and lower levels of the unusual primary BAs tauro-beta-muricholate and glyco-beta-muricholate (the latter not Tier 1-identified) compared to the EFV-CLWH. Of the secondary BAs, most deoxycholate derivatives were elevated in LPV-CLWH versus both EFV-CLWH and UCs. Hyocholate derivatives were increased in EFV-CLWH compared to LPV-CLWH and UCs but were significantly lower in LPV-CLWH than UCs. Lithocholate derivatives were decreased in EFV-CLWH relative to UCs, while LPV-CLWH resembled UCs (Figure S10 and Table S25).

Unconjugated bilirubin (several isomers), biliverdin, urobilin, and five partially characterized bilirubin breakdown products were significantly decreased in EFV-CLWH versus LPV-CLWH and UCs, but significantly higher in LPVs than UCs. Heme levels did not differ. The microbiota-derived catabolite of bilirubin, urobilinogen, was excluded from the dataset by the 50% zero filter (detected in less than 20% of samples in both the CLWH and UC groups). However, some CLWH showed extreme urobilinogen values.

Various sulphated versions (-S) of androgenic steroids in the EFV-CLWH and LPV-CLWH were significantly different and differed from UCs. These included sulfates of androsterone, androstenediol (also different isomers), epiandrosterone, dehydroepiandrosterone (DHEA), and the 16-alpha hydroxylated form of DHEA. EFV-CLWH versus LPV-CLWH showed the greatest divergence from UCs, with reduced levels of most sulphated androgenic steroids, but elevated 16-alpha-hydroxy DHEA 3-sulfate and a metabolite annotated as “andro steroid monosulfate C19H28O6S (1)” relative to LPV-CLWH and to UCs. The latter metabolite, not identified at Tier 1 level, is listed in Metabolomics Workbench (ME405852) as DHEA-S and linked to the same KEGG ID, but showed an opposite trend to the DHEA-S identified here, while it paralleled 16-alpha-hydroxy DHEA 3-sulfate. In contrast, LPV-CLWH showed general steroid upregulation, notably differing from UCs in having elevated DHEA-S, androstenediol (3beta,17beta) disulfate (1), androstenediol (3beta,17beta) monosulfate (1), and decreased 5-alpha-androstan-3beta,17beta-diol disulfate. Corticosteroid metabolites, including cortisol, were elevated in LPV-CLWH versus other groups [cortolone glucuronide (1), cortisol, tetrahydrocortisone glucuronide (5)] and decreased in EFV-CLWH relative to UCs, except for tetrahydrocortisone glucuronide (5), which was increased.

Multivariate receiver operating characteristic (ROC) analysis (Figure S8) identified a five-metabolite model that best distinguished EFV-CLWH from LPV-CLWH (AUC = 0.972, CI: 0.932–0.998), comprising metabolonic lactone sulfate (top univariate performer, Figure S9), hydroxy-CMPF (not-Tier 1 identified), 3beta-hydroxy-5-cholenoate, and two unannotated metabolites (X-16935, X-17357). Based on Spearman-rank correlations between the two unannotated metabolites and all other metabolites, it is likely that these metabolites are also related to steroid, bile acid, or fatty acid metabolites (details in Data S7). Both these metabolites correlated most strongly with metabolomic lactone sulfate, and showed a similar pattern of being significantly lower in the EFV-CLWH than LPV-CLWH and UCs (the latter two groups showing similar levels, Figure S20). No additional information on these metabolites’ identities was available from Metabolon at the time of publication.

### Metabolic sexual dimorphism differs by HIV status and ART regimen (comparison 2)

**Figure 4:**
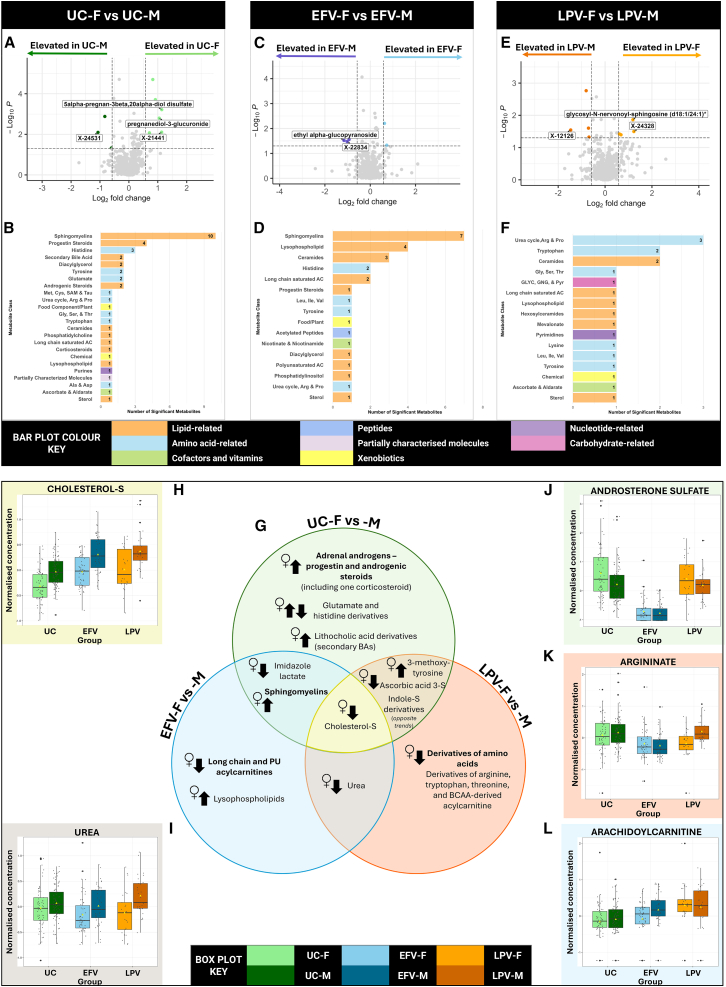
Metabolic sexual dimorphism differs by HIV status and ART regimen The volcano plot (A, C, and E) highlights significantly altered metabolites (KWD, FDR <0.05; 0.6<FC > 1.5, in color), with only those with KWD, FDR <0.05 and 0.5<FC > 2, labeled. Bar plots (B, D, and F) highlight class distributions among annotated metabolites significant or near significant by KWD (FDR <0.05 or <0.06). The metabolites approaching significance were included in B, D, F, and G–L to provide wider biological context but should be considered exploratory (see Table S26 for the statistical metrics of each metabolite). Increased lipid-related metabolites most frequently distinguished female from male children. While some overlap existed across groups, sex-associated metabolites largely differed between UCs and CLWH, and between ART regimens. A Venn diagram (E) shows the metabolic patterns between the sexes in each group and between groups, with representative examples as boxplots (H–J). Yellow triangles in boxplots indicate the average. Bold text indicates a pattern in a specific class of metabolites, whereas normal text indicates individual but notable, differential metabolites. The arrows represent trends in females relative to males. This visual is not exhaustive; it only represents major patterns in the data, and several other individual metabolites were also differentially detected in the various groups. Abbreviations: S: sulfate, PU: polyunsaturated, BA: bile acids, BCAA: branched-chain amino acids. Abbreviations: FA: fatty acids, SAM: S-adenosyl methionine, Tau: taurine, AC: acylcarnitine, GLYC: glycolysis, GNG: gluconeogenesis, and Pyr: pyruvate.

PCA score plots (Figures 4A–4C, and 4E) indicate that sex differences at the prepubescent stage represent a small portion of the overall variation of metabolome. Cholesterol-S was the only metabolite consistently significantly higher in females across all groups (KWD FDR<0.05, Figure 4G and Table S25). Thus, the metabolic expression of sex varied among UCs, EFV-CLWH, and LPV-CLWH (Figures 4B–4D, 4F, and 4H–4J). In EFV- and LPV-CLWH groups, but not UCs, urea and one unannotated metabolite were significantly lower in females (Figure 4G). As these comparisons yielded fewer significant metabolites, metabolites approaching significance (KWD FDR<0.06 and 0.6<FC < 1.5, 9 metabolites) were used to better understand and interpret the underlying biochemistry of this sexual dimorphism (and are included in the bar plots and Venn diagram), although these were not considered markers of sexual dimorphism. The supplementary information may be consulted for the statistical performance of each metabolite (Table S25).

Although cholesterol (as measured conventionally and by metabolomics) and cholesterol sulfate were increased in CLWH compared to UCs, no significant differences were found between the EFV-CLWH and LPV-CLWH. As expected, females had higher whole-body total fat percentage than males - this was the only conventional fat/cholesterol metric showing sexual dimorphism.

In UCs, lipid metabolites primarily distinguished the sexes (Figure 4B), notably steroids and sphingomyelins. Four metabolites were significantly elevated in female UCs across all statistical methods (KWD, effect sizes [ES], and FC; the complete list of significantly different metabolites can be found in Table S25). These included three conjugated isomers of the main metabolite of progesterone, pregnanediol-3 (5α-pregnan-3β-20α-diol monosulfate and disulfide, and 5α-pregnan-3β-20β-diol monosulfate),^24^ and androsterone sulfate. These sex-based trends were attenuated in CLWH and no longer significant. In EFV-CLWH, sex differences involved lipid-related metabolites (Figure 4D), while in LPV-CLWH, amino acid-related metabolites dominated (Figure 4F).

Only 6 of 72 sexually dimorphic metabolites (in any group, KWD only) overlapped with the 99 treatment-discriminating metabolites (significant in three or more metrics), indicating the most sexually dimorphic metabolites were not those most affected by treatment.

### Unsupervised clustering identifies two main CD4%-associated metabotypes among LTWC-CLWH, distinct from UCs (comparison 3)

Due to the strong impact of ART on metabolic profiles, associations between clinical parameters used in HIV cure studies and metabotypes identified by unsupervised analyses were assessed within a historically well-suppressed subset [undetectable viral load with occasional blipping, for >6 years, with no blips >1,000 HIV RNA copies/ml] of the larger of the two treatment groups of CLWH (EFV), to maximize statistical power. All these EFV-LTWC children were on the same ART regimen (EFV, 3 TC, and ABC). A subset of age and sex-matched UCs was used to establish a baseline. Due to reduced sample size, sexual dimorphism was not assessed.

**Figure 5:**
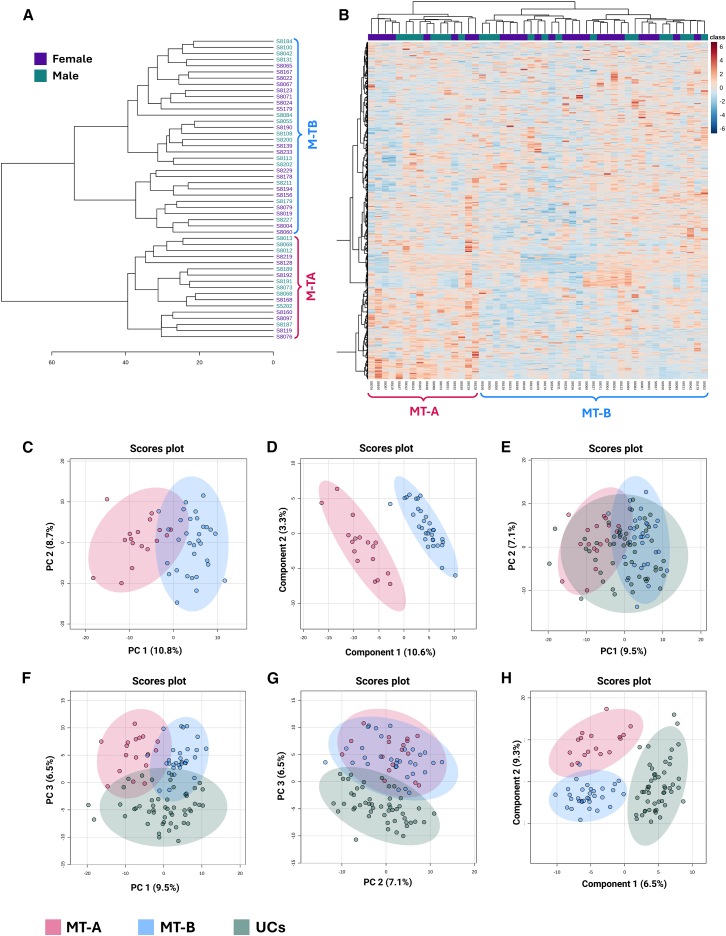
Metabotype identification and comparison in the EFV-LTWC The HCA dendrogram (A) separated MT-A (pink) and MT-B (blue), with sex distribution shown in both the dendrogram and heatmap (B; female: purple, male: green). Although overall metabolomes are similar, distinct metabolites (bottom of heatmap) differentiate the metabotypes. PCA (C) shows near-complete separation of metabotypes, supported by borderline-validated PLS-DA (D). PCA plots comparing UCs to MT-A and MT-B indicate shared metabolic features (E and G), but also that metabotypes divergence from UCs in different ways (F and H).

Hierarchical clustering analysis (HCA) identified two clusters in the EFV-LTWC: metabotype A (MT-A) and metabotype B (MT-B, Figure 5A; distribution of the sexes within the clusters also shown). The K-means algorithm produced the same groups, except for one participant who grouped differently (Table S8), though their clinical profile did not differ notably. HCA clusters were thus retained for subsequent analyses (referred to as MT-A and MT-B). Plasma levels of EFV, 3 TC, and ABC (detected by metabolomics) did not differ significantly between MT-A and MT-B. Although a similar group of LTWC children on LPV was analyzed in the same manner, no clear and consistent clusters were found. The clusters identified by HCA in the LPV-LTWC did not show statistically significant metabolic differences (KWD FDR) (Figure S19 and Table S19), and therefore, this analysis was confined to the EFV-LTWC group.

Among the clinical metadata [Table S27], age at ART initiation, pre-ART CD4 percentage and VL, time to suppression (400 HIV/RNA copies/ml), longitudinal viral load, or HIV DNA as a proxy for reservoir size—did not differ between MT-A and MT-B groups. MT-A and MT-B differed significantly only in contemporaneous (*p* = 0.0007) and longitudinal CD4% (*p* = 0.008); CD4 T cell count approached significance (*p* = 0.06). Longitudinally, MT-A more frequently had CD4% below 30%, while MT-B more frequently never had a CD4% below 30%, after first reaching undetectable VL (there was a significant difference in the frequency of CD4% dipping below 30% between groups, *p* = 0.03), but there are exceptions to the pattern in both groups. The clusters remained consistent even when those with CD4% lower than the 10th percentile for their age were removed, suggesting that the relation between CD4% and metabolites exists even within the healthy range. This implies that the functional capacity, or fitness, of the immune system may be impaired more severely in MT-A than MT-B. While CD4/CD8 ratios were not available, a lower CD4% implies a higher proportion of other lymphocytes, commonly CD8 T cells. This would suggest that the lymphocyte subset distribution in MT-B may be more severely disrupted in MT-A relative to MT-B. CD4% at baseline has been shown to be the strongest predictor of longer time to rebound in infants with early, short-term ART treatment (discussed by Tiemessen^25^) and correlated with viral reservoir size.^26^^,^^27^ CD4% was also part of a set of criteria (in combination with normal CD4 count, rather than in preference to it) to determine infants’ eligibility to enter an analytical treatment interruption in the IMPAACT P115 trial.^28^ Together, this indicates that CD4% is not considered simply as a monitoring parameter but may also have prognostic value in the long-term. In the context of testing new treatments or interventions, our results suggest that there may be a group of CLWH resembling MT-A in a trial who might not respond to the intervention. This subgroup would not be predictable from the clinical parameters alone but may be identified by metabolic parameters. Whether these metabolic alterations correspond to more refined measures of immunological function remains to be elucidated.

Table S27. Metadata for the EFV-LTWC subset of CLWH

### MT-A shows more impaired mitochondrial oxidative metabolism than MT-B (comparison 3 continued)

Although overall metabolomes were similar, as might be expected within a consistently virally suppressed group on the same ART regimen, distinct metabolites separated MT-A from MT-B (Figure 5B). PCA showed near-complete separation (Figure 5C), and PLS-DA had strong cross-validation metrics (Figure 5D) (a four-component model: accuracy = 0.92, R2 = 0.99, Q2 = 0.76) but failed in permutation testing (*p* = 1), likely due to limited sample size. In the PCA scores plots including the UCs (Figures 5E–5H), the two groups of CLWH share differences from the UCs (480/1220 metabolites), though with different trends and to varying degrees in some cases. Each group also had unique differences from UCs. A total of 260 metabolites significantly differed between metabotypes via KWD (FDR<0.05); 68 also showed significant fold change (0.5<FC > 2) and PLS-DA VIP (>1.5) (Table S28). Early ART in CLWH is associated with better immune recovery and a decreased viral reservoir size, and as such, these factors had the potential to have contributed to the metabotype divergence. Although there were no significant differences between the age at ART initiation and viral reservoir size between the metabotypes, the effects of these variables were further assessed using logistic regression, permutational multivariate analysis of variance (PERMANOVA), and linear models with and without covariate corrections. ART initiation and viral reservoir did not contribute to the metabolic divergence of the metabotypes, nor could these variables predict the metabotype grouping. However, CD4% could predict metabotype based on logistic regression, and explained a small proportion of variance in the PERMANOVA, albeit non-significantly (2.4% versus 6.2% explained by metabotype) (Figure S13 and Tables S11–S15).

Table S28. Summary of all statistics applied for comparison 3 (UC vs. MTA vs. MTB)

Most altered between MT-A and MT-B were increased levels of two partially characterized glutamine conjugates: C7H12O2∗ (FDR = 1.39 × 10^−8^, FC = 14.22, VIP = 3.66) and C6H10O2 (2)∗ (FDR = 1.50 × 10^−5^, FC = 8.45, VIP = 3.18); palmitoleate (16:1n7) (FDR = 3.16 × 10^−8^, FC = 4.85, VIP = 3.25); the ketone body, 3-hydroxybutyric acid (3-HBA; FDR = 1.98 × 10^−8^, FC = 8.52, VIP = 3.60) and its acylcarnitine form [(R)-3-hydroxybutyrylcarnitine, FDR = 1.25 × 10^−8^, FC = 4.86, VIP = 3.11]. Two other detoxification derivatives of 3-HBA, 3-hydroxybutyroylglycine∗∗ (an acyl glycine) and the S-stereoisomer of the acylcarnitine, were also significantly elevated in MT-A, though to a lesser extent. The glutamine conjugates diverged in trend: elevated in MT-A vs. UCs, but reduced in MT-B.

MT-A also showed significant alterations (based on at least one statistical test) in fatty acids (medium/long-chain saturated, polyunsaturated, monohydroxy, dicarboxylic), endocannabinoids, and acylcarnitines. Essential amino acids (including Trp and its immunomodulatory metabolites), lactoyl amino acids, and dopamine 4-sulfate were lower in MT-A, whereas phosphate, nucleotide metabolites, bilirubin, and its related metabolites were higher in MT-A. Many metabolites linked to mitochondrial damage correlated strongly with CD4%, as did glutamine (which generally associates with immune functions^29^ and with HIV^30^^,^^31^^,^^32^) and bilirubin. Previously, bilirubin and its by-products were significantly lower in EFV-CLWH than in LPV-CLWH and UCs. Within EFV-LTWC, this pattern held only in MT-B; MT-A had bilirubin levels similar to UCs, indicating divergent responses to the same treatment regimen.

### MT-B has significantly lower bilirubin than MT-A (comparison 3 continued)

MT-B appeared less of an extreme phenotype than MT-A (388 vs. 411 significantly different metabolites compared to UCs; KWD FDR, less severe deviation from UCs in most metabolites). A hallmark of MT-B was significantly lower levels of 12/16 bilirubin-related metabolites (including the predominant ZZ-isomer of bilirubin, i.e., bilirubin itself) compared to both MT-A and UCs. Both MTs showed similarly reduced levels of the configurational bilirubin isomer bilirubin^33^ (E,Z or Z,E − non-Tier 1) versus UCs. Trp was lower in both, but lowest in MT-B. MT-B also had low levels of kynurenine (Kyn) and kynurenate, while MT-A had similar levels of this immunomodulatory catabolite of Trp to UCs, paralleling findings in elite controllers with distinct Trp metabolism.^34^

### Unannotated metabolites and phospholipids consistently associate with HIV-related alterations, independent of ART regimen

**Figure 6:**
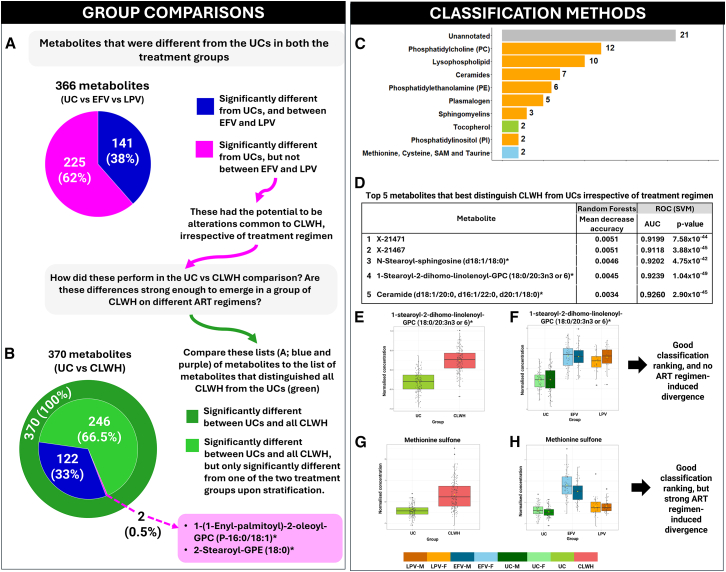
Unannotated metabolites and phospholipids consistently associated with HIV-related alterations, independent of ART regimen Of 225 metabolites deviating similarly in EFV- and LPV-CLWH versus UCs (A), only two—both unidentified phospholipids—differed significantly from UCs in unstratified CLWH (B). Classification models distinguishing CLWH from UCs also highlighted phospholipids and unannotated metabolites as key discriminators (C and D). However, ART regimen effects remained a confounding factor even among top-ranked metabolites (E–H). Yellow triangles in boxplots indicate the average. Asterisks (∗) at the end of metabolite names indicate features that were confidently identified by Metabolon, but which could not be confirmed using a reference standard.

Of the two approaches used to identify metabolites consistently altered in CLWH versus UCs regardless of ART regimen (details in Methods Section), the group comparisons method yielded only two markers, both of which were glycerophospholipids (Figures 6A and 6B). The classification method identified more potential markers, predominantly unannotated metabolites, and various classes of phospholipids (Figures 6C and 6D). While some high-ranking metabolites in both models (random forests [RF] and ROC) showed substantial ART-induced divergence—highlighting the ART regimen as a strong confounder—this does not preclude discovery of HIV-related metabolic markers through systematic analysis. The prominence of unannotated features among top classifiers suggests the involvement of yet-uncharacterized metabolites in HIV-associated metabolic changes and indicates current biological and technical limitations in biomarker identification. After curating ART effects, X-21471 and X-21467 emerged as the highest-ranking classifiers of HIV status regardless of ART regimen (Table S16). These metabolites are linked to polymorphisms in genes encoding mitochondrial transcription termination factor 1, dynamin binding protein (X-21471), and several members of the solute carrier organic anion transporter family,^35^^,^^36^ cardiovascular parameters^37^ (X-21467), and multidrug resistance-associated protein 2^35^^,^^36^ (which transports Ritonavir^38^ and is modulated by EFV,^39^ X-21471 and X-21467) (Table S12). These associations suggest that HIV, the immune response, or the ART regimen backbone (3 TC and ABC), may influence related biological pathways, though this requires further investigation. No correlation was found between these top-ranked metabolites and VL (even when those who were excluded from all other analyses due to viremia >1,000 copies/ml were included), nor with HIV DNA PCR Ct values (used as a proxy for HIV DNA reservoir size). Findings suggest these metabolic alterations are not directly related to viral replication or reservoir size.

## Discussion

This study investigated the metabolic consequences of perinatally acquired HIV during childhood to further clarify the specific clinical drivers of metabolic variation in this population. Understanding how clinical factors shape the metabolic profile is critical for optimizing the use of metabolomics in HIV cure research and clinical management. While previous studies have successfully linked markers like CD4 T cell counts and viral loads to specific metabolic changes, variations in cohort sizes and clinical heterogeneity across the literature have made it challenging to establish consistent patterns. To build upon these findings, our study utilizes a large, well-characterized cohort, which enabled statistically robust comparisons—particularly regarding ART regimen and sex—while controlling for age, sexual development, and viral load. Other outcomes from the CHANGES cohort have been previously published.^19^^,^^40^^,^^41^^,^^42^^,^^43^^,^^44^^,^^45^^,^^46^^,^^47^

Our findings highlight three key points. First, ART regimen profoundly affected the plasma metabolome in virally suppressed CLWH. Second, metabolic signatures of sex in prepubescent children were influenced by both HIV and ART regimen. Third, the emergence of distinct metabotypes among EFV-treated children with excellent viral control indicated that individual responses to ART may impact immunological parameters via immunometabolic signaling. These results, though done in the pediatric context, have implications for the interpretation of all previous work in the HIV metabolomics field, as the ART regimen and sex have either rarely been considered as confounders or effect modifiers, or the sample size was too small to accommodate the necessary corrections.

As in adult PLWH,^10^ systemic lipid metabolism was the most altered metabolic pathway in CLWH on ART. Although all CLWH shared metabolic characteristics that distinguished them from UCs, EFV- and LPV-CLWH also differed substantially from each other, with potentially profound physiological implications. Simple case-control comparisons that ignore ART regimen may thus obscure important treatment-specific effects. Given the minimal influence of viral activity in virally suppressed PLWH, ART regimen and chronic inflammation likely drive most metabolic changes. The pharmacological diversity of ARVs, and their use in different combinations, together with individual variability in drug metabolism, makes it improbable that meaningful metabolic biomarkers can be identified without adequate consideration of ARV effects in virally suppressed, treated PLWH. Therefore, the specific metabolic alterations of the two CLWH groups based on treatment regimen will be placed into biological context to highlight this point.

Differences between EFV- and LPV-CLWH were dominated by alterations in BAs and bilirubin catabolites. Both these groups of metabolites originate in the liver, interact with the intestinal microbiota, and can be returned to the liver. The liver is highly exposed to compounds originating from the intestinal lumen.^48^ As this cohort was primarily on 3 TC, ABC, and either EFV or LPV/r, differences may result from differential EFV and LPV interactions with human conjugation and cytochrome enzymes (e.g., CYP450s), transporters, and the gut microbiota. For example, CYP3A4, a major cytochrome in the liver and intestine responsible, amongst other things, for the detoxification of BAs before their conjugation,^49^ is strongly and irreversibly inhibited by ritonavir^50^ (and to a lesser extent by lopinavir^51^), and induced by EFV.^52^ Although no direct measures of liver function were available, these data suggest potential sub-clinical liver toxicity.

Primary BA production in the liver influences the BAs available to the gut microbiota for conversion to secondary BA. In turn, the composition and functional capacity of the microbiota influence which conversions can take place, as well as their extent.^53^ Also, the antimicrobial effects of both the primary and secondary BAs, as well as those of ARVs, may influence the gut microbial ecology.^54^^,^^55^^,^^56^ Different ARVs have varied gut-tissue penetrative capacities, antimicrobial effects, direct contributions to mucosal inflammation, and interactions with microbial enzymes.^57^^,^^58^ Each drug and combination of drugs may have different side effects, with other physiological changes, which may also result in gut microbial changes. The gut microbiota composition is affected differently by NNRTIs and PIs in adult PLWH^57^^,^^59^^,^^60^^,^^61^^,^^62^^,^^63^ and CLWH,^64^ but the conclusions regarding which regimen exerts the most detrimental effects are not consistent. Studies in PLWH that measure both microbial composition and metabolic profiles are not designed to assess the effects of different regimens and often do not report on the exact regimens at all.^65^^,^^66^ Thus, the exact interactions between metabolites, the gut microbiome, and ARTs in PLWH are not well understood.

The influence of the gut microbiome on human metabolism, the recycling of compounds via the enterohepatic circulation, along with the host’s regulatory mechanisms through nuclear receptors and other mechanisms, form a complex feedback system that influences the bile pool at any given time.^53^ This may, in turn, influence the immune system, given that these BAs and their derivatives are in close proximity to the immune cell community of the gut and may enter the circulation and interact with the circulating immune cells.^67^^,^^68^^,^^69^ Differences in the chemical composition of the BA pool can influence the uptake of sterols and, therefore gene regulation related to sterol metabolism.^53^ Thus, the effects of different ARVs on the human and gut microbial systems involved in BA pool dynamics could conceivably result in widespread physiological differences.

Similarly, bilirubin alterations could stem from EFV- or PI-induced changes in hepatic processing and transport, as well as the ART-induced gut microbial changes. Short-term EFV administered to healthy subjects appears to promote bilirubin elimination (reduced serum levels) via UGTA1A and MRP2 induction (this could also involve OATP and MRP3) through the constitutive androstane receptor and potentially the pregnane X receptor.^39^^,^^70^ Protease inhibitor-based treatments have been shown to induce hyperbilirubinemia (although more commonly associated with atazanavir and indinavir than LPV/r^71^^,^^72^), which accords with the higher levels of bilirubin and its catabolites in LPV-CLWH compared to UCs. The interconnected nature of human and gut microbial bile acid and bilirubin metabolism makes it difficult to speculate as to the exact origin of these observed changes, especially in the absence of gut microbial composition and metabolic measurements. Thus, more research is needed to determine whether these changes can be ascribed to the effects of ART on the human system or HIV and ART-induced microbial changes, or whether these changes are the result of the cumulative effects of these factors.

Metabolonic lactone sulfate, a partially characterized, steroid-like metabolite, was notably lower in EFV-CLWH compared to LPV-CLWH and to UCs. Though its exact identity is unknown, this steroid-like metabolite has been associated with total body fat, fat distribution, fat distribution by sex,^73^ androgenic steroid levels,^74^ BMI,^75^ liver adiposity,^76^ and other cardiometabolic parameters^77^^,^^78^^,^^79^ in adults. This metabolite, along with multiple other unannotated metabolites, has the potential to increase our understanding of the effect of HIV and ART on physiology, but the laborious nature of their identification currently precludes that. For example, the two unannotated metabolites forming part of the five-component ROC model distinguishing EFV- and LPV-CLWH most strongly correlated with metabolomic lactone sulfate and other steroids or fatty acids, were detected at low levels only in the EFV-CLWH group, and X-16935 has been reported to be lower in PLWH on an EFV-based regimen than in those on another regimen.^80^ As these metabolites have been detected in HIV-negative individuals as well,^36^^,^^81^ they are unlikely to be related to the EFV molecule itself. Multiple other steroid metabolites also varied by ART regimen and sex.

While pre-pubescent, school-aged children are not yet subject to the extreme dimorphic influence of sex hormones, there appear to be detectable differences in their responses to HIV and ART.^82^ Notably, how sex influenced the metabolome was different between UCs and CLWH, and between the treatment groups within the CLWH. Cholesterol-S, the circulating, conjugated version of the highly hydrophobic cholesterol molecule,^83^ was the only metabolite to consistently show sex differences (higher in males than females) across groups. This may reflect earlier adrenal activation and pubarche in females, given their higher CYP11A1 activity (rate-limiting for steroidogenesis),^38^ lower cholesterol sulfotransferase activity compared to prepubescent males, and elevated androsterone, estrogens, and androgens.^40^ Lower cholesterol-S in female children may imply greater cholesterol use in steroidogenesis, as desulfation makes it available for CYP11A1-mediated conversion to pregnenolone, the primary steroid precursor.

At approximately eight years of age, most of the CHANGES children were likely to have initiated adrenarche at the time point of sampling for metabolomics. Adrenarche, the maturation of the adrenal gland and the subsequent increased levels of the androgen precursors DHEA and its circulating store, DHEA-sulfate (DHEA-S) that precede pubarche,^84^ is another process with which ARVs can interfere, due to interactions with the enzymes of steroidogenesis (cytochromes and conjugation enzymes). The sexually dimorphic trends seen in the UCs regarding progestin and androgenic steroids were present but much less pronounced in CLWH and did not approach statistical significance, aligning with reported delayed puberty in CLWH.^17^ While pubarche was absent in all participants, CLWH were marginally younger than UCs; however, age differences were not present when stratified by ART regimen. SULT2A1-mediated conversion of DHEA to DHEA-S (DHEA must be unconjugated for conversion into other adrenal steroids^85^) may regulate adrenal androgen levels, as suggested by premature adrenarche cases.

The mechanisms and significance of mini-puberty and adrenarche—primarily human phenomena—remain poorly understood. Puberty occurs independently of adrenarche through the hypothalamic-pituitary-gonadal axis.^84^ One of the better-supported hypotheses is that adrenarche supports neurodevelopment, with neuroactive DHEA contributing to preadolescent brain remodeling. It may also enhance bone strength before puberty,^84^ which is particularly relevant for CLWH, who frequently have reduced bone mineral content,^17^ a pattern also noted in some CHANGES children.^19^ While the mechanism of bone density loss in CLWH may be complex, these findings suggest possible pathways. Adrenal steroids were lowest in EFV-CLWH, higher in LPV-CLWH, and highest in UCs. Shen et al.^19^ reported bone marker differences between EFV and LPV-CLWH, but significant bone marker-associated inflammatory marker variation.

Additionally, the development of iatrogenic Cushing’s syndrome in PLWH (including children) on LPV/r upon glucocorticoid administration,^86^^,^^87^ alongside elevated corticosteroids in the LPV-CLWH observed here, suggests subclinical hypercortisolism in this group of CLWH (via chronic inhibition of CYP3A4-mediated catabolism). Conversely, subclinical hypocortisolism could potentially result from EFV-induced inhibition of steroidogenesis via CYP21A2.^88^ Taken together with the roles of adrenal steroids on bone metabolism,^89^ this suggests that the augmentation of lower bone mineral content by different treatments may occur via distinct mechanisms. These effects may likely persist into adulthood, given the frequency of altered steroid metabolism in adult PLWH,^90^^,^^91^ with potential contribution to persistent immune dysfunction. Understanding these mechanisms could contribute to improved quality of life for CLWH, given steroids’ role in neurometabolism and the prevalence of neurocognitive complications in CLWH.

A potential mechanism contributing to the metabotypes observed in EFV-CLWH is the EFV-induced dose-dependent inhibition of complex I (CI) of the electron transport chain (ETC) *in vitro* at clinically relevant levels.^92^^,^^93^^,^^94^ The half-maximal inhibitory concentration of EFV-induced CI inhibition was <10 μM^94^—well below typical plasma levels (30–50 μM, sometimes higher^95^), with complete inhibition at 30 μM. CI inhibition impairs NADH oxidation to NAD^+^, elevating the NADH/NAD^+^ ratio (reported with EFV-induced CI inhibition^94^), which reduces proton pumping and ATP production via complex V (ATP synthase. This disrupts mitochondrial membrane potential, increases reactive oxygen species, and impairs NAD^+^ regeneration and calcium homeostasis. A sustained increase in the NADH/NAD^+^ ratio may hinder NAD^+^-dependent processes (e.g., β-oxidation) while promoting NAD^+^-regenerating pathways (e.g., lactate to pyruvate conversion, malate-aspartate and glycerophosphate shuttles, and ketogenesis). Prolonged inhibition of fatty acid oxidation may lower free coenzyme A (CoASH) levels, requiring reconjugation of CoASH-bound fatty acids, unable to enter oxidative pathways, to carnitine or glycine to regenerate CoASH and enable fatty acid clearance.^96^ EFV also induces lipid accumulation,^92^ oxidative and endoplasmic reticulum stress, and autophagy in endothelial cells in a dose-dependent manner.^97^ CI, the main source of reactive oxygen species^98^—especially during oxidative phosphorylation blockage—can cause localized damage (e.g., mitochondrial DNA mutations, iron-sulfur cluster oxidation) with potentially broader signaling consequences.

EFV-induced CI inhibition aligns with elevated ketone bodies, fatty acids, acylcarnitines, and acylglycines observed in MT-A, changes associated with impaired NAD^+^-dependent processes (e.g., β-oxidation) and upregulation of NAD^+^ and CoASH-regenerating pathways^6^^,^^7^ (Figure 6B). The more pronounced pattern in MT-A than MT-B is speculated to reflect higher EFV tissue levels due to polymorphisms affecting EFV metabolism,^95^ resulting in stronger inhibition (in the absence of genetic data, this hypothesis cannot be confirmed). Although plasma EFV levels did not differ between metabotypes (untargeted metabolomics assay), this may be confounded, as ART adherence and dose timing were unrecorded. Moreover, plasma and intracellular EFV levels often diverge, with substantial inter- and intraindividual variability.^95^^,^^99^ This variability is dependent not only on polymorphisms in the main EFV-metabolizing enzyme, CYP2B6, but also on various other cytochromes and conjugation enzymes.^100^ As EFV is ≥ 99% protein-bound in serum, tissue diffusion is limited, yet intracellular concentrations (e.g., in PBMCs) can be markedly elevated compared to other NNRTIs.^95^ Consequently, metabolic profiles of individuals on EFV may differ based on their complement of enzyme polymorphisms and may vary over time depending on dynamic physiological and pharmacological conditions.

Thus, EFV-induced CI inhibition may influence the immunometabolism of circulating immune cells. Altered NADH/NAD^+^ ratios can affect sirtuins (Sirt)—NAD^+^-dependent histone deacetylases involved in immune regulation and metabolic reprogramming, particularly in the mitochondria and endoplasmic reticulum, as demonstrated for EFV and Sirt3.^94^ Plasma sirtuin levels in PLWH on ART appear to vary by regimen (including integrase inhibitors) and may correlate with CD8 T cell counts.^101^ Further research is needed to clarify the role of redox balance and sirtuins in monitoring or modulating immune function in PLWH.

The influence of the ETC and metabolic pathways on immune function has only recently been recognized. ETC complexes and tricarboxylic acid cycle metabolites are now known to regulate T cell phenotypes, cytokine production, Treg activity, CD8^+^ exhaustion, Th17 responses, and B cell function.^98^ This, with the lower CD4% in MT-A compared to MT-B, and the performance of CD4% in regression models, suggests that CD4% is a contributor to the metabotype divergence, possibly due to different degrees of immunometabolic fitness in the two metabotypes. However, the direction of causality cannot be established in this study, and therefore, more research is needed to determine whether the metabolic consequences of drug toxicity drive lower CD4% or if lower CD4% (i.e., immunological history) drives the metabolic alterations, or if any other factors contribute to this observation. Such variability may partly explain the lack of consistent immune markers for clinical outcomes or viral control in PLWH. It is possible that early events, such as early treatment and consequently smaller viral reservoirs, may influence the functional immune recovery trajectory. Speculatively, the variance induced by such early events may be contained in the CD4% variation observed here at this later time point, although these factors (age at ART initiation and viral reservoir size) did not contribute to the metabolic divergence of the metabotypes in this study, and more research is needed to better understand these phenomena.

MT-B showed subtler differences from UCs than MT-A, complicating the contextualization of their unique metabolic attributes. MT-B may represent multiple metabotypes that could not be resolved due to limited statistical power (see HCA subgroups). However, lower bilirubin and related metabolites were consistent features of MT-B. Bilirubin metabolism appeared more inhibited in MT-B than MT-A, potentially due to EFV-induced enzyme and transporter inhibition^39^ and the individual genetic polymorphism profiles in MT-B. Hypobilirubinemia has been associated with metabolic disease^102^ and impaired cognitive function.^103^ While Trp was lower in both MTs compared to UCs, that of MT-B was the lowest. This, with concomitantly low levels of Kyn and kynurenate in MT-B (while MT-A had similar levels of these, resembling UCs), was reminiscent of a report of unique Trp metabolism in elite controllers.^34^ Typically, a high Kyn/Trp ratio is associated with ART-naive PLWH, which is typically partially restored upon ART. However, low levels of Trp and Kyn in elite controllers were associated with preserved Th17/Treg balance.^34^ Furthermore, altered immunomodulatory gut microbial Trp metabolites have been associated with immunological non-responders.^104^ MT-B showed better CD4% recovery (longitudinally and at the time of sampling) than MT-A, suggesting superior immunological fitness. Whether this reflects natural ability or reduced drug toxicity remains to be determined.

As ART plasma levels were not different between metabotypes, different degrees of ART adherence are unlikely to have driven the metabotype divergence. However, the ARV measurements may not reflect true drug exposure, as there was no control built into the original CHANGES study for the timing of sample collection since the last dose, nor was adherence actively monitored. Also, EFV metabolites, which may also be bioactive although not antiviral, were not assessed. Differences in intracellular or tissue drug levels - affected by transporter expression or detoxification gene variants - may contribute. For example, EFV has been reported to accumulate in cells, and plasma levels do not necessarily correlate with intracellular levels.^99^ At the time of the randomized ART regimen switching in the larger CHANGES study, CLWH with adverse reactions to EFV were switched back to the LPV-based regimens. Thus, the EFV-LTWC group excluded those with overt EFV side effects. These findings highlight that subclinical metabolic differences persist among CLWH on the same regimen who tolerate that regimen well and have maintained viral suppression for more than six years.

Recognizing distinct metabolic effects of ART regimens may improve biomarker curation for evaluating new therapies via ATIs and enhance understanding of post-treatment control mechanisms. The ART-related variation in key metabolites distinguishing CLWH from UCs in classification models shown here necessitates intentional biomarker curation. For example, after curation, various phospholipids predicted HIV status irrespective of the ART regimen, a metabolite class previously correlated with time to viral rebound and viral set point after treatment interruption in adult PLWH in a study where ART regimen could not be controlled for.^3^ We did not find any correlation between VL or reservoir size (HIV DNA PCR Ct) and highest-ranking phospholipids or unannotated metabolites from comparison 4 here, but this may be due to limited dynamic range in VL at this time point. Hypothetically, these metabolic changes may stem from immune responses or the ART backbone rather than viral replication. Additionally, individual phospholipids were either divergently affected by the ART regimen or not in this study; further work is needed to identify species linked specifically to immune or viral effects rather than treatment. These phospholipid alterations may also be linked to the early development of insulin resistance and cardiovascular disease (CVD) in CLWH, given their association with CVD risk in PLWH.^105^^,^^106^^,^^107^ Whether these alterations are related to continued immune activation and inflammation, or to the backbone ART drugs, is unclear. Although the associations of lamivudine with CVD risk have been contradictory,^108^ abacavir has been related to CVD risk more definitely.^109^ The relationship of these specific phospholipid alterations, the causative mechanisms, and CVD requires further investigation.

Although HIV- and ART-related metabolic changes are broadly described,^110^^,^^111^^,^^112^ underlying mechanisms remain unclear. Even when cell-specific pathways are known, their systemic impact is poorly understood. Moreover, links between metabolic shifts and clinical outcomes are inconsistent. These findings underscore the importance of accounting for the ART regimen in HIV-related studies of dynamic systems like the metabolome to understand persistent immune dysfunction despite suppressive ART. Metabolic differences across and within ART regimens may lead to varied responses to cure strategies, likely reflecting underlying gut and metabolic health, which in turn influence immune function. This suggests personalized ART adjustments and more robust participant characterization may be needed to identify those likely to succeed after treatment interruption in remission or cure studies. This would allow better interpretation of the effects of favorable genetics (such as human leukocyte antigen haplotypes), early treatment, immune modulators, and other interventions on the disease course and the curative potential of individuals.

EFV and LPV/r are older regimens, with EFV no longer used in pediatrics, but LPV/r is still recommended in some cases, and 3 TC and ABC remain in use with newer agents such as integrase strand transfer inhibitors (INSTIs). Although the specific metabolic mechanisms of different ARV combinations may differ, yielding different metabolic profiles, these findings are relevant for interpreting existing studies and understanding long-term complications among those previously exposed to older therapies. INSTIs, while less toxic, still cause metabolic disturbances such as weight gain, as well as more nuanced changes detected by metabolomics,^113^ the mechanisms of which are incompletely understood. Less toxicity does not necessarily equate to no metabolic effects, especially at the resolution that metabolomics provides.^114^ Newer agents like dolutegravir (part of the recommended pediatric and adult regimens) used in combination with tenofovir in adults, also affect mitochondrial^115^^,^^116^ and lipid metabolism.^117^^,^^118^^,^^119^ Specific ARV combinations can either attenuate or exacerbate each of the individual drugs’ effects.^120^ Our results reinforce the need to directly investigate ART regimen as a confounder or effect modifier in metabolomics research on PLWH and provide a framework in which this can be done.

### Limitations of the study

The biological and clinical implications of these findings require independent validation. Although several confounders were accounted for, unmeasured confounders include diet and pharmacogenetic background. Specifically, the CYP2B6 genotype (EFV metabolism) may contribute to the divergence of the metabotypes. While biological sex alone has the potential to cause differing metabolic profiles, behavioral and psychosocial sex differences likely contribute. Our population-specific data may not generalize to other geographical areas and socio-economic circumstances. Further metabolic research with other, more modern ART regimens is also needed to confirm these findings.

## Resource availability

### Lead contact

Further information and requests for resources and reagents should be directed to and will be fulfilled by the lead contact, Caroline Tiemessen (carolinet@nicd.ac.za).

### Materials availability

This study did not generate new, unique reagents.

### Data and code availability

• The metabolomics data and metadata used in this study have been deposited on the Biostudies repository (Biostudies: S-BSST2917, doi: https://doi.org/10.6019/S-BSST2917). This study did not generate any unique code.
• Any additional information required to reanalyze the data reported in this paper is available from the lead contact upon request.

## Acknowledgments

The study was supported in part by grants from the 10.13039/100000072National Institute of Dental and Craniofacial Research (grant no. DE028135) and the *Eunice Kennedy Shriver* 10.13039/100000071National Institute of Child Health and Human Development (grant nos. HD073952 and HD073977). C.T.T. is supported by the South African Research Chairs Initiative of the 10.13039/100016962Department of Science and Innovation, South Africa, and the 10.13039/501100001321National Research Foundation of South Africa (84177).

## Author contributions

Conceptualization, L.K., G.M.A., and C.T.T., methodology, C.H., L.K., and C.T.T.; formal analysis, C.H., T.W., S.W., F.L.; writing—original draft, C.H.; writing—review and editing, L.K., N.H.T., F.L., T.W., S.W., F.P., G.M.A., and C.T.T; funding acquisition, L.K., G.M.A., and C.T.T; supervision, L.K., and C.T.T, investigation, F.P. and R.S.

## Declaration of interests

The authors declare no competing interests.

## Declaration of generative AI and AI-assisted technologies in the writing process

During the preparation of this work, the authors used ChatGPT in order to shorten the manuscript. No content was generated using this tool—its language editing capabilities were used to increase the clarity and conciseness of the existing text. After using this tool or service, the authors reviewed and edited the content as needed and take full responsibility for the content of the publication.

## STAR★Methods

### Key resources table

**Table:** 

REAGENT or RESOURCE	SOURCE	IDENTIFIER
**Biological samples**		

Plasma samples from CLWH and UCs	CHANGES Bone study^19^	N/A

**Deposited data**		

CHANGES Metabolomics	Biostudies	S-BSST2917 (Biostudies: https://doi.org/10.6019/S-BSST2917)

**Software and algorithms**		

R (v4.2.1)	R Core Team^121^	https://www.r-project.org/
MetaboAnalyst (v6.0)	Xia et al., 2009^122^	https://www.metaboanalyst.ca/
tidyverse	Wickham et al., 2019^123^	https://www.tidyverse.org/
ggpubr	Kassambara, 2023^124^	https://cran.r-project.org/web/packages/ggpubr/index.html
rstatix	Kassambara, 2023^125^	https://cran.r-project.org/web/packages/rstatix/index.html
ggplot2	Wickham et al., 2016^126^	https://ggplot2.tidyverse.org/
plotly	Sievert, 2020^127^	https://plotly.com/
ggstatsplot	Patil, 2021^128^	https://cran.r-project.org/web/packages/ggstatsplot/index.html
ggvendiagram	Gao and Dusa, 2024^129^	https://cran.r-project.org/web/packages/ggVennDiagram/readme/README.html
EnhancedVolcano	Blighe et al., 2022^130^	https://bioconductor.org/packages/devel/bioc/vignettes/EnhancedVolcano/inst/doc/EnhancedVolcano.html

### Experimental model and study participant details

This study includes prepubertal (Tanner stage 1), black African CLWH and children who are HIV uninfected, enrolled in the cohort study Childhood HAART Alterations in Normal Growth, Genes, and aGing Evaluation Study (CHANGES) conducted in Johannesburg, South Africa (reported in detail elsewhere^19^^,^^40^^,^^41^^,^^42^^,^^43^^,^^44^^,^^45^^,^^46^^,^^47^). The Institutional Review Board of the University of the Witwatersrand (Johannesburg, South Africa, M220875) approved this study. For this analysis, we include 400 participants from a sub-study on Bone health as previously published.^19^^,^^47^ Of these, 207 were CLWH and 193 were uninfected controls (UCs). The majority of CLWH in CHANGES started ART before two years of age (median 8.52 months of age) and most had been part of a randomized clinical trial to either stay on their initial lopinavir boosted ritonavir (LPV/r) ART regimen or to switch to an Efavirenz (EFV) based regimen.^20^ Metabolomics data were generated using samples obtained at approximately 8 years of age. The sex distribution of participants in each comparison is provided under the results section, but no information on gender was available.

### Method details

#### Untargeted metabolomics analyses

Plasma samples from the CHANGES cohort (*n* = 400) were sent to Metabolon, Incorporated (Durham, NC, USA) for untargeted metabolomics analyses using their Global Assay. This assay makes use of an ultra-high performance liquid chromatography-tandem mass spectrometer and yields metabolite annotations at the highest confidence level (as previously described).^131^ The details of sample preparation and data collection are provided in Methods S1.

#### Clinical and other measurements

Several other data were available for the CHANGES cohort at the time point where the metabolomics analysis was performed. These included CD4 T cell counts and percentage (CD4%) calculations (determined using the TruCount method, BD Biosciences, Germany), viral load (VL, determined by the Abbott RealTime HIV assay, Abbott Park, Illinois, USA), anthropometric measurements, conventional cholesterol measurements (triglycerides, total cholesterol, LDL, HDL), and bone mass measurements (determined by dual-energy X-ray absorptiometry). The circulating HIV reservoir had also been measured, by proxy, using the HIV PCR Ct scores on samples from a timepoint approximately two years after that of the metabolomics analyses reported here, and has been published.^42^ A history of prior viral loads on ART and pre-ART characteristics were available.

### Quantification and statistical analysis

#### Data mining and statistical analysis strategy

The batch-corrected dataset from Metabolon was prepared for statistical analysis by the application of a 50% zero filter, replacement of missing values by one-fifth of the minimum value for each variable, square root transformation, and Pareto scaling before statistical analysis (MetaboAnalyst 6.0). No additional filtering of variables was performed. Unannotated metabolites and xenobiotics were not removed from the dataset, except for those labeled as ARVs (as these would mly outperform metabolites in distinguishing treatment groups), to maximize the biological insights that could be obtained from the data. It is acknowledged that this does not account for unannotated metabolites that may be metabolites of ARVs. Xenobiotics may yield biological insight, especially relating to shared host-microbiota metabolism and the potential effects of ARVs on biotransformation enzymes. Asterisks at the end of metabolite names indicate features that were confidently identified by Metabolon, but which could not be confirmed using a reference standard.

Statistical analysis was performed in MetaboAnalyst 6.0 and R Studio [R Statistical Software v4.2.1; R Core Team]. MetaboAnalyst’s one-factor module was used for basic statistical analysis, the biomarker analysis module for receiver operating characteristic (ROC) analysis, and the pathway analysis and enrichment analysis modules to aid the biological interpretation. For additional statistical analyses, base R functions and the following R packages were used for some statistical analyses: tidyverse,^123^ ggpubr,^124^ and rstatix,^125^ unless another package is specified. The cut-off values for statistical significance in each test are listed in the results section for each comparison. Figures were generated using MetaboAnalyst, base R, and the following R packages: ggplot2,^126^ plotly,^127^ ggstatsplot,^128^ ggvendiagram,^129^ and EnhancedVolcano.^130^

Partial correlations (with covariate correction, as appropriate to the comparison) were done between various metadata (body mass index, age, sex, CD4 count and percentage, and age at ART initiation) and metabolites, as well as between metabolites (considered significant when the correlation coefficient exceeded ±0.5 and FDR <0.05), using MetaboAnalyst.

Differences in clinical variables between the groups were assessed in GraphPad Prism (version 8.0.1.) by Fisher’s exact test or the Chi-square test for categorical variables, while continuous variables were assessed by either the Wilcoxon rank sum (WRS) or Kruskal-Wallis with Dunn’s test (KWD) as post-hoc analysis.

#### Strategy and stratifications

A biphasic approach was taken for data analysis, wherein the main patterns in the data were first explored via various stratifications and correlations, after which the identified patterns were examined in more detail. During the exploratory phase, using the clinical measurements as metadata, it was found that the ART regimen was, besides HIV status, one of the most prominent factors causing metabolic divergence between CLWH. Stratification by sex also gave statistically robust results, with consideration for the decrease in sample size that occurs with stratification. To control the prominent effects of the ART regimen for an exploration of metabolic factors that may contribute to control, additional analyses were performed within a select group of the EFV-CLWH only, which was the larger of the two treatment groups. Additionally, we investigated whether it was possible to identify a set of metabolic alterations that are common to CLWH irrespective of treatment regimen, which may contribute to hypothesis generation for metabolic biomarkers of viral control for cure and control studies. Thus, four sets of comparisons were made: to characterize the effects of different ART regimens (comparison 1), the effect of sex in UCs and the influence of ART on the metabolic presentation of sex (comparison 2), to identify natural metabolic patterns, or metabotypes, within long-term well controlled CLWH on the same ART regimen (EFV-LTWC, comparison 3), and to identify metabolic alterations common to all CLWH, which showed no divergence based on ART regimen (comparison 4). The association of clinical factors with metabolites was also determined for each of these comparisons.

To minimize the influence of known variables that affect the metabolome, CLWH participants were only selected for analysis if they were in Tanner stage 1 (i.e., prepubescent) and could be considered virally suppressed irrespective of the sensitivity of the test used. That is, if they had less than 1000 HIV RNA copies/ml blood at the time point, used for metabolomics sample collection, or the closest available timepoint. This yielded a group of pre-pubescent, virally suppressed CLWH, which was supplemented with only the UCs who were in Tanner stage 1. The resulting group (*n* = 341) could then be stratified by HIV status (this was the starting point for the exploratory analyses), treatment regimen (in the case of the CLWH), and sex.

#### Marker selection for comparison 1

For comparison 1, stratification by ART regimen, several statistical tests with a variety of mathematical bases were applied, as appropriate (where assumptions were met and where models could be validated satisfactorily). This yielded several lists of statistically significant metabolites, with potential importance in explaining the variance between groups. This was done to avoid the bias any one statistical test might have toward variables with certain characteristics. As such, metabolites that perform well in statistical tests most frequently are highlighted as those with the most potential to have biological meaning. The final list of metabolites that was used for biological interpretation was created by selecting only metabolites that were significant based on three or more statistical measures (using a Venn diagram). The specific tests used for marker selection, as well as the final list for each comparison, can be found in Table S25. The range of tests applied for marker selection for Comparison 1 included fold change analysis (FC; for CLWH-EFV/CLWH-LPV: 0.5<FC > 2), Wilcoxon rank-sum test (WR) with FDR<0.05 and effect sizes calculated as r^132^ or KWD with FDR<0.05 and effect size calculated as Eta-squared^133^ (very small effect: ES < 0.01, small: 0.01 ≤ ES < 0.06 medium: 0.06 ≤ ES < 0.14, large: ES ≥ 0.14),^134^ partial least squares-discriminant analysis [PLS-DA; average variable influence on projection (VIP) for the optimal number of components based on cross-validation >1.5]. PLS-DAs (Figures S6 and S7) were only used for marker selection when Q2>0.5 in cross-validation and when the model was not random, as indicated by permutation testing using prediction accuracy during training (*p* < 0.05, *n* = 2000). For additional exploration, and to cross-reference the performance of metabolite markers, orthogonal partial least squares discriminant analysis (OPLS-DA; VIP>1.5), and random forests analysis [RF; top 50 based on mean decrease accuracy, if the out-of-bag (OOB) error was minimal].

#### Marker selection for comparison 2

Given that the comparisons by sex were expected to show smaller differences due to participants being pre-pubescent and the reduction in sample size due to the necessary stratification, these comparisons were done using tests more appropriate to smaller sample sizes. Criteria for marker selection were KWD FDR<0.05 (48 metabolites), alongside effect size calculated as Eta-squared^133^^,^^134^ (as described above, six metabolites fulfilled both criteria) (Table S26). An additional nine metabolites had KWD 0.05<FDR<0.06 (approaching significance), and these were used only to gain additional biological context. PCA and PLS-DA scores plots were used only to explore the overarching patterns in the data (Figure S10).

#### Marker selection for comparison 3

Within the CHANGES, the group of children most relevant to the cure context are those who maintained viral suppression over time and achieved immune reconstitution on ART. However, immune reconstitution could only be assessed by absolute CD4 count and CD4% for the CHANGES, as these were the only available parameters. Longitudinal viral loads and CD4% were plotted over the time [all available time points since the first achievement of viral suppression (VL < 50) in infancy to the end of follow-up time for CHANGES around early adolescence] for each participant and the area under the curve (AUC) was determined (using the R package “DescTools”^135^). This AUC was then normalized to the total follow-up time for each participant (AUC/follow-up time in months), as there were differences in the available data for each participant depending on the date of initial viral suppression and the availability of data from subsequent visits, to obtain a VL and CD4% score for each participant (Tables S9 and S10). These scores were then compared across groups (described below) using WR and were correlated to other clinical parameters and metabolites. The details of the creation of these variables and their analyses can be found in Data S4.

For comparison 3, a subset of the prepubescent CLWH was selected based on long-term viral suppression [undetectable viral load with occasional blipping, for >6 years, with no blips >1000 HIV RNA copies/ml]. Participants had to have an undetectable viral load (<50 copies/ml) at both the metabolomics and HIV DNA PCR timepoints for inclusion. This was to avoid the effect of active viral replication on the metabolome and to minimize the effect of HIV RNA on the HIV DNA PCR Ct value, which could then be taken as a proxy for the circulating HIV reservoir. This yielded a group of children on both an EFV or LPV-based ART regimen (*n* = 73). Given the extent to which the treatment regimen affects the metabolic profile, the well-controlled CLWH were separated by treatment regimen. As the EFV-CLWH (*n* = 49) was much larger than the LPV-CLWH (*n* = 24), the long-term well-controlled EFV-CLWH group (now referred to as the EFV-LTWC) was selected for further analyses, as this would allow more reliable statistical comparisons. All children in the EFV-LTWC group were on EFV with 3 TC and ABC. Within this group, seven participants had not achieved a CD4% within the 10^th^ percentile of the age group (31%).^23^ These children were not excluded, to observe their natural grouping based on the metabolic profile, but the analyses described below were performed with and without them.

A data-driven (unsupervised) approach was followed, using hierarchical clustering analysis (HCA, similarity measure: Euclidean distance, algorithm: Ward’s linkage), to determine if natural metabolic subtypes existed within the EFV-LTWCs. This was verified by an alternative clustering method, K-means clustering, by specifying the cluster number as two. The identified metabotypes were then compared to each other and 50 age- and sex-matched UCs (matched with the nearest neighbor method based on Euclidean distance using the R package “MatchIt”,^136^; Tables S6 and S7) using the KWD with effect sizes, PCA, and PLS-DA (Figure S12) to determine the metabolite patterns that gave rise to the metabotypes. The association of metabotypes with clinical parameters was also assessed via correlations and the KWD (with FDR<0.05). Those metabolites with KWD FDR<0.05, 0.5<FC > 2, and PLS-DA VIP>1.5 were considered as best describing the difference between the metabotypes, but all significant metabolites from the KWD were interpreted to facilitate the elucidation of the underlying mechanism of the metabolic divergence among EFV-LTWC (Table S28).

#### Marker selection for comparison 4

To identify markers associated with HIV or its direct consequences, it was necessary to exclude metabolites that showed significant divergence based on the ART treatment regimen. Two approaches were used to increase the robustness of the identified markers. Firstly, the KWD results from comparison 2 allowed the identification of metabolites that were statistically significantly different from UCs with the same trend (both increased or both decreased relative to UCs) in both the EFV- and LPV-CLWH groups (that is, significant in UC vs. EFV and in UC vs. LPV, but not in EFV vs. LPV, Figures S14A–S14C). These metabolites were more likely to be altered due to HIV, or its consequences, as opposed to ART. To assess if these metabolite alterations were eclipsed by the effect of ART when the CLWH were not stratified by ART, this set of metabolites were compared to the set of metabolites that best described the difference between the UCs and the unstratified CLWH. This allowed the identification of metabolites potentially associated with the effect of HIV that reach statistical significance even in a CLWH group on more than one regimen (Figure S14D). To see if a different approach would yield comparable results, two classification methods (RF and support vector machine ROC) were used, and the metabolites with the best combined performance in these models were further assessed (Figure S15). The metabolites identified by both approaches were all assessed for qualitative (increased/decreased relative to UCs) and quantitative (similar levels between EFV and LPV-CLWH, but different from UCs) similarities in the difference from the UCs. Thus, metabolites were only considered related to the effect of HIV if the direction and extent of the difference from UCs were comparable between the EFV and LPV-CLWH (Table S16). As the results of this classification approach yielded several unannotated metabolites, and it was not possible to identify these metabolites for this study, their annotated names (as supplied by Metabolon) were searched against the National Human Genome Research Institute-European Bioinformatics Institute Catalog of human genome-wide association studies and PubMed to generate hypotheses as to their biological context from previous studies using Metabolon data (Table S16).

## Supplemental information

Document S1. Figures S1–S20, Tables S1–S23, Data S1–S7, and Methods S1

Table S26. Summary of all statistics applied for comparison 2 - UC-F vs. UC-M

Table S29. Metabolites best describing the metabolic effects of HIV irrespective of the treatment regimen based on classification models
