# Supplementary material for: Antiretroviral regimen and sex-specific metabolic heterogeneity in pediatric HIV
Source: iScience. 2026 Jul 20;29(8):116865. doi: 10.1016/j.isci.2026.116865 (PMC13393711; doi:10.1016/j.isci.2026.116865)
Supplement: Document S1. Figures S1–S20, Tables S1–S23, Data S1–S7, and Methods S1 [file mmc1.pdf]

iScience, Volume *29*

Supplemental information

Antiretroviral regimen and sex-specific metabolic heterogeneity in pediatric HIV

Chandre Herbert, Louise Kuhn, Nicole H. Tobin, Fan Li, Renate Strehlau, Faeezah Patel, Tian Wang, Shuang Wang, Grace M. Aldrovandi, and Caroline T. Tiemessen

Methods S1

Details of metabolomics analysis as performed by Metabolon

This is an extract from Metabolon's mData Report™ for the study, "Metabolomic Analysis of the CHANGES Bone Study Cohort":

Metabolon Platform

Sample Accessioning: Following receipt, samples were inventoried and immediately stored at -80°C. Each sample received was accessioned into the Metabolon LIMS system and was assigned by the LIMS a unique identifier that was associated with the original source identifier only. This identifier was used to track all sample handling, tasks, results, etc. The samples (and all derived aliquots) were tracked by the LIMS system. All portions of any sample were automatically assigned their own unique identifiers by the LIMS when a new task was created; the relationship of these samples was also tracked. All samples were maintained at -80°C until processed.

Sample Preparation: Samples were prepared using the automated MicroLab STAR® system from Hamilton Company. Several recovery standards were added prior to the first step in the extraction process for QC purposes. To remove protein, dissociate small molecules bound to protein or trapped in the precipitated protein matrix, and to recover chemically diverse metabolites, proteins were precipitated with methanol under vigorous shaking for 2 min (Glen Mills GenoGrinder 2000) followed by centrifugation. The resulting extract was divided into multiple fractions: two for analysis by two separate reverse phase (RP)/UPLC-MS/MS methods with positive ion mode electrospray ionization (ESI), one for analysis by RP/UPLC-MS/MS with negative ion mode ESI, one for analysis by HILIC/UPLC-MS/MS with negative ion mode ESI, while the remaining fractions were reserved for backup. Samples were placed briefly on a TurboVap® (Zymark) to remove the organic solvent. The sample extracts were stored overnight under nitrogen before preparation for analysis.

QA/QC: Several types of controls were analyzed in concert with the experimental samples: a pooled matrix sample generated by taking a small volume of each experimental sample (or alternatively, use of a pool of well-characterized human plasma) served as a technical replicate throughout the data set; extracted water samples served as process blanks; and a cocktail of QC standards that were carefully chosen not to interfere with the measurement of endogenous compounds were spiked into every analyzed sample, allowed instrument performance monitoring and aided chromatographic alignment. **Tables 1** and **2** describe these QC samples and standards. Instrument variability was determined by calculating the median relative standard deviation (RSD) for the standards that were added to each sample prior to injection into the mass spectrometers. Overall process variability was determined by calculating the median RSD for all endogenous metabolites (*i.e.,* non-instrument standards) present in 100% of the pooled matrix samples. Experimental samples were randomized across the platform run with QC samples spaced evenly among the injections, as outlined in **Figure 1** below.

Table S1 Description of Metabolon QC Samples

Type	Description	Purpose
MTRX	Large pool of human plasma maintained by Metabolon that has been characterized extensively.	Assure that all aspects of the Metabolon process are operating within specifications.
CMTRX	Pool created by taking a small aliquot from every customer sample.	Assess the effect of a non-plasma matrix on the Metabolon process and distinguish biological variability from process variability.
PRCS	Aliquot of ultra-pure water	Process Blank used to assess the contribution to compound signals from the process.

Table S2 Metabolon QC Standards.

Type	Description	Purpose
RS	Recovery Standard	Assess variability and verify performance of extraction and instrumentation.
IS	Internal Standard	Assess variability and performance of instrument.

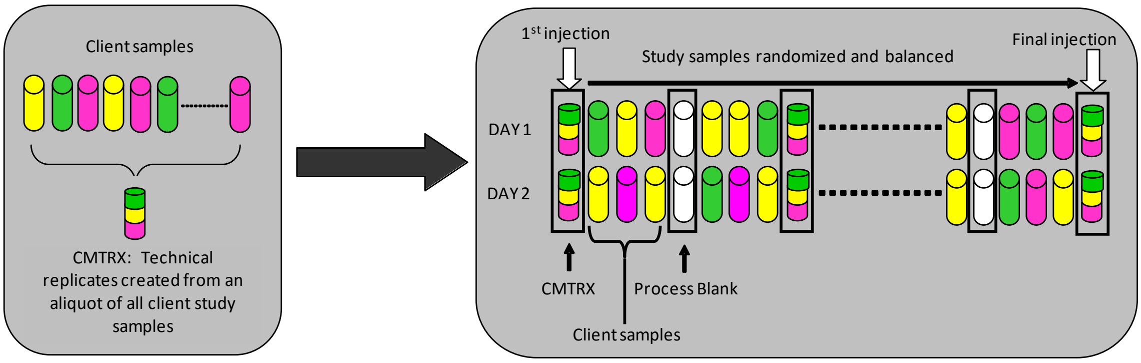

Figure S1 Preparation of client-specific technical replicates. A small aliquot of each client sample (colored cylinders) is pooled to create a CMTRX technical replicate sample (multi-colored cylinder), which is then injected periodically throughout the platform run. Variability among consistently detected biochemicals can be used to calculate an estimate of overall process and platform variability.

Ultrahigh Performance Liquid Chromatography-Tandem Mass Spectroscopy (UPLC-MS/MS): All methods utilized a Waters ACQUITY ultra-performance liquid chromatography (UPLC) and a Thermo Scientific Q-Exactive high resolution/accurate mass spectrometer interfaced with a heated electrospray ionization (HESI-II) source and Orbitrap mass analyzer operated at 35,000 mass resolution (PMID: 32445384). The dried sample extracts were then reconstituted in solvents compatible to each of the four methods. Each reconstitution solvent contained a series of standards at fixed concentrations to ensure injection and chromatographic consistency. One aliquot was analyzed using acidic positive ion conditions, chromatographically optimized for more hydrophilic compounds (PosEarly). In this method, the extract was gradient eluted from a C18 column (Waters UPLC BEH C18-2.1x100 mm, 1.7 µm) using water and methanol, containing 0.05% perfluoropentanoic acid (PFPA) and 0.1% formic acid (FA). Another aliquot was also analyzed using acidic positive ion conditions, however it was chromatographically optimized for more hydrophobic compounds (PosLate). In this method, the extract was gradient eluted from the same aforementioned C18 column using methanol, acetonitrile, water, 0.05% PFPA and 0.01% FA and was operated at an overall higher organic content. Another aliquot was analyzed using basic negative ion optimized conditions using a separate dedicated C18 column (Neg). The basic extracts were gradient eluted from the column using methanol and water, however with 6.5mM Ammonium Bicarbonate at pH 8. The fourth aliquot was analyzed via negative ionization following elution from a HILIC column (Waters UPLC BEH Amide 2.1x150 mm, 1.7 µm) using a gradient consisting of water and acetonitrile with 10mM Ammonium Formate, pH 10.8 (HILIC). The MS analysis alternated between MS and data-dependent MS^n scans using dynamic exclusion. The scan range varied slightly between methods but covered 70-1000 m/z. Raw data files are archived and extracted as described below.

Bioinformatics: The informatics system consisted of four major components, the Laboratory Information Management System (LIMS), the data extraction and peak-identification software, data processing tools for QC and compound identification, and a collection of information interpretation and visualization tools for use by data analysts. The hardware and software foundations for these informatics components were the LAN backbone, and a database server running Oracle 10.2.0.1 Enterprise Edition.

LIMS: The purpose of the Metabolon LIMS system was to enable fully auditable laboratory automation through a secure, easy to use, and highly specialized system. The scope of the Metabolon LIMS system encompasses sample accessioning, sample preparation and instrumental analysis and reporting and advanced data analysis. All of the subsequent software systems are grounded in the LIMS data structures. It has been modified to leverage and interface with the in-house information extraction and data visualization systems, as well as third party instrumentation and data analysis software.

Data Extraction and Compound Identification: Raw data was extracted, peak-identified and QC processed using a combination of Metabolon developed software services (applications). Each of these services perform a specific task independently, and they communicate/coordinate with each other using industry-standard protocols. Compounds were identified by comparison to library entries of purified standards or recurrent unknown entities. Metabolon maintains a library based on authenticated standards that contains the retention time/index (RI), mass to charge ratio (*m/z)*, and fragmentation data on all molecules present in the library. Furthermore, biochemical identifications are based on three criteria: retention index within a narrow RI window of the proposed identification, accurate mass match to the library +/- 10 ppm, and the MS/MS forward and reverse scores between the experimental data and authentic standards. The MS/MS scores are based on a comparison of the ions present in the experimental spectrum to the ions present in the library spectrum. While there may be similarities between molecules based on one of these factors, the use of all three data points is utilized to distinguish and differentiate biochemicals. More than 5,400 commercially available purified or in-house synthesized standard compounds have been acquired and analyzed on all platforms for determination of their analytical characteristics. An additional 7000 mass spectral entries have been created for structurally unnamed biochemicals, which have been identified by virtue of their recurrent nature (both chromatographic and mass spectral). These compounds have the potential to be identified by future acquisition of a matching purified standard or by classical structural analysis. Metabolon continuously

adds biologically-relevant compounds to its chemical library to further enhance its level of Tier 1 metabolite identifications.

Compound Quality Control: A variety of curation procedures were carried out to ensure that a high-quality data set was made available for statistical analysis and data interpretation. The QC and curation processes were designed to ensure accurate and consistent identification of true chemical entities, and to remove or correct those representing system artifacts, mis-assignments, mis-integration and background noise. Metabolon data analysts use proprietary visualization and interpretation software to confirm the consistency of peak identification and integration among the various samples.

Metabolite Quantification and Data Normalization: Peaks were quantified using area-under-the-curve. For studies spanning multiple days, a data normalization step was performed to correct variation resulting from instrument inter-day tuning differences. Essentially, each compound was corrected in run-day blocks by registering the medians to equal one (1.00) and normalizing each data point proportionately (termed the "block correction", **Figure 2**). For studies that did not require more than one day of analysis, no normalization is necessary, other than for purposes of data visualization. In certain instances, biochemical data may have been normalized to an additional factor (*e.g.,* cell counts, total protein as determined by Bradford assay, osmolality, etc.) to account for differences in metabolite levels due to differences in the amount of material present in each sample.

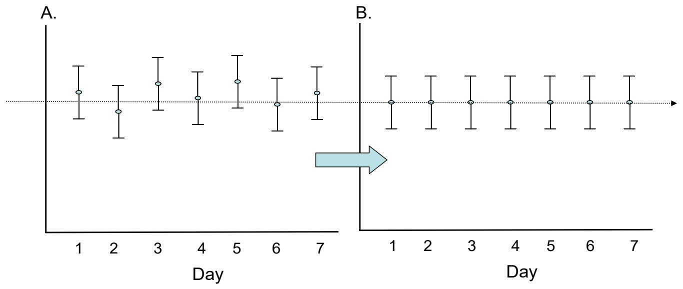

Figure S2 Visualization of data normalization steps for a multiday platform run.

Table S3: Detailed composition of ART regimens and their frequencies in the pre-pubescent, virally suppressed metabolomics subset of CHANGES (related to Table 1)

Regimen abbreviation	NNRTI	NRTI 1	NRTI 2	PI	CHANGES CLWH [no (%)]
**Efavirenz-based regimens**					
EFV, 3TC, ABC	Efavirenz	Abacavir	Lamivudine		96 (95.05)
EFV, 3TC, D4T	Efavirenz	Stavudine	Lamivudine		4 (3.96)
EFV, 3TC, AZT	Efavirenz	Zidovudine	Lamivudine		1 (0.99)
**Total on an EFV-based regimen**					101 (65.16)
**Lopinavir/ritonavir-based regimens**					
LPV/r, 3TC, ABC		Abacavir	Lamivudine	Lopinavir/ritonavir	52 (96.30)
LPV/r, 3TC, AZT		Zidovudine	Lamivudine	Lopinavir/ritonavir	1 (1.85)
EFV, LPV/r, 3TC	Efavirenz		Lamivudine	Lopinavir/ritonavir	1 (1.85)
**Total on an LPV/r-based regimen**					54 (34.84)

Abbreviations: NNRTI: non-nucleoside reverse transcriptase inhibitor, NRTI: nucleoside reverse transcriptase inhibitor, PI: protease inhibitor, EFV: efavirenz, 3TC: lamivudine, ABC: abacavir, D4T: stavudine, AZT: zidovudine, LPV/r: lopinavir/ritonavir.

Data S1: Exploratory analyses

UC vs CLWH (a priori exclusion of those in Tanner stages 2 or 3 and those with VL>1000)

A

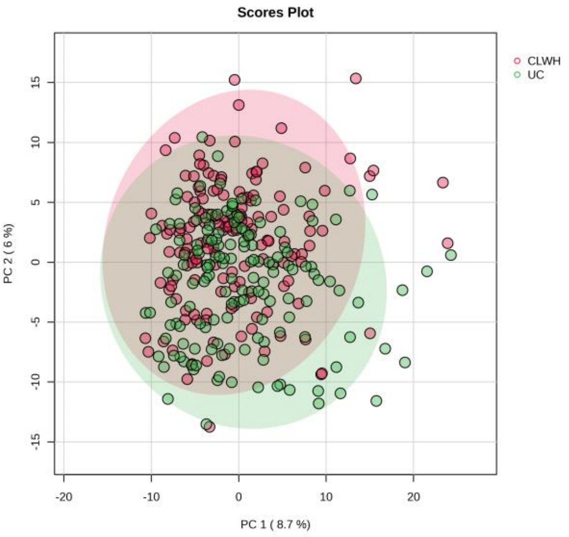

B

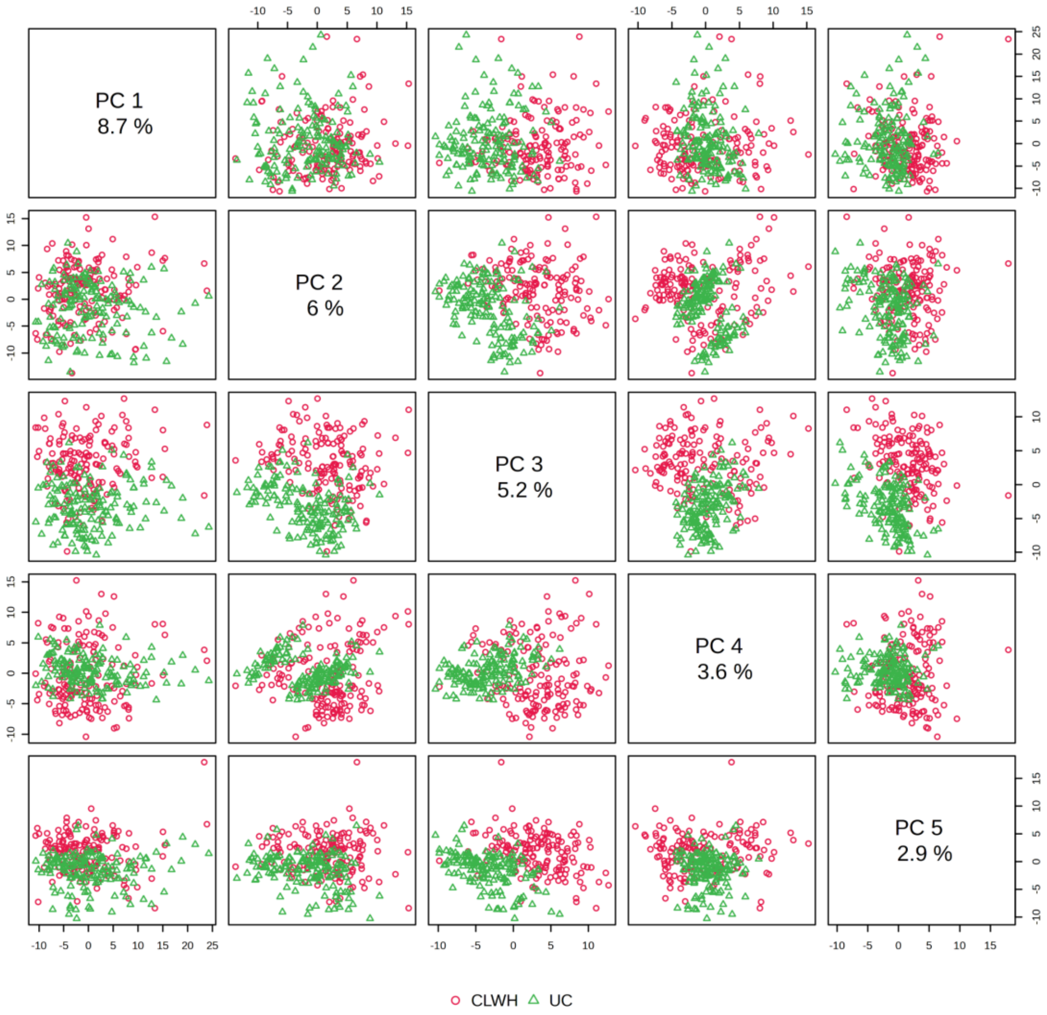

Figure S3 Results of the PCA for the exploratory analyses. A: PCA scores plot (PC1 vs PC2) and B: Overview of the first five principal components scores, plotted against each other. While the UCs and CLWH do not separate in the first two components, implying that many aspects of their metabolomes are similar, there are clear, albeit smaller, metabolic differences between the UCs and CLWH, as seen in the other PC scores plots. (Related to Fig 2).

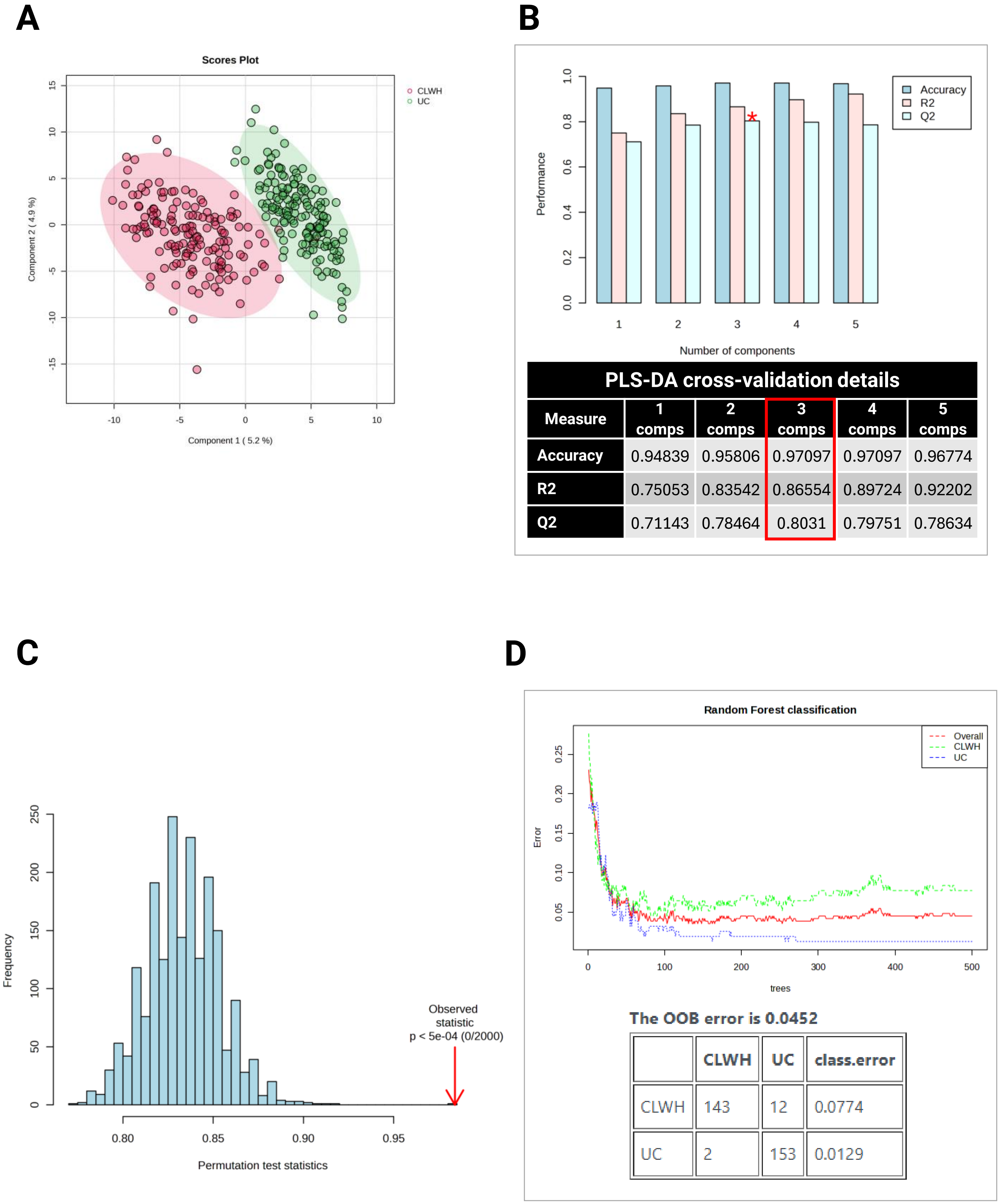

PLS-DA cross-validation details					
Measure	1 comps	2 comps	3 comps	4 comps	5 comps
Accuracy	0.94839	0.95806	0.97097	0.97097	0.96774
R2	0.75053	0.83542	0.86554	0.89724	0.92202
Q2	0.71143	0.78464	0.8031	0.79751	0.78634

The OOB error is 0.0452

	CLWH	UC	class.error
CLWH	143	12	0.0774
UC	2	153	0.0129

Figure S4 Graphical results of the PLS-DA and RF analysis for the exploratory analyses (UC vs CLWH) **A**: PLS-DA scores plot, showing separation between the UCs and the CLWH., **B**: PLS-DA five-fold cross-validation results. The model performed best with three components and was not overfitted. **C**: PLS-DA permutation testing results. The test statistic was prediction accuracy during testing, performed with 2000 permutations. The observed statistic was p<0,0005 (0/2000 permutations). This implies that the fitted model is unlikely to have occurred by random chance. **D:** RF classification plot (showing the error plotted against the number of trees) and the confusion matrix, showing that the out-of-bag error was 0,04. This means that samples could frequently be correctly classified based on the metabolome.

(Related to Fig 2).

The effect of detectable VL

Comparisons of CLWH with detectable (VL>20) and undetectable (VL≤20) VL across and between ART groups.

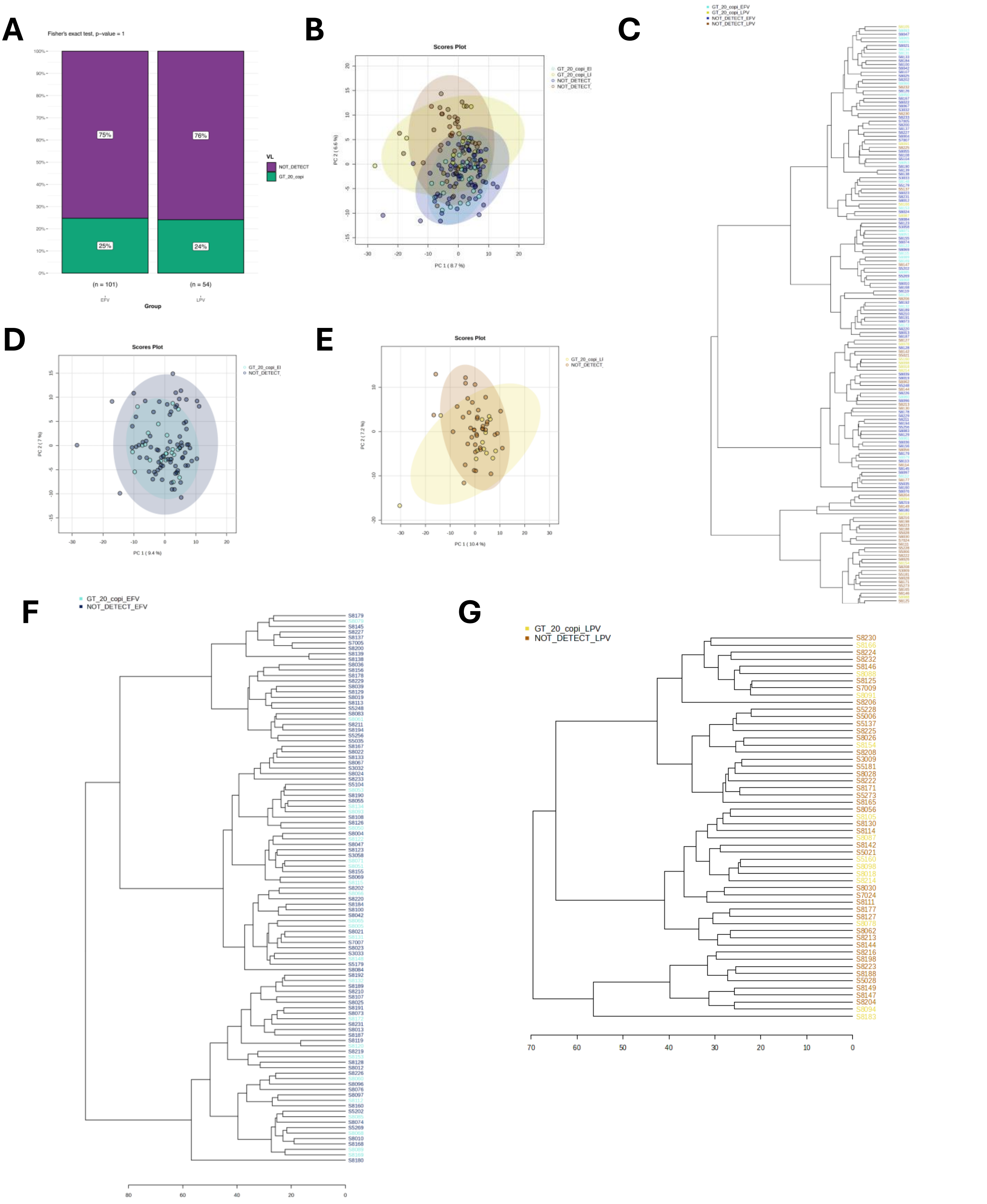

Figure S5 Results of the explorations relating to detectable versus undetectable VL in CLWH, irrespective of treatment group as well as stratified by treatment group and VL. A: A mosaic plot showing that there were no differences in the distribution of viremic and aviremic individuals in the EFV-CLWH and LPV-CLWH. **B**: A PCA plot and dendrogram (**C**) showing that the CLWH did not cluster based on VL, but rather clustered based on treatment regimen. This, along with the PCA plots (**D, E**) and dendrograms (**F, G)** within each treatment group, also shows that the viremic and aviremic individuals did not separate within a treatment group.

- To determine if there were any metabolic trends relating to VL level, the uncorrected WR tests (raw $p<0,05$) along with $0,5<FC>2$ between CLWH were used. No metabolites satisfying these criteria were significant in both treatment group comparisons (EFV-detectable vs EFV-undetectable and LPV-detectable vs LPV-undetectable). When only WR was used, only palmitoyl-arachidonoyl-glycerol (16:0/20:4) [2]*, cis-3,4-methyleneheptanoate, glycochenodeoxycholate were different between viremic and aviremic individuals in both treatment groups. When only FC was considered, eugenol sulfate, 2-hydroxyhippurate (salicylurate), 3-methoxycatechol sulfate (1), 4-vinylcatechol sulfate, 3-hydroxysebacate, salicylate, 4-vinylguaiacol sulfate, X-17690, and X-12007 were different between viremic and aviremic individuals in both treatment groups.

Sensitivity analyses — the effect of the CLWH using slightly different ART regimens

- The sensitivity analyses for the comparison by ART regimen (comparison 2) is reported in full here, as an example, as this is the comparison where the effect of different drugs in the regimen would be most profound.

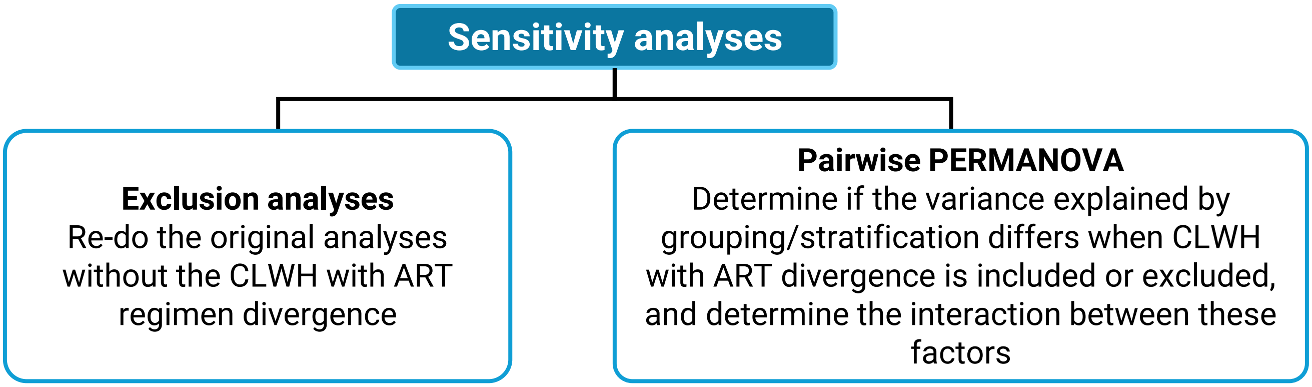

EXCLUSION ANALYSES

- **Kruskal-Wallis** → Including all children (733 metabolites with FDR<0.05), excluding those with ART regimen divergence (736 metabolites with FDR<0.05).
- **Kruskal-Wallis with Dunn's post-hoc (KWD) for EFV vs LPV**→ The first 275 (>50%) significantly different metabolites (FDR<0.05) ranked exactly the same (based on corrected p-values) whether the children with ART divergence were included or not.
- Spearman rank correlation showed a strong and significant correlation between the FDR values with and without the CLWH with ART regimen divergence (ρ=0.9807, confidence interval=0.9763-0.9843, p<0.0001).

Table S4 Summary of the different KWD results when the CLWH with ART regimen divergence were included or excluded in the comparison of EFV-CLWH to LPV-CLWH (Related to "Setting the stage" section of Results).

	Without exclusion	With exclusion
**No. of metabolites with FDR<0.05**	402	416
**No. of metabolites no longer significant after exclusion**	18 (4.5% of 402)<br>• 14 (3.5% of 402) annotated<br>• 4 (1% of 402) unannotated	
**No. of metabolites additionally significant after exclusion**	31 (7.7% of 402)<br>• 26 (6.5% of 402) annotated<br>• 5 (1.2% of 402) unannotated	

PAIRWISE PERMANOVA

Table S5 PERMANOVA models for EFV-CLWH vs LPV-CLWH

	Group		ART divergence	
	**R^2**	**p-value**	**R^2**	**p-value**
**Primary model** (All children, ART regimen group + ART divergence)	0.04544	**0.0001**	0.00897	**0.0376**
**Exclusion model** (Children with ART divergence excluded, ART regimen group)	0.04815	**0.0001**		
**Interaction model**	**R^2**		**p-value**	
(All children, ART regimen group* ART divergence)	0.00771		0.1251	

(Related to "Setting the stage" section of Results)

- While the p-values were significant for the contribution of both the primary ART groups and the ART divergence in explaining the metabolic variance, the primary ART groups explain a significantly larger portion of metabolic variation (4.5% versus 0.9%).
- The R^2 value changed only very slightly when the children with ART divergence were excluded (a change of 0.00271 or approximately 6%), and remained significant.
- There was not a significant interaction between the primary ART groups and ART divergence.

- **In light of these results, together with the exclusion analyses, the effect of ART divergence was considered minimal, even in this comparison where it was likely to have the strongest effect. As such, the children showing ART divergence were not excluded.**

Data S2:
Comparison 1 (UC vs CLWH-EFV vs CLWH-LPV)

A

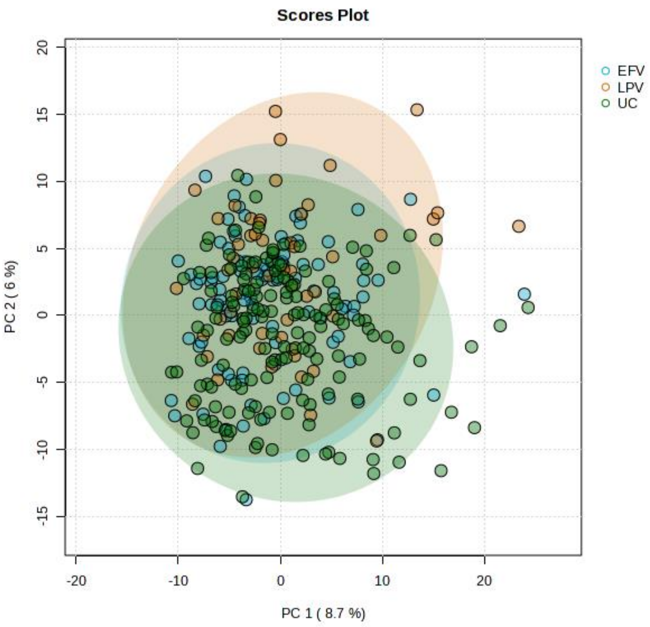

B

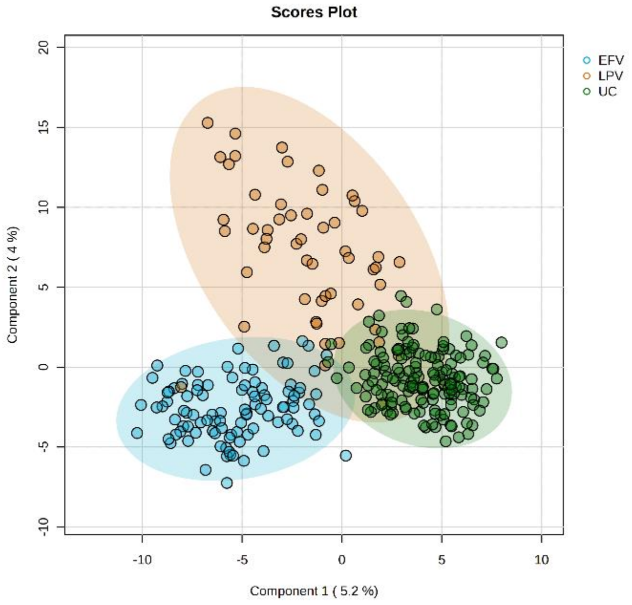

C

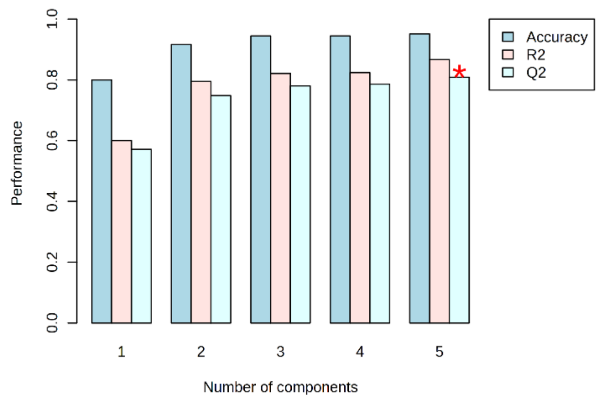

PLS-DA cross-validation details					
Measure	1 comps	2 comps	3 comps	4 comps	5 comps
Accuracy	0.800	0.916	0.945	0.945	0.951
R2	0.600	0.795	0.821	0.824	0.867
Q2	0.571	0.748	0.780	0.786	0.808

D

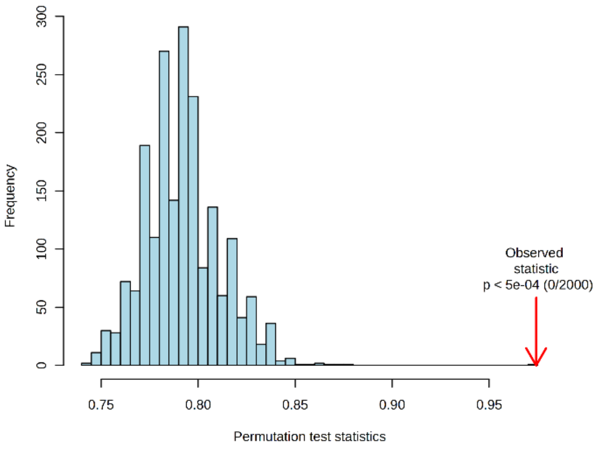

Figure S6 Graphical results of the multivariate statistics for comparison 1 (UC vs CLWH-EFV vs CLWH-LPV). A: PCA scores plot (PC1 vs PC2), B: PLS-DA scores plot, C: PLS-DA five-fold cross-validation results. The model performed best with five components and was not overfitted. Hence, for marker selection, those metabolites with an average VIP score of >1.5, across the first five components, were selected as best representing the difference between the groups based on the PLS-DA, D: PLS-DA permutation testing results. The test statistic was prediction accuracy during testing, performed with 2000 permutations. The observed statistic was p<0,0005 (0/2000 permutations). This implies that the fitted model is unlikely to have occurred by random chance. (Related to Fig 3).

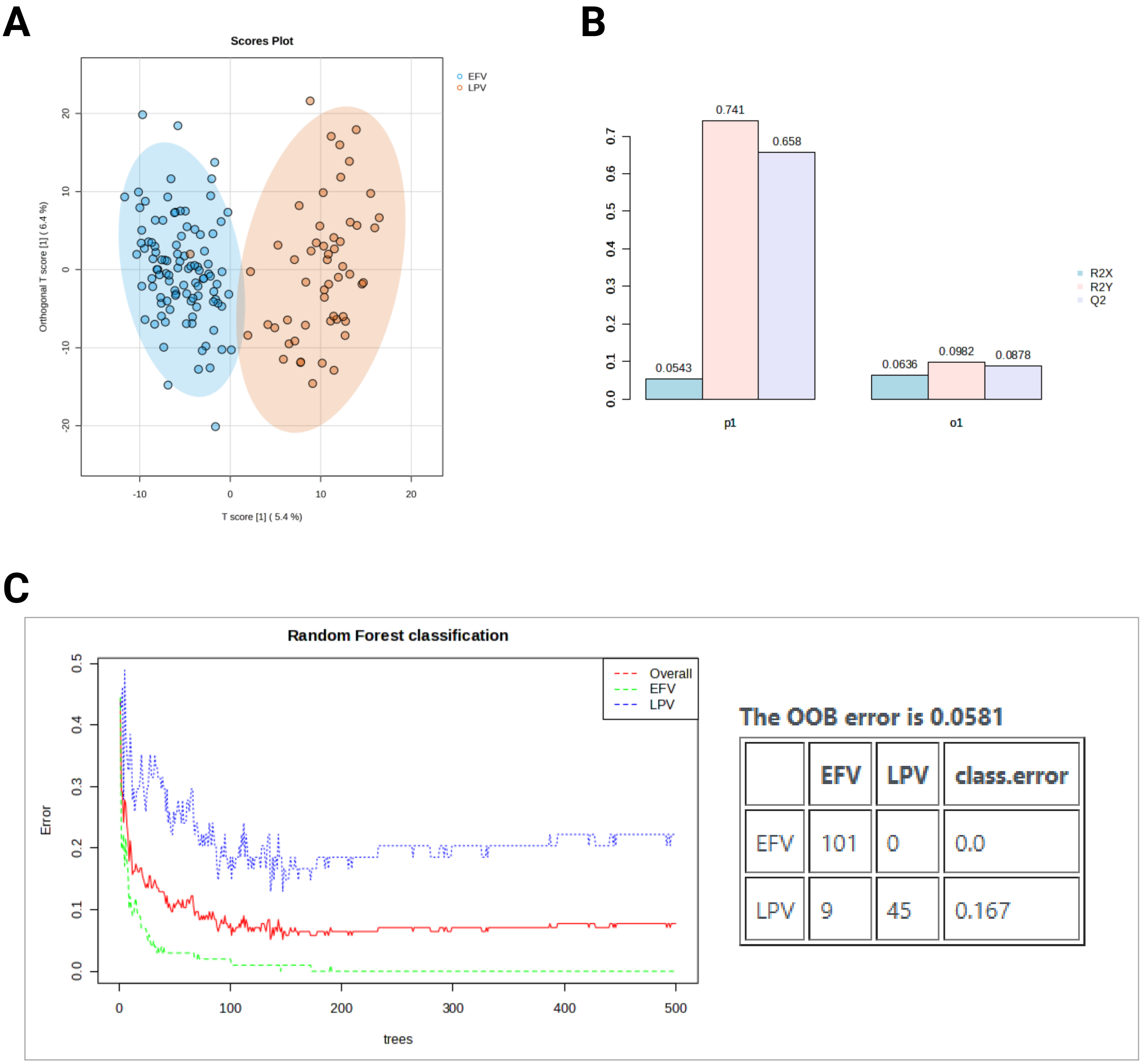

	EFV	LPV	class.error
EFV	101	0	0.0
LPV	9	45	0.167

Figure S7 Graphical results of the additional statistics performed, but not used for marker selection, for comparison 1, when only the CLWH groups are compared to each other (CLWH-EFV vs CLWH-LPV). This was used to corroborate the performance of metabolites best describing the difference between EFV-CLWH and LPV-CLWH, as identified by the marker selection process. **A:** OPLS-DA scores plot **B:** OPLS-DA cross-validation results, showing that the groups separate sufficiently only in component 1 in this model (t-score), **C:** Random forests classification performance graph and the associated confusion matrix and out-of-bag error (0,0581). This analysis was performed using 500 trees and seven predictors. This shows that while all EFV-CLWH were classified correctly, nine of 54 LPV-CLWH were misclassified as being of the EFV-CLWH group. (Related to Fig 3).

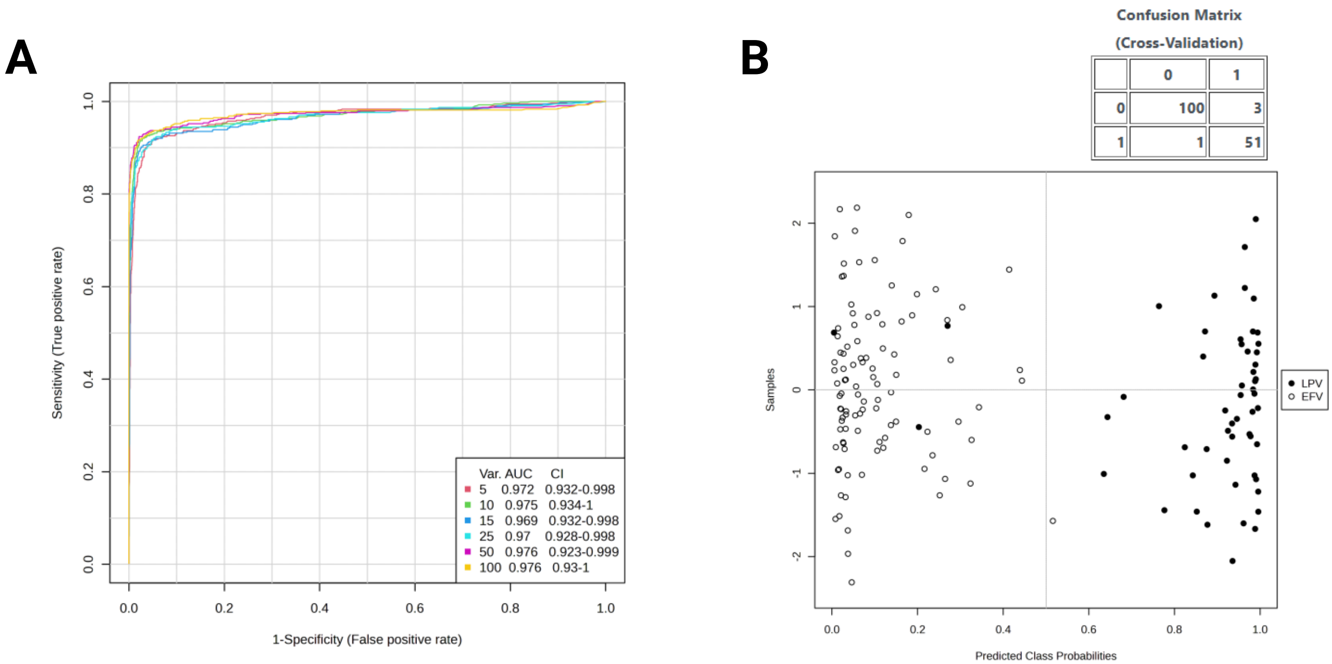

C

Top 10 best-performing metabolites in multivariate ROC analysis (EFV-CLWH vs LPV-CLWH)					
CHEMID	Metabolite	Rank Freq.	Importance	EFV	LPV
999916935	X-16935	1	0.000488	Low	High
100021331	Metabolonic lactone sulfate	1	0.000392	Low	High
100019794	Hydroxy-CMPF*	1	0.00035	Low	High
999924556	X-24556	1	0.000294	Low	High
100004182	3Beta-hydroxy-5-cholenoate	1	0.000266	High	Low
100015968	Carotene diol (3)	1	0.000258	Low	High
100004083	Glycohyocholate	1	0.000202	High	Low
100001073	Androsterone sulfate	1	0.000195	Low	High
100001743	Tryptophan betaine	0.966667	0.000235	High	Low
100001250	Tauro-beta-muricholate	0.966667	0.000221	High	Low

Figure S8 Results and validation of the multivariate ROC analysis performed using the support vector machine algorithm as classification and feature ranking method for distinguishing CLWH treatment groups (CLWH-EFV vs CLWH-LPV). **A:** A ROC curve representing models with 5, 10, 15, 25, 50, or 100 metabolites, to classify CLWH into the EFV or LPV groups. The 100-metabolite model (represented by the yellow curve) performed best (AUC = 0,973; CI: 0,927-1). This suggests that there is substantial interindividual variation even amongst those on the same treatment regimen, as also shown in comparison 3. **B:** The predicted class probabilities (average of the cross-validation) for each sample using the best-performing model (100-metabolite) and the corresponding confusion matrix. **C:** The details of the performance of the top 10 best-performing metabolites in the ROC analysis. Note that the best performing metabolites are an unannotated metabolite (X-16935) and the steroid metabolite, metabolonic lactone sulphate, while this list also includes other steroids, bile acids, and (typically) microbially produced derivatives of tryptophan and provitamin A (carotene). (Related to Fig 3).

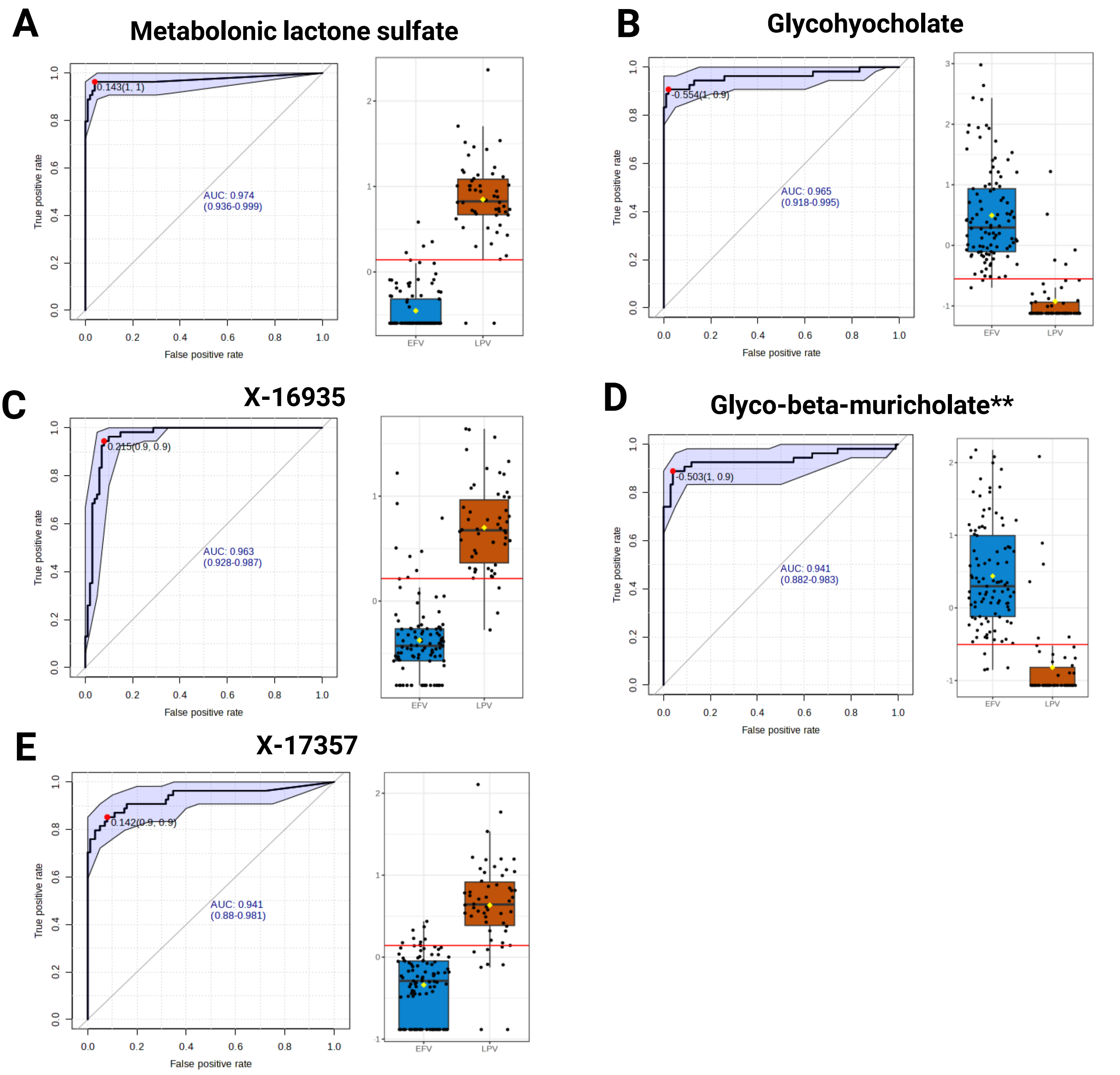

F

Top 10 best-performing metabolites in univariate ROC analysis (EFV-CLWH vs LPV-CLWH)				
**CHEMID**	**Metabolite**	**AUC**	**t-tests**	**Log2 FC**
100021331	metabolonic lactone sulfate	0.97286	1.23E-48	-4.1465
100004083	glycohyocholate	0.96278	2.85E-24	3.7173
999916935	X-16935	0.96095	1.27E-34	-2.6261
100020214	glyco-beta-muricholate**	0.93711	2.72E-21	2.7781
999917357	X-17357	0.93674	1.86E-26	-2.0509
100001073	androsterone sulfate	0.93243	2.02E-26	-3.1945
100001250	tauro-beta-muricholate	0.92923	4.00E-18	3.5973
999924556	X-24556	0.92739	2.60E-28	-2.7878
100019794	hydroxy-CMPF*	0.92061	8.97E-25	-2.1184
100002027	androstenediol (3alpha, 17alpha) monosulfate (3)	0.91135	7.36E-23	-2.3733

Figure S9 Results of the univariate ROC analysis for distinguishing CLWH treatment groups (CLWH-EFV vs CLWH-LPV). **A-E**: ROC curves and boxplots for the top five best-performing metabolites in the univariate ROC, based on AUC. **F**: A summary of the statistics related to the top ten best-performing metabolites in the univariate ROC analysis. Again, this list is dominated by steroids and bile acids. ****:** Indicates a compound for which a standard was not available (not Tier 1 identification), but Metabolon was reasonably confident of its identity, or the information provided. (Related to Fig 3).

Data S3:
Comparison 2 (comparing the sexes across all groups)

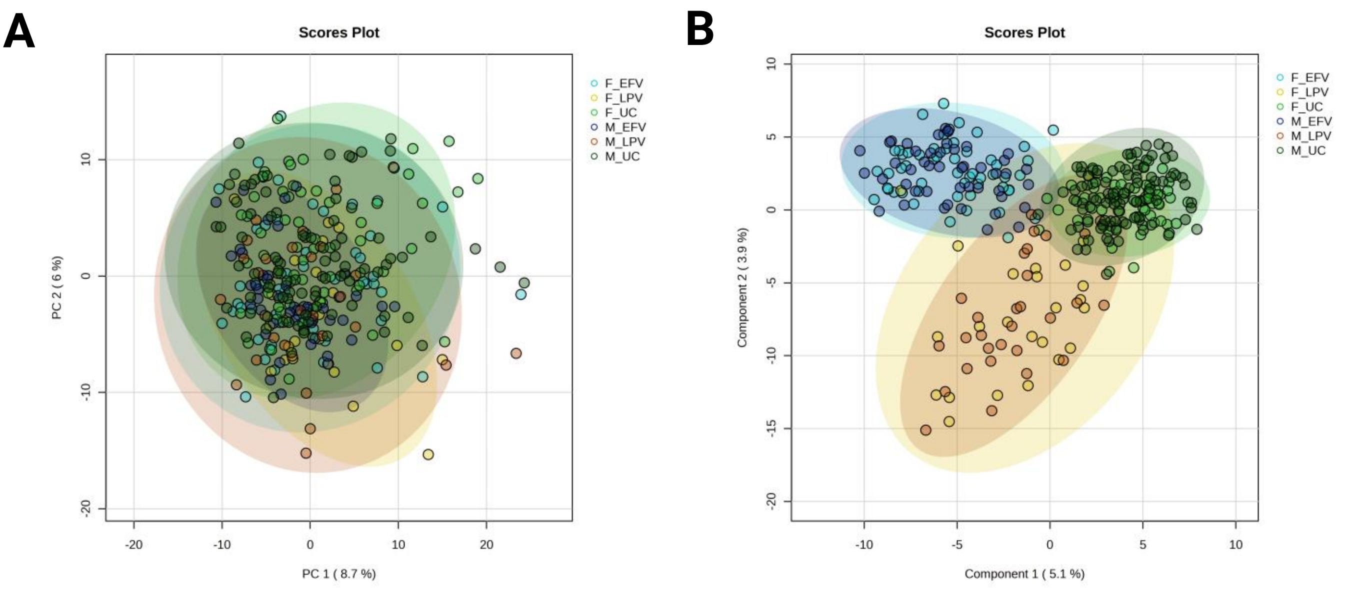

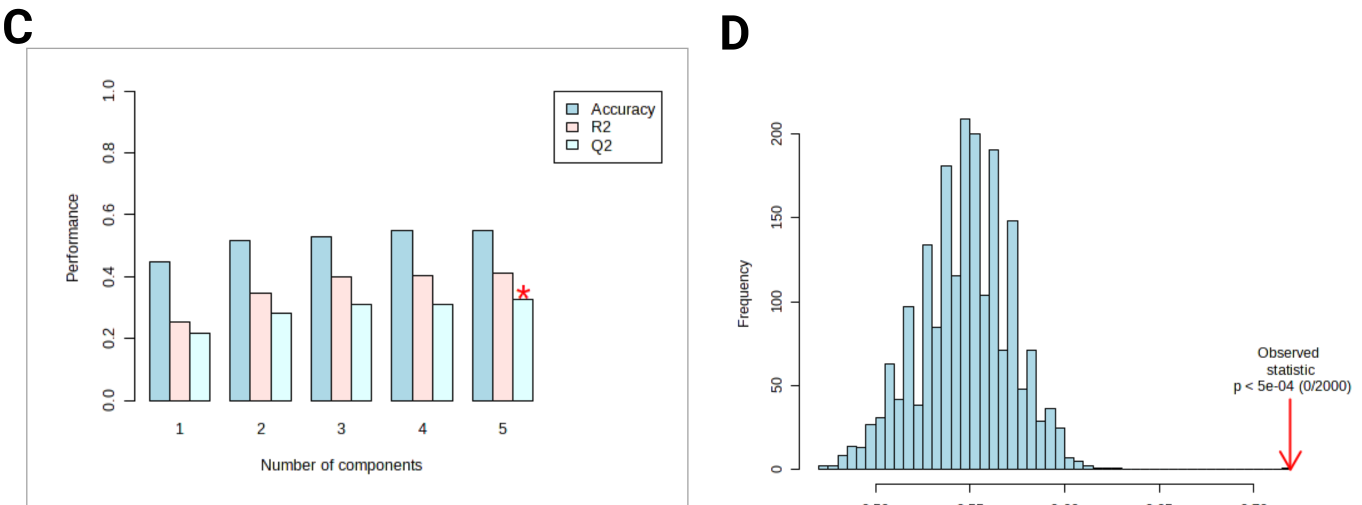

PLS-DA cross-validation details					
**Measure**	**1 comps**	**2 comps**	**3 comps**	**4 comps**	**5 comps**
**Accuracy**	0.44838	0.51613	0.52956	0.5486	0.54907
**R2**	0.2525	0.34436	0.3996	0.40313	0.41131
**Q2**	0.21622	0.2818	0.3098	0.31156	0.32654

Figure S10 Graphical results of the multivariate statistics for comparison 2 (all groups by sex). **A**: PCA scores plot (PC1 vs PC2). The lack of separation when the samples are grouped by sex indicates a larger source of variation in the data (such as the HIV status and ART regimen). **B**: PLS-DA scores plot. As the male and female children of each group cluster together, the HIV status and ART regimen has more dominant effects than sex on the plasma metabolome. **C**: PLS-DA five-fold cross-validation results. The model did not perform well, likely due to the decrease in sample size for each group as a consequence of stratification. This model was not used for marker selection in the sex comparisons. **D**: PLS-DA permutation testing results. The test statistic was prediction accuracy during testing, performed with 2000 permutations. The observed statistic was p<0,0005 (0/2000 permutations). This implies that the fitted model is unlikely to have occurred by random chance, although p-value estimations with this method may not be accurate with smaller sample sizes. (Related to Fig 4).

Data S4: Comparison 3 (EFV-LTWC Metabotypes)

Age and sex-matching of UCs to compare to EFV-LTWC (Related to Fig 5).

- This was done using the R package "MatchIt", in the following way:

m.out <- matchit(as.factor(group) ~ age, data = mydf, method = "nearest", exact = ~sex, distance = "euclidean")matched_samples <- match.data(m.out)

The credit for this code is due to an answer on StackOverflow:
https://stackoverflow.com/a/73963544

- **There were no differences in the age and the distribution of sex in the groups after the selection of matching UCs:**

Table S6 Summary of the statistical comparison of sex after matching UCs to EFV-LTWC

Contingency table for sex				Chi-square test	
	MT-A	MT-B	UC		
**F**	8	19	27	**Chi-square, df**	0,6807, 2
**M**	9	13	22	**p-value**	0,7115
**Total**	17	32	49		

Table S7 Kruskal-Wallis with Dunn's test as post-hoc for age (age and sex matched UCs vs metabotypes)

Dunn's multiple comparisons test	Mean rank diff,	Significant?	Summary	Adjusted P Value	
MT-A vs. MT-B	-0.6535	No	ns	>0,9999	A-B
MT-A vs. UC	-12.16	No	ns	0.3859	A-C
MT-B vs. UC	-11.51	No	ns	0.2249	B-C

Test details	Mean rank 1	Mean rank 2	Mean rank diff,	n1	n2
MT-A vs. MT-B	43.21	43.86	-0.6535	17	32
MT-A vs. UC	43.21	55.37	-12.16	17	49
MT-B vs. UC	43.86	55.37	-11.51	32	49

Comparison 3 (EFV-LTWC Metabotypes) (Related to Fig 5).

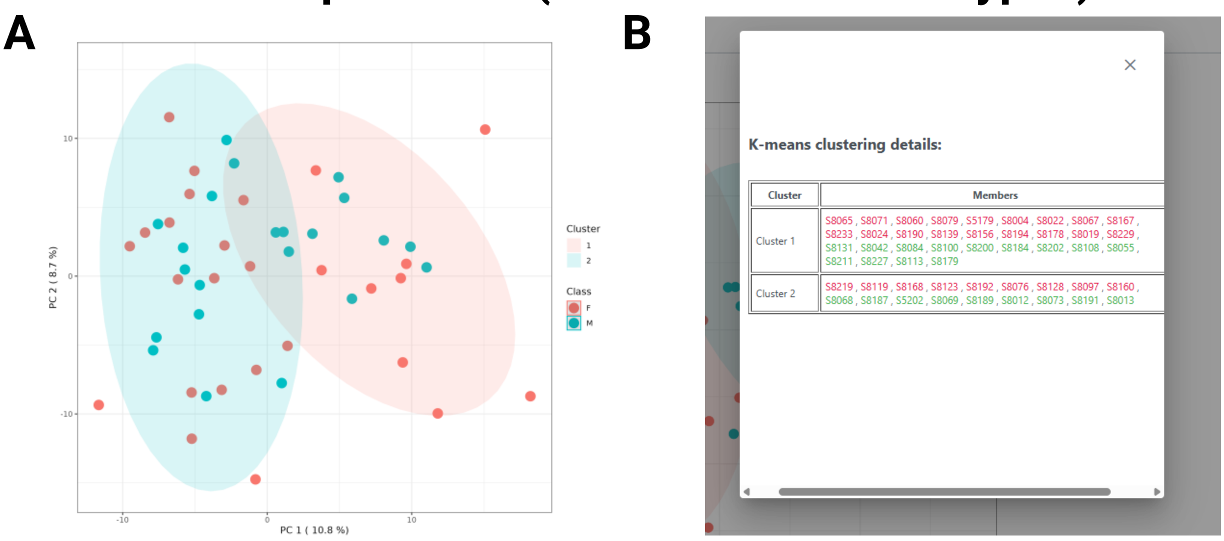

Figure S11 K-means clustering results. The cluster number was specified as two, to determine if this clustering algorithm would yield the same two sample clusters as found by HCA. The input data was grouped by sex as an arbitrary grouping variable, as all these participants were CLWH on an EFV-based ART regimen. **A:** A PCA scores plot of the samples as clustered by K-means clustering. Here, the colored ellipses represent clusters, while the color of the dots represents the sex (red: female, blue: male). **B:** A screenshot of the clustering results as output by MetaboAnalyst (there is no downloadable format for these results on this platform). Only one participant was clustered differently by K-means and HCA (S8123). See Table SX below.

Table S8 Cross-referencing of the clustering assignments by HCA and KMC

SampleID	K-means cluster	Hierarchical Clustering cluster	SampleID	K-means cluster	Hierarchical Clustering cluster
S8065	Cluster 1	Metabotype B	S8219	Cluster 2	Metabotype A
S8071	Cluster 1	Metabotype B	S8119	Cluster 2	Metabotype A
S8060	Cluster 1	Metabotype B	S8168	Cluster 2	Metabotype A
S8079	Cluster 1	Metabotype B	S8123	Cluster 2	Metabotype B
S5179	Cluster 1	Metabotype B	S8192	Cluster 2	Metabotype A
S8004	Cluster 1	Metabotype B	S8076	Cluster 2	Metabotype A
S8022	Cluster 1	Metabotype B	S8128	Cluster 2	Metabotype A
S8067	Cluster 1	Metabotype B	S8097	Cluster 2	Metabotype A
S8167	Cluster 1	Metabotype B	S8160	Cluster 2	Metabotype A
S8233	Cluster 1	Metabotype B	S8068	Cluster 2	Metabotype A
S8024	Cluster 1	Metabotype B	S8187	Cluster 2	Metabotype A
S8190	Cluster 1	Metabotype B	S5202	Cluster 2	Metabotype A
S8139	Cluster 1	Metabotype B	S8069	Cluster 2	Metabotype A
S8156	Cluster 1	Metabotype B	S8189	Cluster 2	Metabotype A
S8194	Cluster 1	Metabotype B	S8012	Cluster 2	Metabotype A
S8178	Cluster 1	Metabotype B	S8073	Cluster 2	Metabotype A
S8019	Cluster 1	Metabotype B	S8191	Cluster 2	Metabotype A
S8229	Cluster 1	Metabotype B	S8013	Cluster 2	Metabotype A
S8131	Cluster 1	Metabotype B			
S8042	Cluster 1	Metabotype B			
S8084	Cluster 1	Metabotype B			
S8100	Cluster 1	Metabotype B			
S8200	Cluster 1	Metabotype B			
S8184	Cluster 1	Metabotype B			
S8202	Cluster 1	Metabotype B			
S8108	Cluster 1	Metabotype B			
S8055	Cluster 1	Metabotype B			
S8211	Cluster 1	Metabotype B			
S8227	Cluster 1	Metabotype B			
S8113	Cluster 1	Metabotype B			
S8179	Cluster 1	Metabotype B			

VL and CD4% score calculations (Related to Fig 5).

- This was done using the R package "DescTools", in the following way:
- All available data for VL over time and the CD4% over time for each participant was organized into a separate file. This code was then used to apply the function to calculate the area under the curve to each participant's dataset:

```
allAUC<-lapply(split, function(x) AUC(x[,2],x[,1],method=c("trapezoid"), na.rm = TRUE))
```

- The resulting variables were then statistically compared across the metabotypes using the WR test and were correlated with other clinal parameters and metabolites.

Table S9 Normalization of longitudinal VL AUC to calculate final longitudinal VL score

Sample ID	Metabotype	VL AUC	Total follow-up time (months)	VL score (AUC normalized to total follow-up time)
8191	MT-A	2595.08	93.90	27.64
8012	MT-A	6367.61	117.51	54.19
8013	MT-A	91.31	3.47	26.34
8068	MT-A	5463.62	105.64	51.72
8076	MT-A	4392.67	119.80	36.67
8097	MT-A	2882.70	94.56	30.49
8119	MT-A	3114.20	104.43	29.82
8168	MT-A	2926.75	101.15	28.94
8187	MT-A	2877.64	97.08	29.64
8189	MT-A	2452.46	94.59	25.93
8219	MT-A	4990.00	102.23	48.81
8073	MT-A	6145.87	98.92	62.13
8160	MT-A	1758.52	73.18	24.03
5202	MT-A	15102.97	123.11	122.67
8069	MT-A	6842.56	108.79	62.90
8128	MT-A	5878.02	63.93	91.94
8192	MT-A	19358.62	102.89	188.16
8019	MT-B	3447.95	111.77	30.85
8227	MT-B	5269.30	106.46	49.50
8200	MT-B	5937.05	95.44	62.21
8211	MT-B	103999.92	95.21	1092.29
5179	MT-B	3674.20	116.07	31.66
8004	MT-B	3790.95	131.05	28.93
8022	MT-B	4275.28	112.69	37.94
8042	MT-B	2385.51	111.02	21.49
8067	MT-B	3985.80	111.84	35.64
8084	MT-B	10218.48	115.21	88.69
8100	MT-B	1746.59	72.59	24.06
8108	MT-B	3034.38	101.97	29.76
8113	MT-B	4144.84	92.43	44.84
8123	MT-B	6969.70	104.20	66.89
8131	MT-B	168905.97	98.62	1712.64
8139	MT-B	3447.92	93.18	37.00
8178	MT-B	3593.43	100.59	35.72
8190	MT-B	7567.33	98.52	76.81
8202	MT-B	2815.74	104.30	27.00
8233	MT-B	4004.75	90.89	44.06
8065	MT-B	3517.31	95.97	36.65
8167	MT-B	2545.18	84.16	30.24
8184	MT-B	2314.75	91.05	25.42
8229	MT-B	5907.49	81.34	72.62
8024	MT-B	9502.00	122.03	77.86
8055	MT-B	4568.03	74.39	61.40
8060	MT-B	7938.48	100.75	78.79
8071	MT-B	6747.02	102.66	65.72
8079	MT-B	10375.28	103.41	100.33
8156	MT-B	6466.46	111.05	58.23
8179	MT-B	5812.15	98.30	59.13
8194	MT-B	1286.98	63.74	20.19

(Related to Fig 5).

Table S10 Normalization of longitudinal CD4% AUC to calculate final longitudinal CD4% score

Sample ID	Metabotype	CD4% AUC	Total follow-up time (months)	CD4% score (AUC normalized to total follow-up time)
8191	MT-A	4135.51	101.77	40.64
8012	MT-A	4558.27	128.13	35.58
8013	MT-A	4195.06	122.36	34.28
8068	MT-A	3174.45	112.23	28.29
8076	MT-A	3785.06	126.52	29.92
8097	MT-A	3940.75	107.02	36.82
8119	MT-A	3200.38	110.39	28.99
8168	MT-A	2902.50	114.39	25.37
8187	MT-A	3176.91	116.43	27.29
8189	MT-A	3439.67	111.77	30.77
8219	MT-A	3396.82	106.00	32.05
8073	MT-A	4002.29	108.07	37.04
8160	MT-A	2569.67	83.84	30.65
5202	MT-A	4045.80	129.11	31.33
8069	MT-A	2977.15	112.69	26.42
8128	MT-A	1709.79	82.52	20.72
8192	MT-A	3954.16	107.67	36.72
8019	MT-B	4118.51	119.41	34.49
8227	MT-B	4364.07	112.49	38.79
8200	MT-B	2924.66	103.70	28.20
8211	MT-B	4755.96	107.41	44.28
5179	MT-B	4928.40	127.87	38.54
8004	MT-B	4521.27	136.30	33.17
8022	MT-B	3893.95	120.52	32.31
8042	MT-B	3514.64	116.30	30.22
8067	MT-B	3921.57	116.33	33.71
8084	MT-B	4638.95	123.38	37.60
8100	MT-B	3700.64	107.70	34.36
8108	MT-B	4683.52	107.64	43.51
8113	MT-B	2881.25	106.46	27.06
8123	MT-B	3903.09	108.69	35.91
8131	MT-B	2254.90	102.82	21.93
8139	MT-B	4340.43	103.05	42.12
8178	MT-B	4070.76	107.02	38.04
8190	MT-B	4203.06	105.80	39.73
8202	MT-B	4306.06	110.26	39.05
8233	MT-B	3842.60	97.90	39.25
8065	MT-B	3701.47	100.69	36.76
8167	MT-B	3051.96	91.41	33.39
8184	MT-B	2809.59	102.30	27.47
8229	MT-B	3522.09	94.43	37.30
8024	MT-B	4740.20	129.15	36.70
8055	MT-B	3328.83	100.13	33.24
8060	MT-B	3758.78	110.30	34.08
8071	MT-B	4179.68	111.48	37.49
8079	MT-B	4050.62	109.11	37.12
8156	MT-B	3570.35	116.98	30.52
8179	MT-B	4320.56	101.51	42.56
8194	MT-B	3717.94	103.97	35.76

Comparison 3 (EFV-LTWC Metabotypes)

A

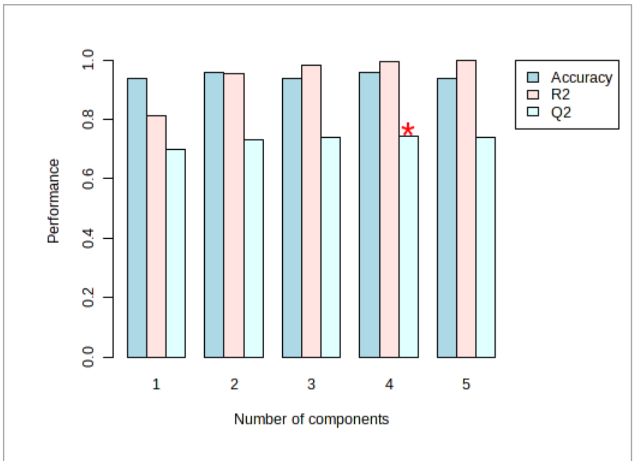

PLS-DA cross-validation details					
Measure	1 comps	2 comps	3 comps	4 comps	5 comps
Accuracy	0.93878	0.95918	0.93878	0.95918	0.93878
R2	0.81281	0.95509	0.98247	0.99292	0.9987
Q2	0.69828	0.73043	0.74077	0.74518	0.73783

C

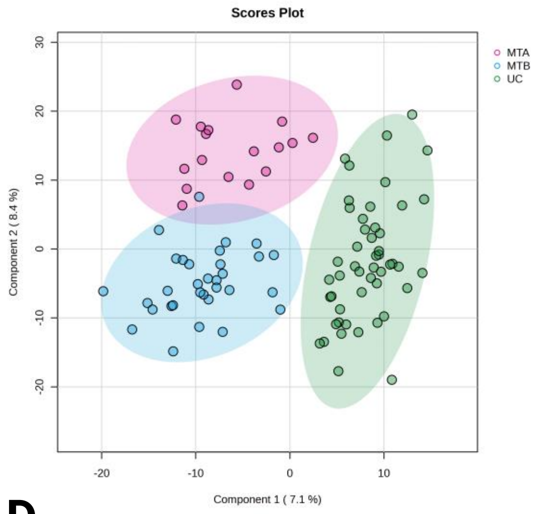

B

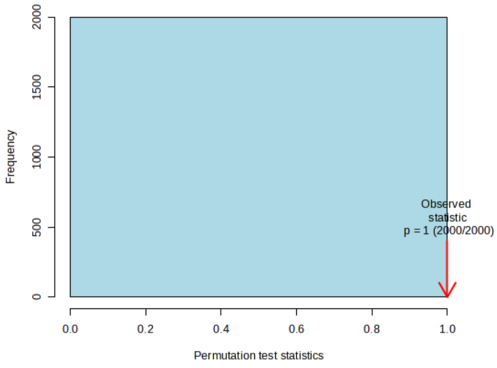

D

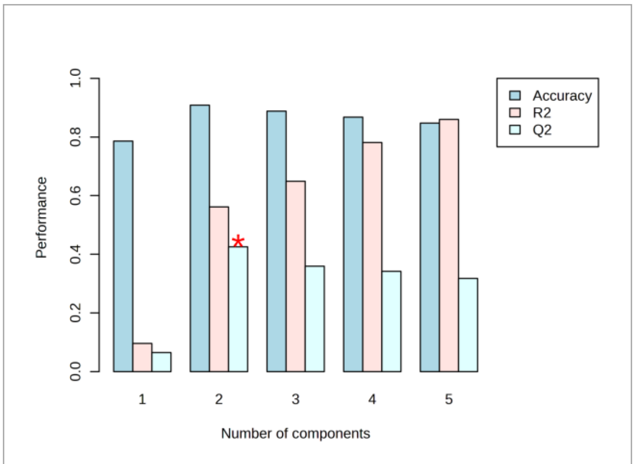

PLS-DA cross-validation details					
Measure	1 comps	2 comps	3 comps	4 comps	5 comps
Accuracy	0.78632	0.90895	0.88789	0.86789	0.84684
R2	0.09552	0.56129	0.64933	0.7809	0.85988
Q2	0.06491	0.42550	0.35905	0.34157	0.31704

E

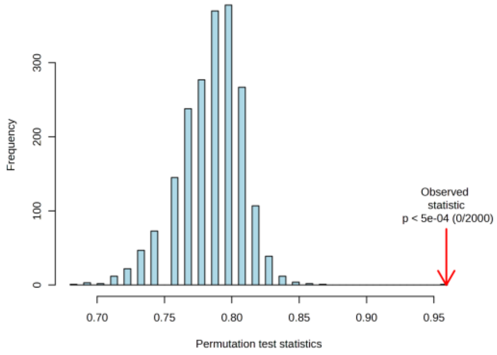

Figure S12 Graphical results of the multivariate statistics for comparison 3 (EFV-LTWC metabotypes). **A**: PLS-DA leave-one-out cross-validation results. The model performed well when four components were included. **B:** PLS-DA permutation testing results. The test statistic was prediction accuracy during testing, performed with 2000 permutations. The observed statistic was p<1 (2000/2000 permutations). This implies that the model could have been generated by random chance, however p-value estimations with this method are typically not accurate with smaller sample sizes. The PLS-DA was not used for marker selection for the metabotype comparison. **C:** PLS-DA scores plot. MT-A and MT-B separate from each other and the UCs. **D:** PLS-DA five-fold cross-validation results. The model performed best with two components, although its performance is not ideal, and it may be overfitted. **E:** PLS-DA permutation testing results. The test statistic was prediction accuracy during testing, performed with 2000 permutations. The observed statistic was p<0,0005 (0/2000 permutations). This implies that the fitted model is unlikely to have occurred by random chance. (Related to Fig 5).

Assessing the influence of age at ART initiation on the MT-A and MT-B divergence (Related to Fig 5).

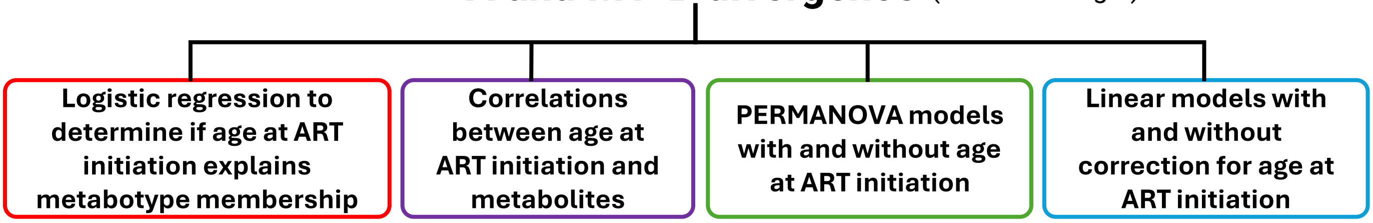

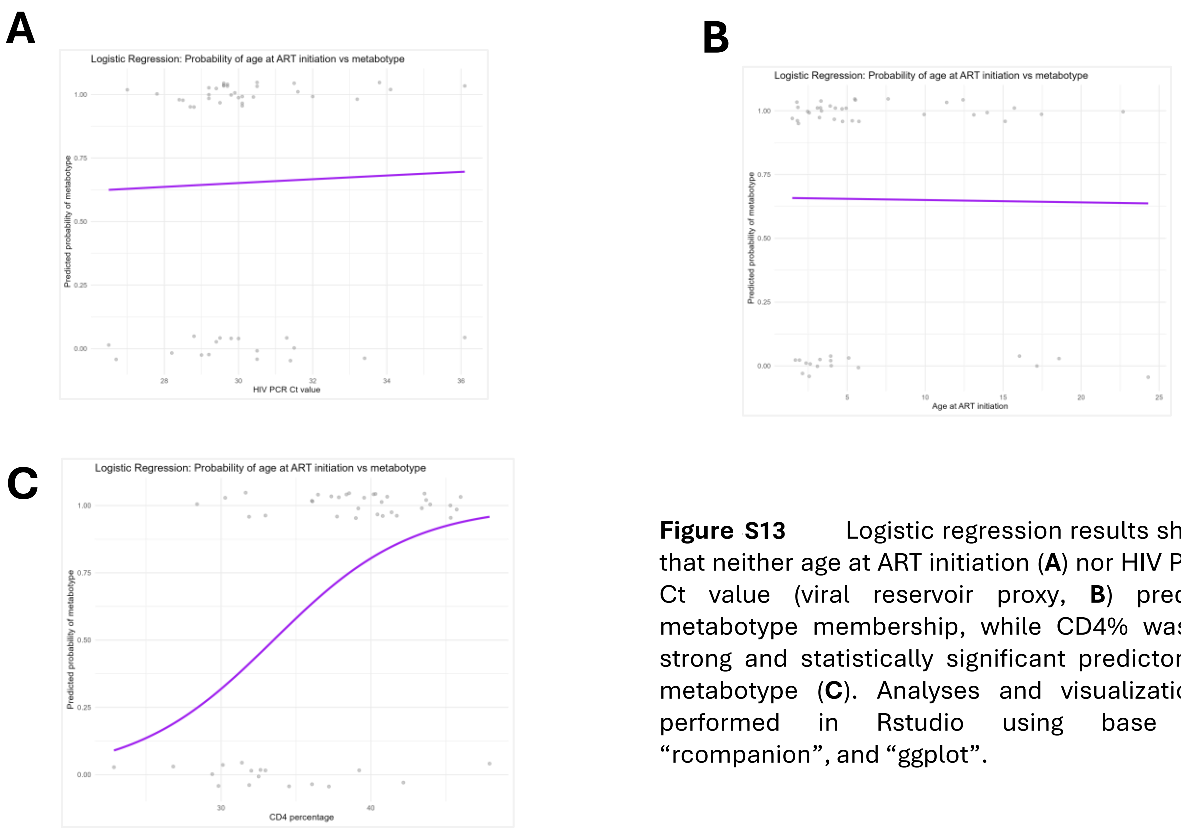

Figure S13 Logistic regression results show that neither age at ART initiation (**A**) nor HIV PCR Ct value (viral reservoir proxy, **B**) predict metabotype membership, while CD4% was a strong and statistically significant predictor of metabotype (**C**). Analyses and visualizations performed in Rstudio using base R, "rcompanion", and "ggplot".

Table S11 Logistic regression results for individual models assessing the ability of age at ART initiation, HIV reservoir size, and CD4 percentage to predict metabotype membership

	Age at ART initiation	Viral reservoir (HIV PCR Ct)	CD4 percentage
**Coefficients**			
**Intercept**	0.66 (p=0.149)	0.370 (p=0.149)	-7.28 (p=0,005)
**Slope**	-0.004 (p=0.935)	0.033 (p=0.827)	0.217 (p=0,0025)
**Odds ratio**	0.996	1.030	1.240
**Model fit**			
**Null deviance**	63.262	63.26	63.260
**Residual deviance**	63.255	63.21	50.870
**Akaike's Information Criterion (AIC)**	67.255	67.21	54.870
**Pseudo R^2**			
**Nagelkerke's R^2**	0.000186	0.00136	0.308246

To further assess whether age at ART initiation may contribute to the metabotype divergence, linear models with and without correction for age at ART initiation was performed (MetaboAnalyst 6.0), as well as individual PERMANOVA models assessing the individual contribution of grouping (metabotype) and age at ART initiation on the metabolic variance, in addition to another PERMANOVA assessing the marginal effects of these two variables (in Rstudio using the 'vegan' package[1]).

PERMANOVA RESULTS (Related to Fig 5).

Table S12 Effect of Group PERMANOVA

	Degrees of freedom	Sum of squares	R^2	F-statistic	Permutation (n=999) p-value
**Group**	1	0.3243	0.08603	4.424	**0.001**
**Residual**	47	3.4455	0.91397		
**Total**	48	3.7699	1.00000		

Table S13 Effect of Age at ART start PERMANOVA

	Degrees of freedom	Sum of squares	R^2	F-statistic	Permutation (n=999) p-value
**Group**	1	0.0555	0.01473	0.7026	0.966
**Residual**	47	3.7143	0.98527		
**Total**	48	3.7699	1.00000		

Table S14 Marginal effects of Group and Age at ART start PERMANOVA

	Degrees of freedom	Sum of squares	R^2	F-statistic	Permutation (n=999) p-value
**Group**	1	0.3238	0.08589	4.3929	**0.001**
**Age at ART initiation**	1	0.0550	0.01459	0.7461	0.934
**Residual**	46	3.3906	0.89938		
**Total**	48	3.7699	1.00000		

The low R^2 values and high permutation p-values associated with age at ART initiation in each case suggest the age at ART initiation does not contribute appreciably to the metabolic variation between metabotypes.

[1] Oksanen J, Simpson G, Blanchet F, Kindt R, Legendre P, Minchin P, O'Hara R, Solymos P, Stevens M, Szoecs E, Wagner H, Barbour M, Bedward M, Bolker B, Borcard D, Borman T, Carvalho G, Chirico M, De Caceres M, Durand S, Evangelista H, FitzJohn R, Friendly M, Furneaux B, Hannigan G, Hill M, Lahti L, Martino C, McGlinn D, Ouellette M, Ribeiro Cunha E, Smith T, Stier A, Ter Braak C, Weedon J (2025). _vegan: Community Ecology Package_. R package version 2.7-1,<https://CRAN.R-project.org/package=vegan>.

LINEAR MODELS WITH AND WITHOUT CORRECTION FOR AGE AT ART INITIATION (Related to Fig 5).

Table S15 Results of linear models contrasting MT-A and MT-B with and without correction for age at ART initiation

	Without adjustment			With adjustment for age at ART initiation		
**Metabolite**	**p-value**	**Adj. p-value**	**p-value ranking**	**p-value**	**Adj. p-value**	**p-value ranking**
glutamine conjugate of C7H12O2*	2.31E-11	2.11E-08	1	2.1846E-11	1.999E-08	1
docosapentaenoate (n3 DPA; 22:5n3)	7.14E-10	2.91E-07	2	5.1474E-10	2.355E-07	2
10-nonadecenoate (19:1n9)	9.55E-10	2.91E-07	3	1.3428E-09	4.096E-07	3
10-heptadecenoate (17:1n7)	2.28E-09	3.54E-07	4	3.115E-09	4.316E-07	6
(R)-3-hydroxybutyrylcarnitine	2.33E-09	3.54E-07	5	2.3636E-09	4.316E-07	5
(14 or 15)-methylpalmitate (a17:0 or i17:0)	2.41E-09	3.54E-07	6	2.3263E-09	4.316E-07	4
3-hydroxyoleoylcarnitine	2.77E-09	3.54E-07	7	3.7735E-09	4.316E-07	8
X-21353	3.10E-09	3.54E-07	8	4.2603E-09	4.331E-07	9
docosahexaenoate (DHA; 22:6n3)	3.83E-09	3.90E-07	9	3.7578E-09	4.316E-07	7
dodecadienoate (12:2)*	4.32E-09	3.95E-07	10	5.8714E-09	4.884E-07	11
palmitoleoylcarnitine (C16:1)*	4.91E-09	4.07E-07	11	4.7532E-09	4.349E-07	10
margarate (17:0)	5.34E-09	4.07E-07	12	7.2309E-09	5.514E-07	12
palmitoleate (16:1n7)	7.48E-09	4.86E-07	13	9.8711E-09	6.371E-07	14
myristoleate (14:1n5)	7.96E-09	4.86E-07	14	8.9658E-09	6.311E-07	13
3-hydroxyoctanoylcarnitine (1)	7.97E-09	4.86E-07	15	1.0444E-08	6.371E-07	15
dihomo-linolenate (20:3n3 or n6)	8.58E-09	4.91E-07	16	1.1291E-08	6.457E-07	16
3-hydroxydecanoylcarnitine	1.06E-08	5.68E-07	17	1.4213E-08	7.65E-07	17
nonadecanoate (19:0)	1.30E-08	6.61E-07	18	1.7233E-08	8.76E-07	18
eicosenoate (20:1)	1.44E-08	6.96E-07	19	1.919E-08	9.018E-07	19
X-26107	1.58E-08	7.21E-07	20	2.0698E-08	9.018E-07	21
hexanoylcarnitine (C6)	1.71E-08	7.44E-07	21	2.0328E-08	9.018E-07	20
adrenate (22:4n6)	2.70E-08	1.09E-06	22	3.5281E-08	1.345E-06	24
myristoleoylcarnitine (C14:1)*	2.85E-08	1.09E-06	23	3.7337E-08	1.367E-06	25
3-hydroxybutyrate (BHBA)	2.87E-08	1.09E-06	24	3.5213E-08	1.345E-06	23
eicosapentaenoate (EPA; 20:5n3)	3.26E-08	1.15E-06	25	2.6115E-08	1.086E-06	22
3-hydroxylaurate	3.27E-08	1.15E-06	26	4.1422E-08	1.458E-06	26
oleate/vaccenate (18:1)	5.11E-08	1.65E-06	27	6.4881E-08	2.043E-06	27
stearate (18:0)	5.22E-08	1.65E-06	28	6.6179E-08	2.043E-06	28
X-17335	5.23E-08	1.65E-06	29	6.6188E-08	2.043E-06	29
octanoylcarnitine (C8)	6.26E-08	1.91E-06	30	6.698E-08	2.043E-06	30

- Without correction for age at ART initiation, 208 metabolites were significantly different between MT-A and MT-B based on a linear model. In a model with correction for age at ART initiation, 206 of the same metabolites were significant between the two groups, while 3-hydroxyhippurate and 3-hydroxypyridine sulfate (the two least significant metabolites in the model without correction) were no longer significant and 4-acetylphenol sulfate was additionally significant.
- Table S2.10.3 shows that the same set of metabolites were among the top 30 most significantly different metabolites between MT-A and MT-B in linear models, with or without correction for age at ART initiation. The blue highlights indicate where the ranking of a metabolite (based on adjusted p-values) changed based on whether or not covariate correction was applied.
- Thus the only difference between the results of the models with and without correction lies in three metabolites gaining or losing significance, and the ranking of metabolites based on the adjusted p-values. **This confirms the PERMANOVA results — the age at ART initiation does not significantly modify the observed metabolic differences between MT-A and MT-B.**

Data S5: Comparison 4

(the effect of HIV irrespective of the treatment regimen)

APPROACH 1: GROUP COMPARISONS

- The two-by-two comparisons of the KWD related to comparison 2 (UC vs EFV-CLWH vs LPV-CLWH):

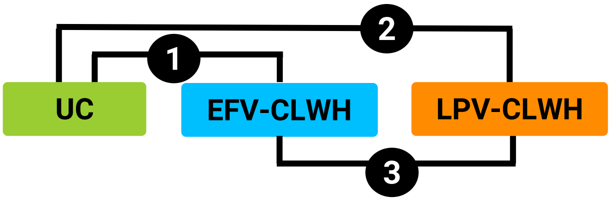

- These resulted in three sets of metabolites. Metabolites significantly different between:

1 UC vs EFV-CLWH

2 UC vs LPV-CLWH

3 EFV-CLWH vs LPV-CLWH

- Many metabolites appear in more than one set, but the direction and magnitude of differences varied. A Venn-diagram approach was used to identify the overlaps between these sets of metabolites. These overlapping metabolites were those with the highest potential of being related to an HIV rather than treatment related effect.

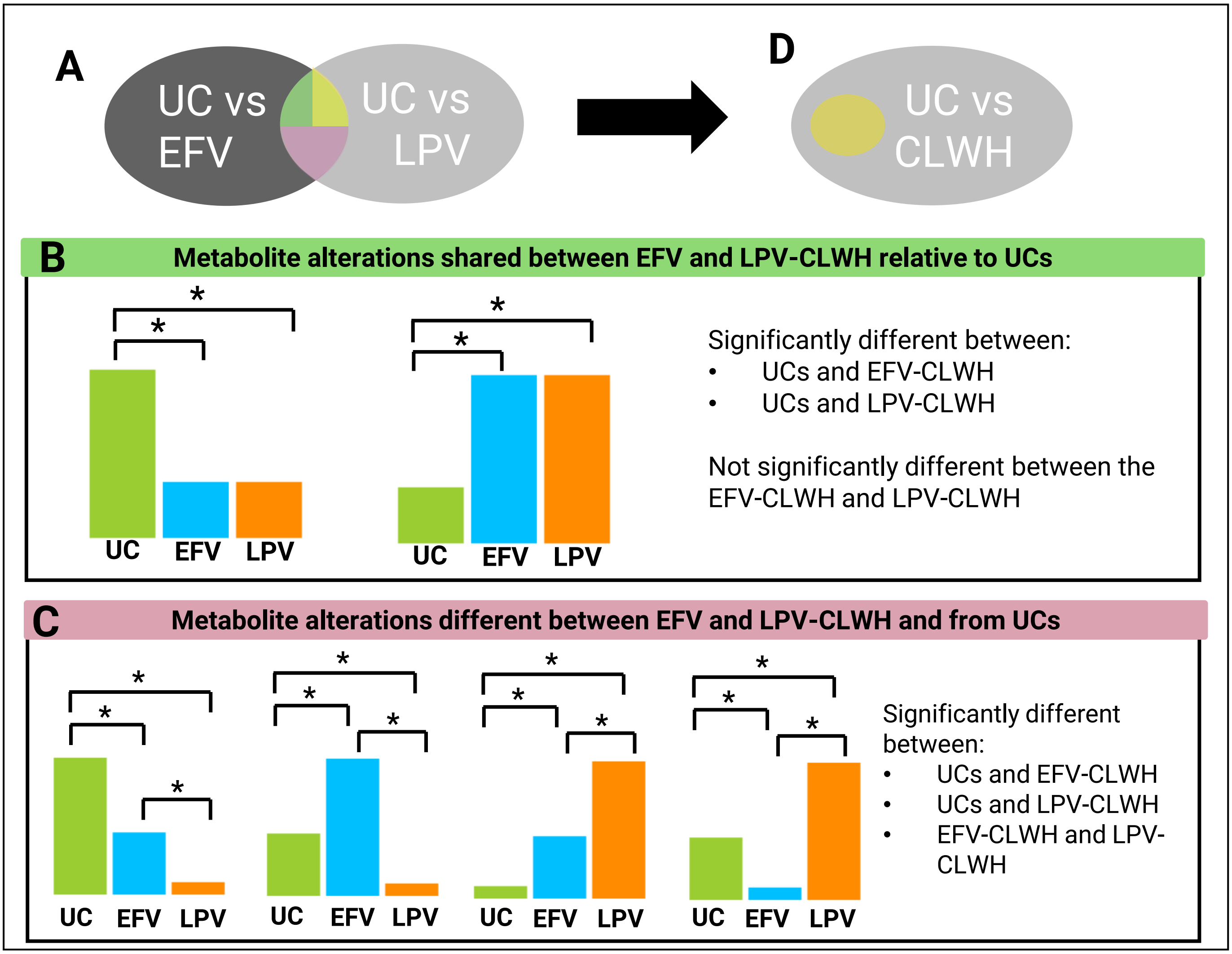

Figure S14 Patterns of metabolic differences between UCs, EFV-CLWH, and LPV-CLWH as well as those between UCs and CLWH ırrespective of treatment regimen. A: A Venn diagram illustrating these patterns. The set of metabolites indicated in yellow in the Venn diagram were those relevant to the effect of HIV irrespective of treatment regimen. These proportions are not accurate and are only for conceptual illustration. The metabolite set in pink were different from the UCs in both treatment groups but were also significantly different between the treatment groups and would therefore not be expected to be a consistent finding irrespective of ART regimen. **B:** Metabolite alterations shared between EFV and LPV-CLWH relative to UCs in the KWD (green plus yellow in Venn diagram). **C:** Metabolite alterations different between EFV and LPV-CLWH and from UCs (pink in Venn diagram). **D:** Metabolite alterations shared between EFV and LPV-CLWH relative to UCs and which were significantly different in CLWH (unstratified) compared to UCs (yellow). Only two metabolites fulfilled these conditions. (Related to Fig 6). Asterisks (*) indicate arbitrary statistical significance to facilitate this conceptual explanation.

APPROACH 2: Classification methods (UC vs CLWH)

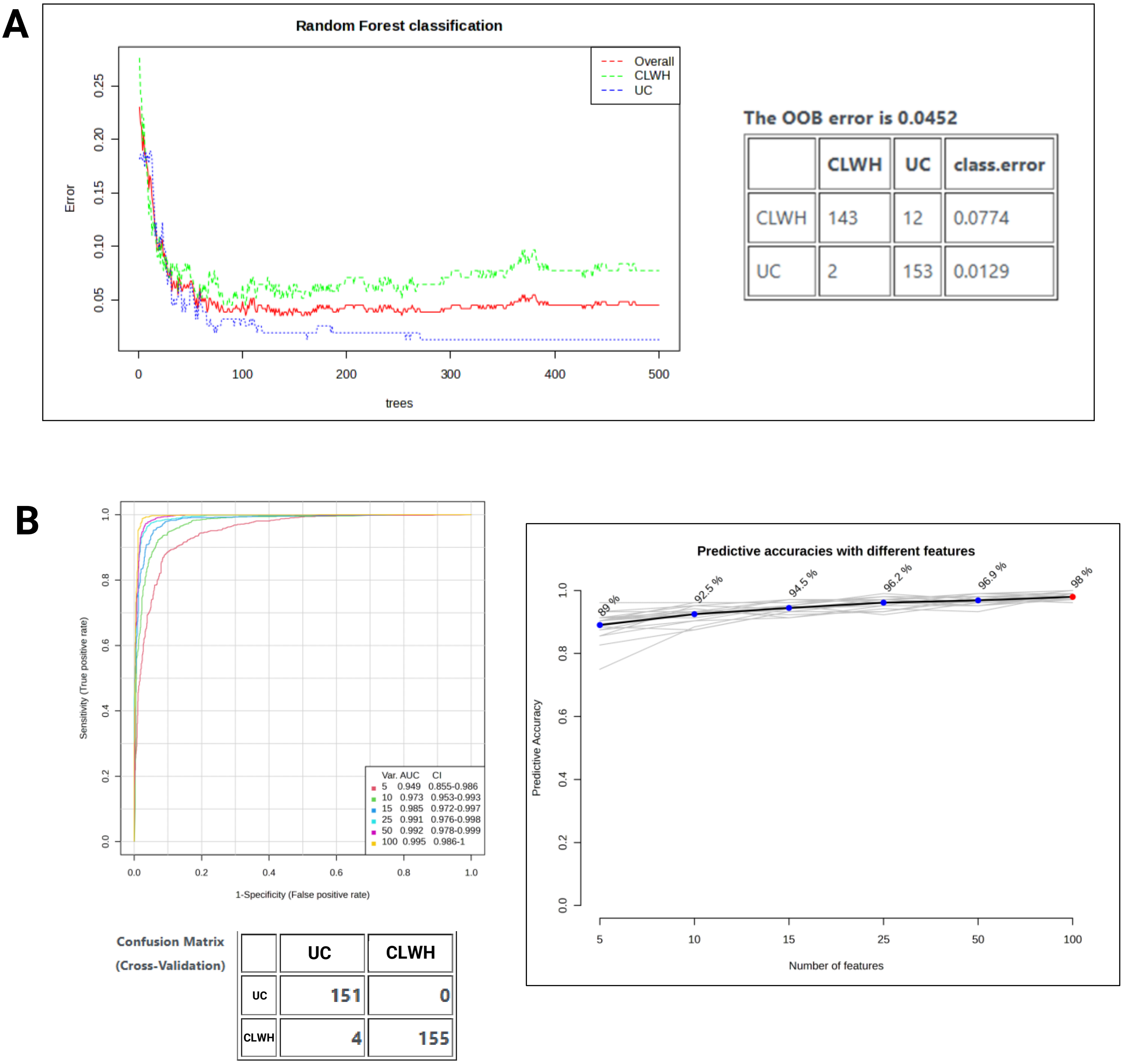

	CLWH	UC	class.error
CLWH	143	12	0.0774
UC	2	153	0.0129

	UC	CLWH
UC	151	0
CLWH	4	155

Figure S15 Graphical results of the classification methods applied to identify metabolic alterations common to CLWH irrespective of ART regimen. **A:** Random forests classification performance graph and the associated confusion matrix and out-of-bag error (0,0452), which shows good model performance. This analysis was performed using 500 trees and seven predictors. Based on this confusion matrix, CLWH are more likely to be classified as UCs than UCs are of being classified as CLWH. Thus, there is a higher level of false negatives than false positives. **B:** Results and validation of the multivariate ROC analysis performed using the linear support vector machine algorithm as classification and feature ranking method for distinguishing CLWH from UCs. This model also misclassified UCs rather than CLWH. (Related to Fig 6).

Table S16 Previously published biological associations of unannotated metabolites with high rankings in classification methods (UC vs CLWH). (Related to Fig 6).
This is not an exhaustive collection of all reports of these metabolites.

**X-21471**		
Associated genes		
Gene	Gene name	Context & Reference
MTERF1	Mitochondrial transcription termination factor 1	Metabolic syndrome in Finnish men[1]
ABCC2	ATP binding cassette subfamily C member 2	
DNMBP	Dynamin binding protein	
SLCO1B1	Solute carrier organic anion transporter family member 1B1	
LINC01595	Long intergenic non-protein coding RNA 1595	
SULT2A1	Sulfotransferase family 2A member 1	
Other published reports		
Ye et al., 2024	Peripheral arteriosclerosis[2]	
**X-21467**		
Associated genes		
**Gene**	**Gene name**	**Context & Reference**
ABCC2	ATP binding cassette subfamily C member 2	Genomic regions variants associated with circulating levels metabolites in Europeans[3]
SLCO1B1	Solute carrier organic anion transporter family member 1B1	
SLCO1B3	Solute carrier organic anion transporter family member 1B3	
SLCO1C1	Solute carrier organic anion transporter family member 1C1	
Other published reports		
Vike et al., 2022	Exposure to collegiate football[4]	
Chen et al., 2025	Associations with arterial fibrillation and stroke[5]	
Peng et al., 2025	Female pattern hair loss[6]	

References:

1. Yin X, Chan LS, Bose D, Jackson AU, Vandehaar P, Locke AE, et al. Genome-wide association studies of metabolites in Finnish men identify disease-relevant loci. Nature Communications. 2022;**13**(1).

2. Ye Q, Zhou Y, Xu K, Jiang Z. Causality of blood metabolites and metabolic pathways on peripheral arteriosclerosis: a Mendelian randomization study. Frontiers in Nutrition. 2024;**11**.

3. Hysi PG, Mangino M, Christofidou P, Falchi M, Karoly ED, Mohney RP, et al. Metabolome Genome-Wide Association Study Identifies 74 Novel Genomic Regions Influencing Plasma Metabolites Levels. Metabolites. 2022;**12**(1):61.

4. Vike NL, Bari S, Stetsiv K, Talavage TM, Nauman EA, Papa L, et al. Metabolomic response to collegiate football participation: Pre- and Post-season analysis. Scientific Reports. 2022;**12**(1):3091.

5. Chen J-L, Lu X-Y, Chen D-Z, Chen Y. Lipid metabolism-associated metabolites on cardiovascular diseases: a two-sample Mendelian randomized study. Frontiers in Cardiovascular Medicine. 2025;**12**.

6. Peng L, Zhao X, Shen L, Zhang L, Han Y, Li L, et al. Causal Relationship Between Blood Metabolomics and Female Pattern Hair Loss: A Bidirectional Mendelian Randomization Study. Clinical, Cosmetic and Investigational Dermatology. 2025;**18**:383-92.

Data S6:

Comparing LPV-CLWH who had ever been on EFV before to those who had never been on EFV

(Relates to the 'Setting the stage' section of Results)

A

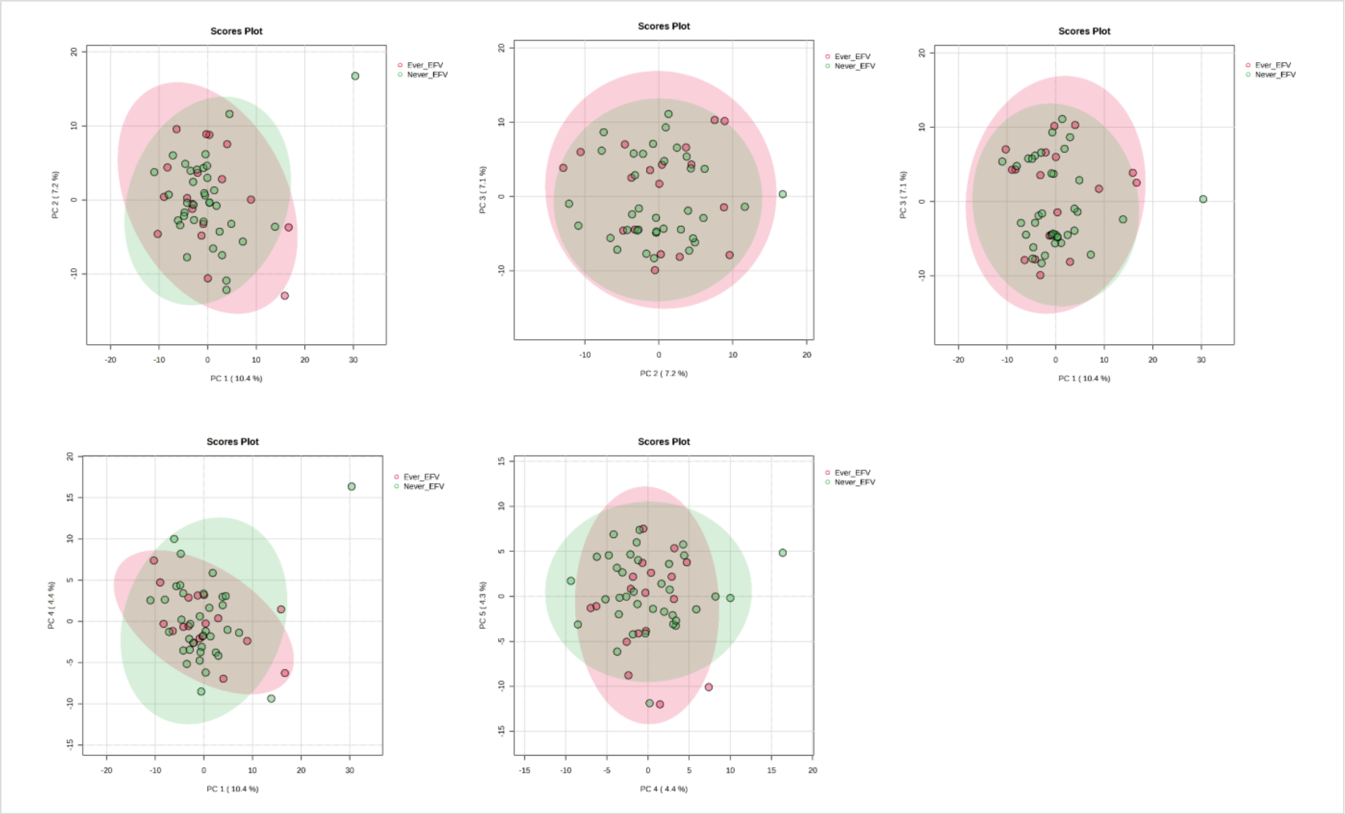

B

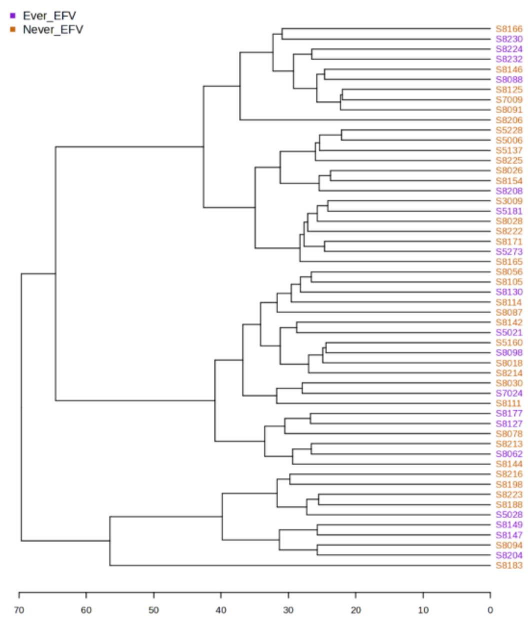

C

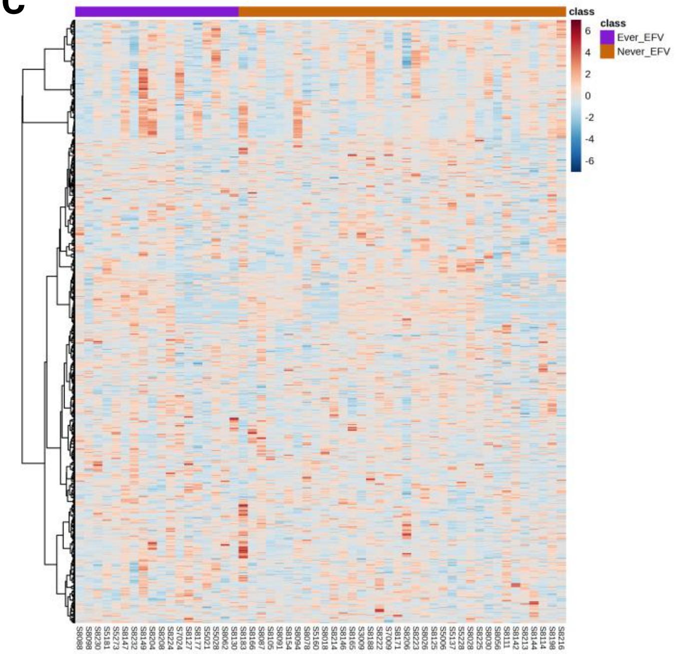

Figure S16 **A:** PCA scores plots, of different principal components plotted against one another, to show that there is very little metabolic difference between the LPV-CLWH who had ever or never been on EFV. **B**: A dendogram and **C**: a unclustered heatmap, also showing that there is no clear metabolic differences between the ever on EFV and the never on EFV groups.

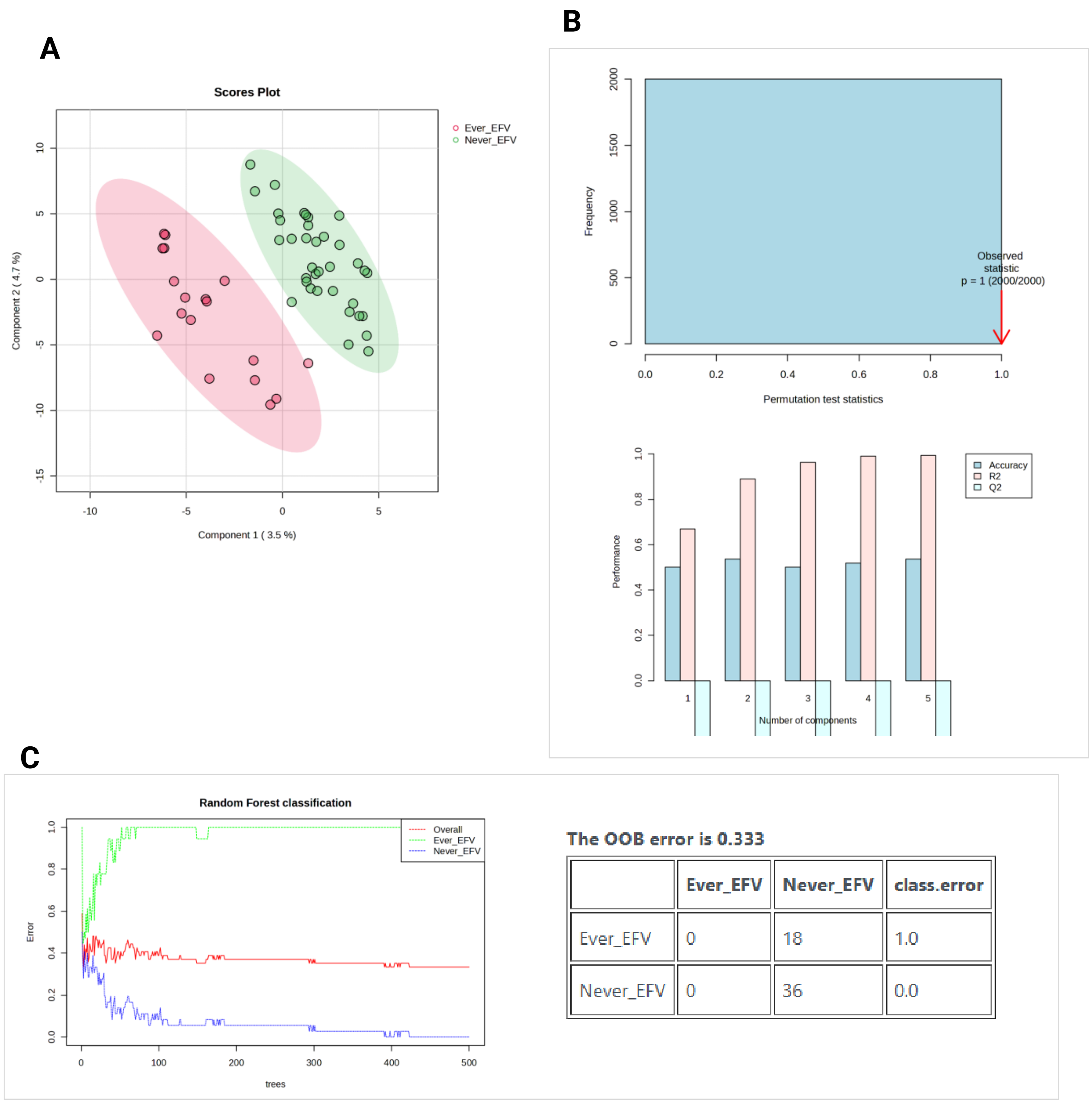

Figure S17 **A and B:** The PLS-DA scores plot and its accompanying validation metrics. This model could not be validated, it failed on both cross-validation and permutation testing. **C:** The random forest analysis failed to classify those who had ever been on EFV as a separate group—all were classified with those who had never been on EFV. This implies that the current ART regimen's metabolic effects are more prominent than a previous regimen (at least as measured here, with short term use of the alternative regimen and after approximately X years since the switch). This is not unexpected, given the immediate response of the metabolic system to physiological changes. This does not, however, preclude that a particular regimen may have long term metabolic effects, especially if used for a longer period of time. Whether these effects would still be observable in the metabolic profile, or would rather (more likely) be observed as higher level physiological changes or damage (such as neurological damage), needs to be investigated. (Relates to the 'Setting the stage' section of Results)

Table S17 Metabolites with significant fold change in the LPV-ever EFV vs LPV-never EFV comparison (Relates to the 'Setting the stage' section of Results).

CHEMID	Fold change	Metabolite	Sub-pathway
1E+09	0.1374	X-12731	Unannotated
1E+09	0.26184	X-23157	Unannotated
1E+09	0.33166	X-24475	Unannotated
1E+09	0.36838	X-24522	Unannotated
1E+09	0.39017	X-23662	Unannotated
1E+09	0.39113	X-12701	Unannotated
1E+09	0.4138	X-11852	Unannotated
1E+09	0.42306	X-25279	Unannotated
1E+09	0.47892	X-12007	Unannotated
1E+09	3.289	X-24571	Unannotated
1E+09	3.3459	X-12740	Unannotated
1E+08	0.24156	vanilloylglycine	Food Component/Plant
1E+08	0.45817	umbelliferone sulfate	Food Component/Plant
1E+08	2.2947	1,6-anhydroglucose	Food Component/Plant
1E+08	2.969	2-piperidinone	Food Component/Plant
1E+08	3.3667	daidzein sulfate (2)	Food Component/Plant
1E+08	3.7602	eugenol sulfate	Food Component/Plant
1E+08	3.9199	genistein sulfate*	Food Component/Plant
1869	0.14504	2-hydroxyhippurate (salicylurate)	Benzoate Metabolism
1E+08	0.4657	propyl 4-hydroxybenzoate sulfate	Benzoate Metabolism
1E+08	0.49952	methyl-4-hydroxybenzoate sulfate	Benzoate Metabolism
1E+08	0.11518	hyocholate	Secondary Bile Acid Metabolism
1E+08	3.5398	glycohyocholate	Secondary Bile Acid Metabolism
1E+08	3.676	taurohyocholate*	Secondary Bile Acid Metabolism
1E+08	4.3617	tauro-beta-muricholate	Primary Bile Acid Metabolism
1E+08	4.7498	glyco-beta-muricholate**	Primary Bile Acid Metabolism
1E+08	0.49225	androstenediol (3alpha, 17alpha) monosulfate (2)	Androgenic Steroids
1E+08	0.24049	5-hydroxy-2-methylpyridine sulfate	Chemical
1E+08	0.49897	oleoyl-linolenoyl-glycerol (18:1/18:3) [2]*	Diacylglycerol
1E+08	2.1734	14-HDoHE/17-HDoHE	Docosanoid
501	0.10297	salicylate	Drug - Topical Agents
1E+08	2.0217	dodecanedioate (C12-DC)	Fatty Acid, Dicarboxylate
878	0.44389	fructose	Fructose, Mannose and Galactose Metabolism
1025	2.2541	pipecolate	Lysine Metabolism
181	2.1034	laurate (12:0)	Medium Chain Fatty Acid
1E+08	2.5295	glucuronide of C12H22O4 (1)*	Partially Characterized Molecules
1E+08	4.5494	N-acetylkynurenine (2)	Tryptophan Metabolism
1E+08	0.49809	4-hydroxyphenylacetoylcarnitine	Tyrosine Metabolism
1E+08	0.27286	homocitrulline	Urea cycle; Arginine and Proline Metabolism
849	0.30889	caffeine	Xanthine Metabolism

Table S18 Metabolites with significant raw p-values (but not FDR) in the LPV-ever EFV vs LPV-never EFV comparison (Relates to the 'Setting the stage' section of Results).

CHEMID	p-value	FDR	FC	Metabolite	Median Ever EFV	Median Never EFV	Linegraph
100000963	0.003272	0.99411	0.27286	homocitrulline	0.0731	0.8503	
100000939	0.036598	0.99411	2.2947	1,6-anhydroglucose	0.8235	0.1899	
100006378	0.008969	0.99411	4.5494	N-acetylkynurenine (2)	0.4999	0.0638	
999923587	0.000988	0.99411		X-23587	0.9531	1.5612	
100009015	0.005635	0.99411		1-(1-enyl-stearoyl)-2-docosahexaenoyl-GPC (P-18:0/22:6)*	0.9519	1.2933	
100009037	0.007151	0.99411		1-margaroyl-2-linoleoyl-GPC (17:0/18:2)*	1.0049	1.1548	
100001619	0.009392	0.99411		glycerophosphoglycerol	1.5554	0.9423	
100004414	0.010093	0.99411		2-hydroxyphytanate*	0.6696	1.1818	
100009130	0.015622	0.99411		1-oleoyl-2-docosahexaenoyl-GPC (18:1/22:6)*	1.0017	1.1407	
100020225	0.015622	0.99411		1-(1-enyl-oleoyl)-2-docosahexaenoyl-GPC (P-18:1/22:6)*	0.9135	1.1589	
999913553	0.018377	0.99411		X-13553	1.1303	0.7351	
391	0.021324	0.99411		citrulline	0.9954	0.8269	
999911315	0.021324	0.99411		X-11315	1.0258	0.9179	
999923639	0.023582	0.99411		X-23639	1.1484	0.9762	
999925810	0.023983	0.99411		X-25810	1.3004	1.5874	
339	0.027348	0.99411		glutarate (C5-DC)	0.9758	0.6788	
100009003	0.028993	0.99411		1-(1-enyl-oleoyl)-2-linoleoyl-GPE (P-18:1/18:2)*	0.6164	0.9642	
100006360	0.030131	0.99411		dopamine 4-sulfate	1.3538	0.9957	
100008916	0.030131	0.99411		1-stearoyl-2-docosahexaenoyl-GPC (18:0/22:6)	0.9788	1.1334	
100001950	0.031609	0.99411		bilirubin (E,E)*	1.4789	1.0388	
100000773	0.032537	0.99411		3-hydroxyoctanoate	1.1321	0.7873	
999924588	0.03326	0.99411		X-24588	1.3772	1.0122	
999915503	0.034749	0.99411		X-15503	1.1575	0.9908	
100001253	0.036415	0.99411		N-acetylglutamine	0.9749	0.8059	
100020371	0.038146	0.99411		N,N-dimethylalanine	0.5744	1.2471	
999912714	0.038626	0.99411		X-12714	0.2198	1.1478	
100015962	0.039946	0.99411		N,N,N-trimethyl-5-aminovalerate	1.1087	1.5846	
999921736	0.039946	0.99411		X-21736	1.3726	0.7702	
999911407	0.041394	0.99411		X-11407	0.0873	0.8271	
100001212	0.043709	0.99411		guanidinosuccinate	0.0839	0.8948	
1083	0.045771	0.99411		N-acetylmethionine	1.1171	0.9642	
999921339	0.045771	0.99411		X-21339	0.9049	1.3075	
466	0.047505	0.99411		phytanate	1.0241	1.6301	
100001170	0.048032	0.99411		3-hydroxy-2-ethylpropionate	0.7248	1.0834	

Metadata comparisons between the ever and never on EFV LPV-CLWH groups

(Relates to the 'Setting the stage' section of Results)

A

Stunting

Approaching significance

P value	0.0510		
P value summary	ns		
One- or two-sided	Two-sided		
Statistically significant (P < 0.05)?	No		
**Data analyzed**	**Stunted**	**Not stunted**	**Total**
Never on EFV	3	28	31
Ever on EFV	6	11	17
Total	9	39	48
**Percentage of row total**	**Stunted**	**Not stunted**	
Never on EFV	9.68%	90.32%	
Ever on EFV	35.29%	64.71%	
**Percentage of column total**	**Stunted**	**Not stunted**	
Never on EFV	33.33%	71.79%	
Ever on EFV	66.67%	28.21%	
**Percentage of grand total**	**Stunted**	**Not stunted**	
Never on EFV	6.25%	58.33%	
Ever on EFV	12.50%	22.92%	

D

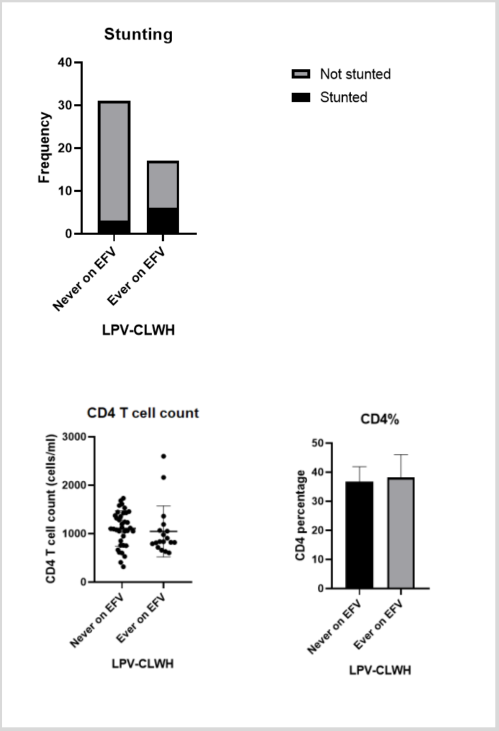

B

CD4 T cell count

Table Analyzed	CD4 count
Column B	Ever on EFV
vs.	vs.
Column A	Never on EFV
**Mann Whitney test**	Not significant
P value	0.1252
Exact or approximate P value?	Exact
P value summary	ns
Significantly different (P < 0.05)?	No
One- or two-tailed P value?	Two-tailed
Sum of ranks in column A,B	1074 , 411
Mann-Whitney U	240
**Difference between medians**	
Median of column A	1109, n=36
Median of column B	834.0, n=18
Difference: Actual	-274.5
Difference: Hodges-Lehmann	-217.0

C

CD4 T cell percentage

Table Analyzed	CD4%
Column B	Ever on EFV
vs.	vs.
Column A	Never on EFV
**Mann Whitney test**	Not significant
P value	0.6590
Exact or approximate P value?	Exact
P value summary	ns
Significantly different (P < 0.05)?	No
One- or two-tailed P value?	Two-tailed
Sum of ranks in column A,B	965.5 , 519.5
Mann-Whitney U	299.5
**Difference between medians**	
Median of column A	37.18, n=36
Median of column B	38.70, n=18
Difference: Actual	1.520
Difference: Hodges-Lehmann	0.7800

Figure S18 Results from the statistical comparison of stunting (considered stunted when height-for-age z-score<2) (**A**), CD4 T cell count (**B**), and CD4 T cell percentage (**C**) among the ever and never on EFV groups of LPV-CLWH, to determine if there is any indication that those who had ever been on EFV had worse immunological or growth outcomes. Plots showing the distribution of values for these three parameters are shown in **D**.

Metabotype analysis amongst LPV-LTWC

(Relates to Fig 5)

Table S19 Cross-referencing of the clustering assignments by HCA, KMC, and a self-organizing map (SOM)

Sample ID	HC	SOM	KMC
S8018	B	SOM1	KMC2
S8030	B	SOM2	KMC2
S8111	B	SOM2	KMC2
S8147	B	SOM2	KMC1
S8149	B	SOM2	KMC1
S8198	B	SOM2	KMC2
S8204	B	SOM2	KMC1
S8216	B	SOM2	KMC1
S3009	A	SOM1	KMC2
S5137	A	SOM1	KMC2
S5181	A	SOM1	KMC2
S7009	A	SOM1	KMC2
S8028	A	SOM2	KMC2
S8088	A	SOM1	KMC2
S8171	A	SOM1	KMC2
S8208	A	SOM1	KMC2
S8213	A	SOM2	KMC1
S8224	A	SOM1	KMC2
S8225	A	SOM1	KMC2
S8230	A	SOM1	KMC2

- Overall, there is less agreement between clustering methods for the LPV-LTWC than the EFV-LTWC
- A third method was employed for the LPV-LTWC (SOM) to expand the cross-referencing
- This may be due to sample size, inherently less metabolic variation in the LPV-LTWC, or that the variation is too individual to form consistent groups at this sample size

Comparison of hierarchical clustering LPV-LTWC metabotypes

(Relates to Fig 5)

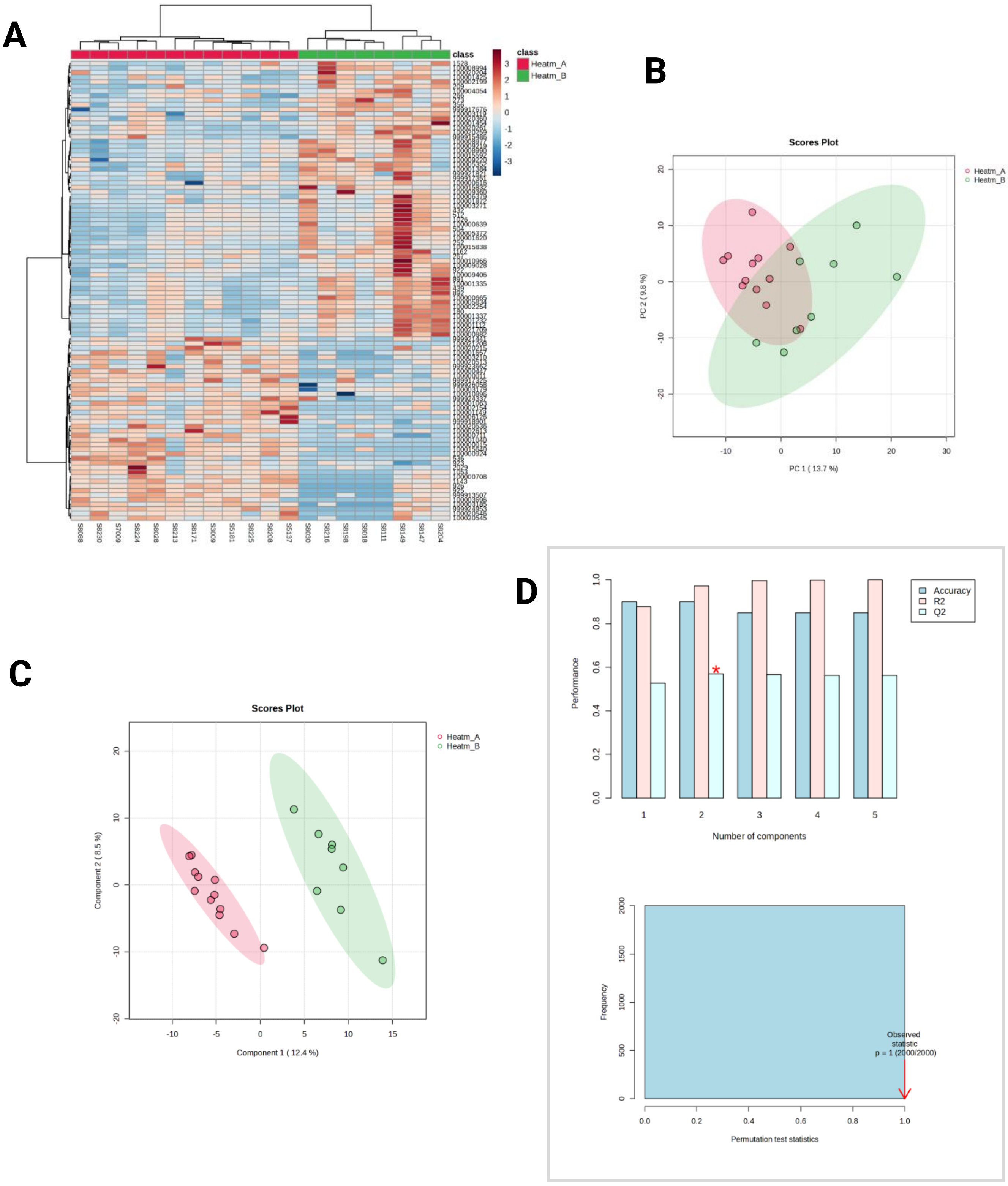

Figure S19 **A**: HCA heatmap, showing that there are some metabolic differences between the identified clusters, but that this is not consistent in all individuals. None of these differences were significant in KWD with FDR<0.05. **B**: PCA showing some separation between the LPV-LTWC HCA clusters, but also intra-group variability. **C and D**: The PLS-DA model showed borderline validation based on cross-validation, but did not validate based on permutation testing, likely due to the small sample size. On the balance of these findings, the LPV-LTWC metabotypes analysis was not pursued.

Data S7:

(Relates to Fig 3)

Information on the possible identities of the unannotated metabolites in the 5-metabolite model distinguishing EFV-CLWH and LPV-CLWH

1. **Spearman rank correlations with all other metabolites**
   This was done to determine which types of metabolites these metabolites share patterns, and therefore possibly pathways, with.
2. **Previously reported associations or correlations**

Metabolite X-17357

- Based on correlations in the CHANGES dataset and previously reported associations, metabolite X-17357 is most likely related to steroid and/or bile acid metabolism.

Table S20 Metabolites showing at least moderate (rho>0.5) correlation with X-17357

CHEMID	Spearman's rho	t-stat	p-value	FDR	Metabolite	Sub-pathway
999917357	0.78316	1076600	1.62E-65	9.91E-63	X-12844	Unannotated
999912844	0.68215	1578100	8.84E-44	3.60E-41	metabolonic lactone sulfate	Partially Characterized Molecules
100021331	0.63048	1834700	9.28E-36	2.83E-33	X-24556	Unannotated
999924556	0.61484	1912400	1.28E-33	3.12E-31	cortolone glucuronide (1)	Corticosteroids
100015971	0.61023	1935200	5.18E-33	1.05E-30	X-11444	Unannotated
999911444	-0.60693	7978600	1.39E-32	2.42E-30	glycohyocholate	Secondary Bile Acid Metabolism
100004083	0.59935	1989300	1.29E-31	1.96E-29	4-oxo-retinoic acid	Vitamin A Metabolism
100002825	0.5909	2031200	1.44E-30	1.95E-28	androsterone sulfate	Androgenic Steroids
100001073	0.58002	2085200	2.90E-29	3.54E-27	X-24418	Unannotated
999924418	0.55885	2190300	7.32E-27	8.12E-25	epiandrosterone sulfate	Androgenic Steroids
100001287	0.5481	2243700	1.05E-25	1.07E-23	androstenediol (3alpha, 17alpha) monosulfate (3)	Androgenic Steroids
100002027	0.54326	2267800	3.37E-25	3.16E-23	X-16935	Unannotated
999916935	-0.51992	7546600	7.25E-23	6.32E-21	taurohyocholate*	Secondary Bile Acid Metabolism
100004084	-0.51862	7540100	9.65E-23	7.85E-21	tauro-beta-muricholate	Primary Bile Acid Metabolism
100001250	0.50888	2438400	7.98E-22	6.09E-20	hydroxy-CMPF*	Fatty Acid, Dicarboxylate
100019794	-0.50715	7483100	1.16E-21	8.29E-20	methionine sulfone	Methionine, Cysteine, SAM and Taurine Metabolism

- Based on these associations, metabolite X-17357 correlates most frequently with steroid metabolites and bile acids, making it likely to be a lipid-related metabolite.

Table S21 Previously reported associations with X-17357

**Associated genes**		
**Gene**	**Gene name**	**Context and reference**
HSDD17B12	Hydroxysteroid 17-beta dehydrogenase 12	Involved in fatty acid elongation (chronic kidney disease[1] and in healthy Finnish men[2])
TMEM182	Transmembrane protein 182	Negative regulation of myogenesis and skeletal muscle regeneration
AKR1D1	Aldo-keto reductase family member D1 (Human delta(4)-3-oxosteroid 5-beta-reductase)	Catalyses 5-beta-reduction of bile acid intermediates and steroid hormones carrying a delta(4)-3-one structure[2]
**Other associations**		
Otto *et. al.* 2020[3]	Sex-specific associations with visceral adipose tissue volume in urine	
Quell *et. al.* 2017[4]	Possible involvement in dehydrogenase reactions	
Sun *et. al.* 2025[5]	Associations with lung cancer via immune receptors	

Metabolite X-16935

- This metabolite has previously been found to be lowered in PLWH who had been on ART for more than five years relative to healthy controls. Within the PLWH group, it was also significantly lower in the group on a Tenofovir/Lamivudine/Efavirenz regimen compared to those on a Zidovudine/Lamivudine/Nevirapine regimen[6].
- Our results confirm this finding — metabolite X-16935 was significantly lower in EFV-CLWH than LPV-CLWH and UCs (these groups had similar levels). This suggests that the alteration in this metabolite is a direct consequence of EFV (as the LPV group also received lamivudine). Given that it was detected in these other groups, at a higher level than in the EFV group, it is not a derivative of the EFV molecule itself.
- Based on correlations in the CHANGES dataset and previously reported associations, metabolite X-16935 is likely related to steroid and/or fatty acid metabolism, and may have a signalling role related to the energetic effects of EFV, as described under comparison 3.

Table S22 Metabolites showing at least moderate (rho>0.5) correlation with X-16935

CHEMID	Spearman's rho	t-stat	p-value	FDR	Metabolite	Sub-pathway
100021331	0.77097	21.245	2.61E-62	1.59E-59	metabolonic lactone sulfate	Partially Characterized Molecules
999921339	0.6594	15.393	4.72E-40	1.92E-37	X-21339	Unannotated
100004635	-0.65332	-15.145	4.13E-39	1.06E-36	methionine sulfone	Methionine, Cysteine, SAM and Taurine Metabolism
100002027	0.65317	15.139	4.36E-39	1.06E-36	androstenediol (3alpha, 17alpha) monosulfate (3)	Androgenic Steroids
999912456	0.64222	14.704	1.91E-37	3.88E-35	X-12456	Unannotated
999911880	0.62333	13.99	9.15E-35	1.59E-32	X-11880	Unannotated
100001073	0.61595	13.722	9.10E-34	1.39E-31	androsterone sulfate	Androgenic Steroids
999924556	0.61531	13.699	1.11E-33	1.50E-31	X-24556	Unannotated
100019794	0.61441	13.667	1.46E-33	1.78E-31	hydroxy-CMPF*	Fatty Acid, Dicarboxylate
100001287	0.59963	13.15	1.19E-31	1.32E-29	epiandrosterone sulfate	Androgenic Steroids
100021324	0.58932	12.802	2.24E-30	2.28E-28	3,5-dichloro-2,6-dihydroxybenzoic acid	Chemical
999911372	0.57728	12.407	6.07E-29	5.69E-27	X-11372	Unannotated
100015967	0.57479	12.327	1.18E-28	1.03E-26	carotene diol (2)	Vitamin A Metabolism
999917357	0.56258	11.942	2.84E-27	2.31E-25	X-17357	Unannotated
999921467	0.52911	10.943	9.19E-24	7.01E-22	X-21467	Unannotated

Table S23 Previously reported associations with X-16935

Associated genes		
**Gene**	**Gene name**	**Context and reference**
SLCO1B1	Solute carrier organic anion transporter family member 1B1	Transport of eicosanoids, thyroid hormones, and conjugated steroids (association found in healthy Finnish men)[2]
ACOT4, ACOT2	Acyl-CoA thioesterase 4 and 2	Hydrolysis of acyl-CoAs to free fatty acid and CoA (maintains free fatty acid:CoA ratio) (association found in healthy Finnish men)[2]
SLC27A2 / VLACS	Solute carrier family 27 member 2 / Very long chain acyl-CoA synthetase	Catalyses the activation of very long chain fatty acids to Co-A esters in the peroxisomes (association found in healthy Finnish men)[2]
JADRR, LINC03140	JADE1 adjacent regulatory RNA, long intergenic non-protein coding RNA 3140	Association found in healthy Asian adults[7]

DAPK1, LINC02872	Death associated protein kinase 1, long intergenic non-protein coding RNA 2872	DAPK1 – positive mediator of INF-gamma-induced programmed cell death, also TGF-ß-mediated apoptosis (mitochondrial) (association found in healthy Asian adults[7])
	**Other associations**	
Lou *et. al.* 2023[8]	Intake of deep-fried foods	
Al-Sayegh *et. al.* 2024[9]	Lower odds of lung cancer among former smokers	
Quell *et. al.* 2017[4]	Potentially involved in dehydrogenation reactions	

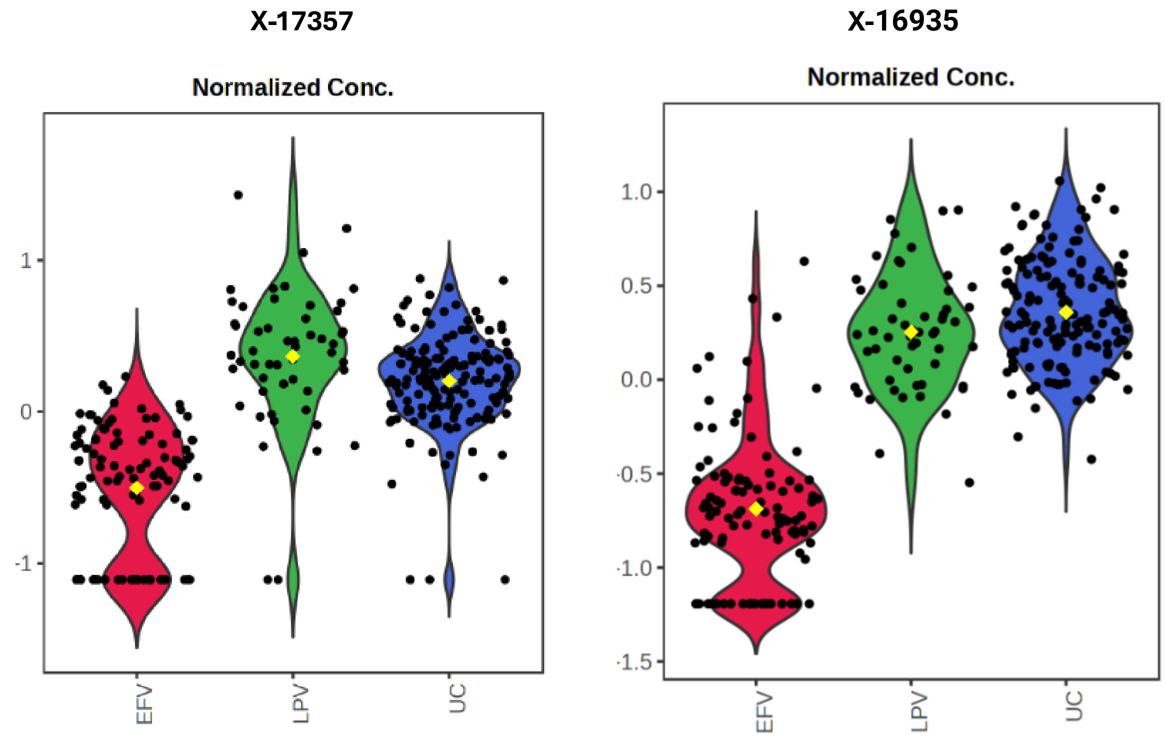

Figure S20 Violin plots of these metabolites, showing the EFV-specific pattern, which was also seen in metabolonic lactone sulfate.

References

[1] Schlosser P, Cheng Y, Gerasimova A, Wiese S, Maruani A, Vukojevic V, et al. Genetic studies of paired metabolomes reveal enzymatic and transport processes at the interface of plasma and urine. Nat Genet. 2023 Jun;55(6):995-1008. doi: 10.1038/s41588-023-01409-8.

[2] Yin X, Chan LS, Bose D, Jackson AU, VandeHaar P, Locke AE, et al. Genome-wide association studies of metabolites in Finnish men identify disease-relevant loci. Nat Commun. 2022 Mar 28;13(1):1644. doi: 10.1038/s41467-022-29143-5

[3] Otto L, Budde K, Kastenmüller G, Kaul A, Völker U, Völzke H, et al. Associations between adipose tissue volume and small molecules in plasma and urine among asymptomatic subjects from the general population. Sci Rep. 2020 Jan 30;10(1):1487. doi: 10.1038/s41598-020-58430-8.

[4] Quell JD, Römisch-Margl W, Colombo M, Krumsiek J, Evans AM, Mohney R, et al. Automated pathway and reaction prediction facilitates in silico identification of unknown metabolites in human cohort studies. J Chromatogr B Analyt Technol Biomed Life Sci. 2017 Dec 15;1071:58-67. doi: 10.1016/j.jchromb.2017.04.002.

[5] Sun T, Chen S, Liu Z, Hu Y, Sun Y, Wang L. Causal association of blood metabolites, immune cells, and lung cancer: A mediation Mendelian randomization study. Medicine (Baltimore). 2025 Apr 4;104(14):e42053. doi: 10.1097/MD.0000000000042053.

[6] Babu H, Sperk M, Ambikan AT, Rachel G, Viswanathan VK, Tripathy SP, et al. Plasma Metabolic Signature and Abnormalities in HIV-Infected Individuals on Long-Term Successful Antiretroviral Therapy. Metabolites. 2019;9(10):210.

[7] Sadhu N, Dalan R, Jain PR, Lee CJM, Pakkiri LS, Tay KY, et al. Metabolome-wide association identifies ferredoxin-1 (FDX1) as a determinant of cholesterol metabolism and cardiovascular risk in Asian populations. Nat Cardiovasc Res. 2025 May;4(5):567-583. doi: 10.1038/s44161-025-00638-w.

[8] Low DY, Mina TH, Sadhu N, Wong KE, Jain PR, Dalan R, et al. Metabolic variation reflects dietary intake in a multi-ethnic Asian population [Preprint]. medRxiv. 2023 Dec 04 [cited 2026 Feb 04]. doi: 10.1101/2023.12.04.23299350.

[9] Al-Sayegh T, Wen W, Wu J, Zheng W, Shu XO, Cai Q. Abstract LB136: Global metabolomics identified promising biomarkers for lung cancer risk among former smokers. Cancer Res. 2024 Apr 1;84(7 Suppl):LB136. doi: 10.1158/1538-7445.AM2024-LB136.
